# Novel synthesis of pseudopeptides bearing a difluoromethyl group by Ugi reaction and desulfanylation

**DOI:** 10.3762/bjoc.7.123

**Published:** 2011-08-08

**Authors:** Jingjing Wu, Hui Li, Song Cao

**Affiliations:** 1 Shanghai Key Laboratory of Chemical Biology, School of Pharmacy, East China University of Science and Technology, Shanghai 200237, China

**Keywords:** difluoromethyl functionality, *gem*-difluoromethylene-containing acid, pseudopeptides, reductive cleavage, Ugi reaction

## Abstract

Thirteen difluoromethyl-containing pseudopeptides were synthesized by Ugi reaction using the novel building block 2,2-difluoro-2-(phenylthio)acetic acid (**2**) as one component, followed by removal of the phenylsulfanyl protecting group in the presence of tributyltin hydride and azobisisobutyronitrile.

## Introduction

Fluorinated amino acids and pseudopeptides have increasingly attracted attention in recent years [1–5]. The selective incorporation of fluorine-containing groups, such as trifluoromethyl, difluoromethyl and difluoromethylene, into peptides or peptidomimetics often drastically alters the chemical, physical, and biological properties of the parent compounds [6–9]. Nowadays, difluoromethyl-containing compounds are increasingly being applied in pharmaceuticals and agrochemicals [10–12]. It is reported that difluoromethyl functionality (CF_2_H) is isosteric and isopolar to the hydroxyl group and can behave as a hydrogen donor through hydrogen bonding [13].

**Figure 1:**
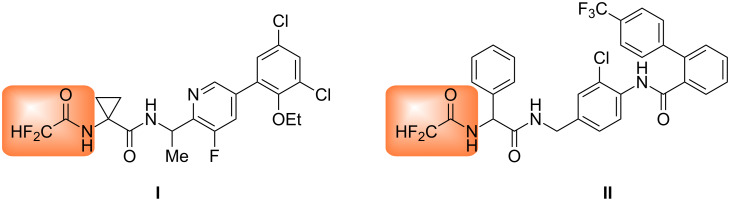
Two examples of bioactive pseudopeptides bearing a CF_2_H group.

However, to date, most fluorine-containing peptide modifications involve the introduction of trifluoromethyl or difluoromethylene into molecules [14–18]. Only a few examples have been reported of the preparation and bioassay of pseudopeptides and peptidomimetics bearing difluoromethyl groups. For example, compound **I** can act as bradykinin B1 antagonist or inverse agonist and can be used in the prevention of inflammation and pain [19]. Compound **II** is an inhibitor of microsomal triglyceride transfer protein (MTP) and useful for the treatment of obesity and atherosclerosis (Figure 1) [20].

**Scheme 1:**
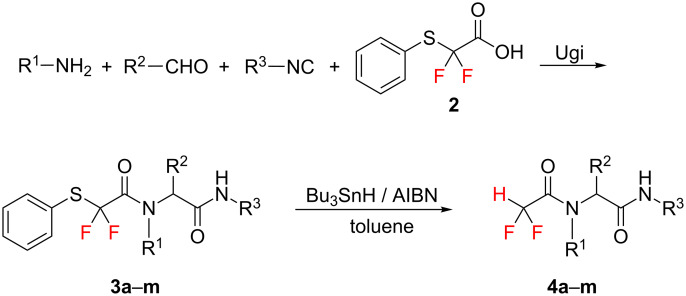
Synthesis of difluoromethyl-containing pseudopeptides (**4a**–**m)** by Ugi reaction and desulfanylation.

Among the protocols for the preparation of pseudopeptide derivatives, the Ugi four-component reaction offers significant advantages over conventional linear-step synthesis [21]. Various fluorinated building blocks have been used in the Ugi four-component reaction to construct a fluorinated compound library [22–25]. Our group has always been interested in developing efficient methods for the preparation of difluoromethyl-containing compounds through multicomponent reactions [26–30]. Recently, we reported a novel and general strategy for the construction of a difluoromethyl compound library, and we further illustrated this strategy by application to the synthesis of CF_2_H-bearing pseudopeptides and 1,2,3-triazoles through Ugi and click reaction, respectively [27,30]. In continuation of our interest in the synthesis of diverse difluoromethyl-containing pseudopeptides, we herein report a novel and efficient synthesis of difluoromethyl-containing pseudopeptides through Ugi reaction, with *gem*-difluoromethylene-containing acid as a key component, followed by reductive cleavage of the phenylsulfanyl group (Scheme 1).

## Results and Discussion

**Scheme 2:**
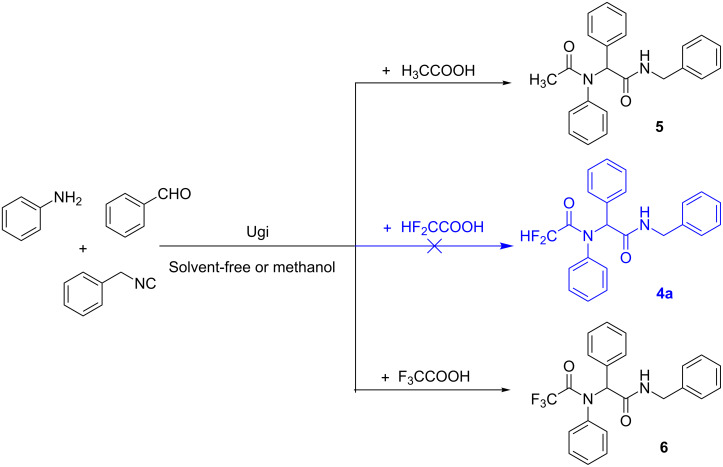
The Ugi reaction of aniline, benzaldehyde, (isocyanomethyl)benzene with acetic acid, difluoroacetic acid and trifluoroacetic acid in methanol or under solvent-free conditions.

For the purpose of screening novel bioactive compounds, we recently prepared a variety of diverse difluoromethyl-containing pseudopeptides. In our initial experiments, we tried to use difluoroacetic acid as one component to undergo Ugi reaction to prepare difluoromethyl-containing pseudopeptides. Unfortunately, the anticipated difluoromethyl-containing product **4a** was not obtained (Scheme 2). Although there are a few examples of acetic acid and trifluoroacetic acid acting as substrates in an Ugi reaction [24,31], up to now, no literature was found concerning the use of difluoroacetic acid as one of the components in the Ugi reaction. For a comparative study, acetic acid and trifluoroacetic acid served as the substrates for the Ugi reaction under the same reaction conditions as those used for the difluoroacetic acid, and the results indicated that the reaction proceeded efficiently regardless of reaction conditions, and the Ugi products (**5** and **6**) were obtained in good yields. The hydrogen atom next to the CF_2_ group seems to influence the formation of Ugi product.

**Scheme 3:**
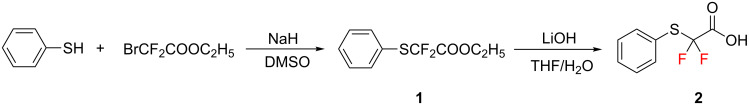
Synthesis of 2,2-difluoro-2-(phenylthio)acetic acid (**2**).

In previous studies, we developed a synthetic methodology to prepare functionalized small molecules having a CF_2_H group [27]. In this work, we first synthesized a protected difluoro-containing building block, 2,2-difluoro-2-(phenylthio)acetic acid (**2**). The synthesis of compound **2** is illustrated in Scheme 3. The ethyl 2,2-difluoro-2-(phenylthio)acetate (**1**) was readily prepared by the reaction of ethyl bromodifluoroacetate and thiophenol according to the known procedure [32]. The novel difluorinated acid **2** was obtained by hydrolysis of the ester under basic condition in nearly quantitative yield.

After successful synthesis of the protected functionalized CF_2_ building block **2**, we tried to use it as one of the components in the preparations of the difluoromethylene-containing pseudopeptides by Ugi reaction. Indeed, the reaction of aniline, benzaldehyde, (isocyanomethyl)benzene with **2** proceeded efficiently under solvent-free conditions. Finally, we removed the protecting group (PhS) with Bu_3_SnH/AIBN according to our previous research, and the desired difluoromethyl-containing pseudopeptide was successfully obtained [27].

To demonstrate the scope of the method, several different substituted anilines, substituted benzaldehydes, isocyanides and this novel difluorinated building block **2** were subjected to Ugi reaction under solvent-free conditions, followed by reductive cleavage of the phenylsulfanyl group. It was found that both Ugi reaction and desulfanylation proceeded smoothly for all substrates used to give the corresponding difluoromethylene-containing and difluoromethyl-containing pseudopeptides (**3a**–**m** and **4a**–**m**) in good yields (Table 1).

**Table 1:** Synthesis of difluoromethylene-containing pseudopeptides (**3a**–**m**) and difluoromethyl-containing pseudopeptides (**4a**–**m**).

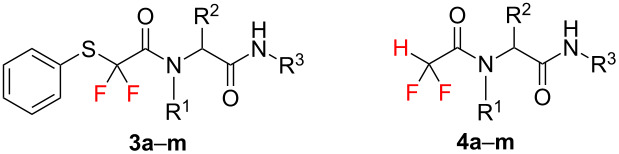					

Entry	R^1^	R^2^	R^3^	**3**	**4**
				Yield (%)^a^	Yield (%)^a^

a	Ph	Ph	Bn	82	75
b	2-MePh	Ph	Bn	78	68
c	2-MePh	4-MePh	Bn	75	74
d	4-MeOPh	Ph	Bn	79	75
e	Ph	4-MeOPh	Bn	78	70
f	2-MePh	4-MeOPh	Bn	74	67
g	4-MePh	4-MeOPh	Bn	72	78
h	4-FPh	4-MeOPh	Bn	70	71
i	Ph	4-FPh	Bn	77	75
j	2-MePh	4-FPh	Bn	70	69
k	4-MeOPh	4-FPh	Bn	72	74
l	Ph	Ph	Ph	68	66
m	Ph	4-MeOPh	Ph	66	60

^a^Isolated yield.

## Conclusion

In summary, we have developed a novel and efficient protocol for the synthesis of CF_2_H-containing pseudopeptides by Ugi reaction of substituted anilines, benzaldehyde, isocyanides and the novel building block 2,2-difluoro-2-(phenylthio)acetic acid (**2**), followed by the cleavage of the phenylsulfanyl group.

## Experimental

### General

All reagents were of analytic grade, obtained from commercial suppliers and were used without further purification. Melting points were measured in an open capillary using Büchi melting point B-540 apparatus and are uncorrected. ^1^H NMR and ^13^C NMR spectra were recorded on a Bruker AM-400 spectrometer (400 MHz and 100 MHz, respectively) using TMS as internal standard. The ^19^F NMR were obtained using a Bruker AM-400 spectrometer (376 MHz) and the ^19^F NMR were measured with external CF_3_CO_2_H as standard. Gas chromatography-mass spectra (GC-MS) were recorded on HP 5973 MSD with 6890 GC. High resolution mass spectra (HRMS) were recorded under electron impact conditions using a MicroMass GCT CA 055 instrument and recorded on a MicroMass LCTTM spectrometer. Column chromatography was carried out with Merck 60 (230–400 mesh) silica gel.

#### General procedure for compounds 3a–m

To a stirred amine (1 mmol), the aldehyde (1 mmol) was added in portions for about 5 min. The mixture was stirred for 30 min at rt. Then, the reaction mixture was heated to 60 °C, and isocyanide (1 mmol) and 2,2-difluoro-2-(phenylthio)acetic acid (**2**) (1 mmol) were added. Stirring was continued at 60 °C for 1 h (TLC). The crude residue was purified by chromatography to give the desired products **3**.

#### General procedure for compounds 4a–m

Bu_3_SnH (0.58 g, 2 mmol) was added under argon atmosphere to a solution of **3** (1 mmol) in dry toluene (3 mL). Deoxygenation was continued for 5 min. Azobisisobutyronitrile (AIBN) (0.02 g, 0.1 mmol) was added and the solution was heated at reflux for 9 h (TLC). The mixture was concentrated under reduced pressure and the residue was dissolved in EtOAc (5 mL). The solution was stirred with KF/H_2_O (15 mg/0.15 mL) for 3 h and extracted with EtOAc (3 × 20 mL). The combined organic phases were washed successively with water (20 mL) and brine (20 mL), and dried over anhydrous Na_2_SO_4_. After removal of the solvent, the crude product was purified by chromatography to give the desired products **4**.

## Supporting Information

File 1 Experimental procedures and compound characterization.
