# Supplementary material for: Novel synthesis of pseudopeptides bearing a difluoromethyl group by Ugi reaction and desulfanylation
Source: Beilstein J Org Chem. 2011 Aug 8;7:1070–4. doi: 10.3762/bjoc.7.123 (PMC3170163; doi:10.3762/bjoc.7.123)

Supporting Information

for

Novel synthesis of pseudopeptides bearing a difluoromethyl group by Ugi reaction and desulfanylation

Jingjing Wu, Hui Li and Song Cao*

Address: Shanghai Key Laboratory of Chemical Biology, School of Pharmacy, East China University of Science and Technology, Shanghai 200237, China

Email: Song Cao* - scao@ecust.edu.cn

* Corresponding author

Experimental procedures and compound characterization

Contents

General …………………………………………………………………….. S2

Preparation and physical data of compounds **1-2**...S2

Preparation and physical data of compounds **3**………………………............S2–5

Preparation and physical data of compounds **4**……………………………...S5–8

Preparation and physical data of compound **5**… ……………………………….S8

Preparation and physical data of compound **6**… ……………..……….……….S8

References...……………………………………………...……………….S8

NMR spectra and MS spectra for all products…………………………..…….S9–56

General

All reagents were of analytic grade, obtained from commercial suppliers and used without further purification. Melting points were measured in an open capillary using Büchi melting point B-540 apparatus and are uncorrected. ^{1}H NMR and ^{13}C NMR spectra were recorded on a Bruker AM-400 spectrometer (400 MHz and 100 MHz, respectively) using TMS as internal standard. The ^{19}F NMR were obtained using a Bruker AM-400 spectrometer (376 MHz) and the ^{19}F NMR were measured with external CF_3CO_2H as the standard. Gas chromatography-mass spectra (GC-MS) were recorded on HP 5973 MSD with 6890 GC. High resolution mass spectra (HRMS) were recorded under electron impact conditions using a MicroMass GCT CA 055 instrument and recorded on a MicroMass LCTTM spectrometer. Column chromatography was carried out with Merck 60 (230–400 mesh) silica gel.

Preparation of ethyl 2,2-difluoro-2-(phenylthio)acetate (**1**)

To a solution of thiophenol (1.10 g, 10 mmol) in DMSO (10 mL) was added NaH (0.26 g, 11 mmol) at rt. After the mixture was stirred for 1 h, ethyl bromodifluoroacetate (2.23 g, 11 mmol) was added to the solution. The mixture was stirred at the same temperature for 19 h (TLC) and quenched with aqueous NH_4Cl solution and extracted with CH_2Cl_2. The organic layer was washed successively with water and brine, dried over anhydrous Na_2SO_4, and evaporated under reduced pressure. The residue was purified by chromatography on a silica gel column to give **1**. Yield: 83%. GC-MS: *m/z* = 232, 159, 109, 77.

Preparation of 2,2-difluoro-2-(phenylthio)acetic acid (**2**)

A solution of **1** (1.66 g, 5 mmol) in THF (15 mL) was mixed with a solution of LiOH (0.36 g, 15 mmol) in H_2O (15 mL). After the mixture was stirred at rt for 1 h (TLC), THF was removed under reduced pressure and the solution was washed with ether (3 × 20 mL). The solution was adjusted to pH 3.0 with concentrated HCl and was extracted with ether. The combined ether solution was dried over anhydrous Na_2SO_4. The solvent was removed to give **2** without further purification (0.80 g, 93%). Colorless liquid. ^{1}H NMR (400 MHz, $CDCl_3$): δ 9.96 (s, 1H), 7.67–7.65 (d, J = 7.7 Hz, 2H), 7.53–7.42 (m, 3H); ^{13}C NMR (100 MHz, $CDCl_3$): δ 166.0 (t, $^{2}J_{CF}$ = 33.6 Hz), 136.8, 131.0, 129.5, 124.2, 119.6 (t, $^{1}J_{CF}$ = 287.3 Hz); ^{19}F NMR (376.5 MHz, $CDCl_3$): δ −83.6 (s, 2F).

Preparation of isocyanides

Isocyanides were synthesized according to literature procedures [1].

General procedure for compounds **3a**–**m**

To a stirred amine (1 mmol), aldehyde (1 mmol) was added in portions for about 5 min. The mixture was stirred for 30 min again at rt. Then, the reaction mixture was heated to 60 °C, isocyanide (1 mmol) and 2,2-difluoro-2-(phenylthio)acetic acid (**2**) (1 mmol) were added. Stirring was continued at 60 °C for 1 h (TLC). The crude residue was purified by chromatography to give the desired products **3**.

N-(2-(benzylamino)-2-oxo-1-phenylethyl)-2,2-difluoro-N-phenyl-2-(phenylthio)acetamide (3a):

White solid. Yield: 82%. Mp: 176.2–177.1°C, ^{1}H NMR (400 MHz, $CDCl_3$): δ 7.71–7.32 (m, 4H), 7.19–7.14 (m, 12H), 7.1–6.65 (m, 4H), 5.99 (s, 1H), 5.93 (s, 1H), 4.54–4.43 (m, 2H); ^{13}C NMR (100 MHz, $CDCl_3$): δ 168.3, 161.7 (t, $^2J_{CF}$ = 27.2 Hz), 137.8, 137.1, 136.9, 132.8, 131.0, 130.6, 130.0, 129.0, 128.9, 128.6, 128.5, 128.0, 127.9, 127.6, 127.4, 125.7, 123.7 (t, $^1J_{CF}$ = 293.7 Hz), 67.7, 43.8; ^{19}F NMR (376 MHz, $CDCl_3$): δ (−71.3)–(−71.4) (m, 2F).

N-(2-(benzylamino)-2-oxo-1-phenylethyl)-2,2-difluoro-2-(phenylthio)-N-o-tolylacetamide (3b):

White solid. Yield: 78%. Mp: 132.0–132.9 °C, ^{1}H NMR (400 MHz, $CDCl_3$): δ 7.64–7.29 (m, 8H), 7.27–7.11 (m, 10H), 6.95–6.92 (m, 1H), 5.87 (s, 1H), 5.83 (s, 1H), 4.54–4.52 (m, 2H), 1.90 (s, 3H); ^{13}C NMR (100 MHz, $CDCl_3$): δ 168.4, 162.2 (t, $^2J_{CF}$ = 27.1 Hz), 138.0, 137.9, 137.0, 135.9, 132.0, 131.8, 131.1, 130.1, 130.0, 129.2, 1291, 128.9, 128.6, 128.3, 127.5, 127.4, 125.8, 125.7, 123.7 (t, $^1J_{CF}$ = 293.9Hz), 68.0, 43.8, 18.0; ^{19}F NMR (376 MHz, $CDCl_3$): δ −73.0 (d, J_{FF} = 220.8 Hz, 1F), −74.2 (d, J_{FF} = 220.8 Hz, 1F).

N-(2-(benzylamino)-2-oxo-1-p-tolylethyl)-2,2-difluoro-2-(phenylthio)-N-o-tolylacetamide (3c):

White solid. Yield: 75%. Mp: 156.1–157.5 °C, ^{1}H NMR (400 MHz, $CDCl_3$): δ 7.64–6.95 (m, 18H), 5.86 (t, J = 3.9 Hz, 1H), 5.76 (s, 1H), 4.57–4.47 (m, 2H), 2.29 (s, 3H), 1.92 (s, 3H); ^{13}C NMR (100 MHz, $CDCl_3$): δ 168.7, 162.1 (t, $^2J_{CF}$ = 27.1 Hz), 139.1, 138.1, 137.0, 136.0, 131.8, 131.0, 130.7, 130.2, 130.0, 129.8, 129.0, 128.9, 128.6, 127.6, 127.5, 126.4, 125.9, 125.7, 124.0 (t, $^1J_{CF}$ = 294.1 Hz), 67.8, 43.8, 21.2, 18.1; ^{19}F NMR (376 MHz, $CDCl_3$): δ −72.9 (d, J_{FF} = 220.7 Hz, 1F), −74.2 (d, J_{FF} = 220.7 Hz, 1F).

N-(2-(benzylamino)-2-oxo-1-phenylethyl)-2,2-difluoro-N-(4-methoxyphenyl)-2-(phenylthio)acet-amide (3d):

White solid. Yield: 79%. Mp: 142.2–145.0 °C, ^{1}H NMR (400 MHz, $CDCl_3$): δ 7.73–7.29 (m, 9H), 7.27–6.70 (m, 10H), 6.00 (t, J = 5.4 Hz, 1H), 5.94 (s, 1H), 4.57–4.47 (m, 2H), 3.75 (s, 3H); ^{13}C NMR (100 MHz, $CDCl_3$): δ 168.6, 161.7 (t, $^2J_{CF}$ = 27.4 Hz), 160.0, 138.0, 137.1, 137.0, 132.1, 131.2, 130.0, 129.0, 128.7, 128.5, 128.0, 127.6, 127.4, 125.8, 124.8, 123.8 (t, $^1J_{CF}$ = 293.6 Hz), 113.9, 67.1, 55.2, 43.8; ^{19}F NMR (376 MHz, $CDCl_3$): δ −70.9 (d, J_{FF} = 220.8 Hz, 1F), −71.5 (d, J_{FF} = 220.9 Hz, 1F).

N-(2-(benzylamino)-1-(4-methoxyphenyl)-2-oxoethyl)-2,2-difluoro-N-phenyl-2-(phenylthio)acet-amide (3e):

White solid. Yield: 78%. Mp: 140.3–142.9 °C, ^{1}H NMR (400 MHz, $CDCl_3$): δ 7.67–7.30 (m, 8H), 7.27–6.51 (m, 11H), 5.97 (s, 1H), 5.95 (s, 1H), 4.57–4.47 (m, 2H), 3.73 (s, 3H); ^{13}C NMR (100 MHz, $CDCl_3$): δ 168.3, 162.1 (t, $^2J_{CF}$ = 27.2 Hz), 159.4, 137.8, 136.9, 133.0, 132.1, 130.7, 130.0, 129.6, 129.0, 128.9, 128.7, 128.6, 127.6, 127.5, 125.9, 123.8 (t, $^1J_{CF}$ = 293.8 Hz), 113.0, 67.5, 55.3, 43.9; ^{19}F NMR (376 MHz, $CDCl_3$): δ −70.9 (d, J_{FF} = 221.0 Hz, 1F), −71.6 (d, J_{FF} = 221.0 Hz, 1F).

N-(2-(benzylamino)-1-(4-methoxyphenyl)-2-oxoethyl)-2,2-difluoro-2-(phenylthio)-N-o-tolylacet-amide (3f):

White solid. Yield: 74%. Mp: 154.0–155.1 °C, ^{1}H NMR (400 MHz, $CDCl_3$): δ 7.64–7.30 (m, 8H), 7.26–6.68 (m, 10H), 5.90 (t, J = 4.5 Hz, 1H), 5.82 (s, 1H), 4.53–4.51 (m, 2H), 3.75 (s, 3H), 1.89 (s, 3H); ^{13}C NMR (100 MHz, $CDCl_3$): δ 168.7, 162.1 (t, $^2J_{CF}$ = 27.1 Hz), 160.1, 138.1, 138.0, 137.0,

135.8, 132.4, 131.8, 130.2, 130.0, 129.1, 128.9, 128.6, 127.5, 127.4, 125.8, 125.7, 123.9 (t, $^{1}J_{CF}$ = 294.1 Hz), 114.3, 113.6, 67.2, 55.2, 43.8, 18.1; ^{19}F NMR (376 MHz, $CDCl_3$): δ −73.0 (d, J_{FF} = 220.6 Hz, 1F), −74.2 (d, J_{FF} = 220.6 Hz, 1F).

***N*-(2-(benzylamino)-1-(4-methoxyphenyl)-2-oxoethyl)-2,2-difluoro-2-(phenylthio)-*N*-*p*-tolylacetamide (3g):**

White solid. Yield: 72%. Mp: 147.1–147.7 °C, ^{1}H NMR (400 MHz, $CDCl_3$): δ 7.54–7.30 (m, 8H), 7.27–6.56 (m, 10H), 6.02 (s, 1H), 5.91 (s, 1H), 4.56–4.46 (m, 2H), 3.76 (s, 3H), 2.27 (s, 3H); ^{13}C NMR (100 MHz, $CDCl_3$): δ 168.7, 161.8 (t, $^{2}J_{CF}$ = 27.2 Hz), 159.9, 138.4, 138.1, 136.9, 134.5, 132.1, 130.8, 130.0, 128.9, 128.6, 128.7, 127.6, 127.4, 125.9, 125.0, 124.0 (t, $^{1}J_{CF}$ = 293.7 Hz), 113.9, 67.1, 55.2, 43.8, 21.2; ^{19}F NMR (376 MHz, $CDCl_3$): δ −70.7 (d, J_{FF} = 220.9 Hz, 1F), −71.3 (d, J_{FF} = 221.0 Hz, 1F).

***N*-(2-(benzylamino)-1-(4-methoxyphenyl)-2-oxoethyl)-2,2-difluoro-*N*-(4-fluorophenyl)-2-(phenylthio)acetamide (3h):**

White solid. Yield: 70%. Mp: 162.3–163.2 °C, ^{1}H NMR (400 MHz, $CDCl_3$): δ 7.80–7.22 (m, 11H), 7.02–6.54 (m, 7H), 6.00 (s, 1H), 5.98 (t, J = 5.6 Hz, 1H), 4.56–4.45 (m, 2H), 3.76 (s, 3H); ^{13}C NMR (100 MHz, $CDCl_3$): δ 168.8, 162.2 (d, $^{1'}J_{CF}$ = 249.0 Hz), 161.8 (t, $^{2}J_{CF}$ = 27.2 Hz), 160.1, 138.0, 136.9, 133.5, 133.1, 132.7, 132.1, 130.1, 129.0, 128.7, 127.5, 127.4, 125.6, 124.5, 123.8 (t, $^{1}J_{CF}$ = 293.7 Hz), 114.6 (d, $^{2'}J_{CF}$ = 23.7 Hz), 114.0, 66.4, 55.2, 43.8; ^{19}F NMR (376 MHz, $CDCl_3$): δ −71.4 (s, 2F), (−112.3)–(−112.4) (m, 1F).

***N*-(2-(benzylamino)-1-(4-fluorophenyl)-2-oxoethyl)-2,2-difluoro-*N*-phenyl-2-(phenylthio)acetamide (3i):**

White solid. Yield: 77%. Mp: 184.9–186.0 °C, ^{1}H NMR (400 MHz, $CDCl_3$): δ 7.71–7.31 (m, 9H), 7.27–6.62 (m, 10H), 6.03 (s, 1H), 5.96 (s, 1H), 4.59–4.45 (m, 2H); ^{13}C NMR (100 MHz, $CDCl_3$): δ 168.3, 162.9 (d, $^{1'}J_{CF}$ = 249.3 Hz), 161.9 (t, $^{2}J_{CF}$ = 28.1 Hz), 137.8, 136.9, 136.7, 132.7, 132.6, 131.1, 130.1, 129.0, 128.7, 128.2, 127.9, 127.6, 127.5, 125.6, 123.7 (t, $^{1}J_{CF}$ = 293.8 Hz), 115.5 (d, $^{2'}J_{CF}$ = 21.6 Hz), 66.5, 43.8; ^{19}F NMR (376 MHz, $CDCl_3$): δ −71.4 (s, 2F), −111.7 (s, 1F).

***N*-(2-(benzylamino)-1-(4-fluorophenyl)-2-oxoethyl)-2,2-difluoro-2-(phenylthio)-*N*-*o*-tolylacetamide (3j):**

White solid. Yield: 70%. Mp: 162.3–163.2 °C, ^{1}H NMR (400 MHz, $CDCl_3$): δ 7.64–7.31 (m, 9H), 7.24–6.83 (m, 9H), 5.97 (s, 1H), 5.87 (s, 1H), 4.56–4.50 (m, 2H), 1.86 (s, 3H); ^{13}C NMR (100 MHz, $CDCl_3$): δ 168.3, 163.1 (d, $^{1'}J_{CF}$ = 249.6 Hz), 162.3 (t, $^{2}J_{CF}$ = 27.5 Hz), 137.9, 137.8, 137.0, 135.4, 133.0 (d, $^{3'}J_{CF}$ = 8.4 Hz), 131.8, 130.2, 130.1, 129.3, 128.9, 128.7, 127.5, 127.4, 125.8, 125.6, 123.8 (t, $^{1}J_{CF}$ = 293.2 Hz), 116.0, 115.2 (d, $^{2'}J_{CF}$ = 21.5 Hz), 66.7, 43.9, 18.0; ^{19}F NMR (376 MHz, $CDCl_3$): δ −73.3 (d, J_{FF} = 220.7 Hz, 1F), −74.3 (d, J_{FF} = 220.7 Hz, 1F), −111.5 (s, 1F).

***N*-(2-(benzylamino)-1-(4-fluorophenyl)-2-oxoethyl)-2,2-difluoro-*N*-(4-methoxyphenyl)-2-(phenylthio)acetamide (3k):**

White solid. Yield: 72%. Mp: 154.0–155.1 °C, ^{1}H NMR (400 MHz, $CDCl_3$): δ 7.63–7.29 (m, 8H), 7.28–6.48 (m, 10H), 6.01 (s, 1H), 5.97 (s, 1H), 4.57–4.47 (m, 2H), 3.75 (s, 3H); ^{13}C NMR (100 MHz, $CDCl_3$): δ 168.4, 162.9 (d, $^{1'}J_{CF}$ = 249.2 Hz), 162.1 (t, $^{2}J_{CF}$ = 27.0 Hz), 159.4, 137.8, 136.9, 132.7 (d,

$^{3'}J_{CF}$ = 8.3 Hz), 132.5, 132.2, 130.1, 129.1, 129.0, 128.7, 127.6, 127.5, 125.7, 123.7 (t, $^{1}J_{CF}$ = 293.6 Hz), 115.5 (d, $^{2'}J_{CF}$ = 21.6 Hz), 113.1, 66.1, 55.3, 43.8; ^{19}F NMR (376 MHz, $CDCl_3$): δ −71.4 (s, 2F), −111.8 (s, 1F).

2,2-Difluoro-*N*-(2-oxo-1-phenyl-2-(phenylamino)ethyl)-*N*-phenyl-2-(phenylthio)acetamide (3l):
White solid. Yield: 68%. Mp: 207.8–208.5 °C, ^{1}H NMR (400 MHz, $CDCl_3$): δ 7.79–7.31 (m, 13H), 7.27–7.02 (m, 7H), 6.67 (s, 1H), 6.11 (s, 1H); ^{13}C NMR (100 MHz, $CDCl_3$): δ 166.5, 162.0 (t, $^{2}J_{CF}$ = 27.6 Hz), 137.4, 136.9, 132.4, 131.1, 130.7, 130.1, 129.2, 129.0, 128.7, 128.6, 128.3, 127.9, 125.6, 124.6, 123.7 (t, $^{1}J_{CF}$ = 293.7 Hz), 120.9, 120.1, 68.2; ^{19}F NMR (376 MHz, $CDCl_3$): δ −71.4 (s, 2F).

2,2-Difluoro-*N*-(1-(4-methoxyphenyl)-2-oxo-2-(phenylamino)ethyl)-*N*-phenyl-2-(phenylthio)acetamide (3m):
White solid. Yield: 66%. Mp: 195.2–196.0 °C, ^{1}H NMR (400 MHz, $CDCl_3$): δ 7.79–6.74 (m, 19H), 6.65 (s, 1H), 6.11 (s, 1H), 3.77 (s, 3H); ^{13}C NMR (100 MHz, $CDCl_3$): δ 166.9, 161.9 (t, $^{2}J_{CF}$ = 27.4 Hz), 160.1, 137.5, 136.9, 136.8, 132.2, 131.2, 130.1, 128.9, 128.6, 128.2, 127.8, 125.7, 124.5, 124.3, 123.8 (t, $^{1}J_{CF}$ = 293.7 Hz), 120.1, 114.0, 67.5, 55.3; ^{19}F NMR (376 MHz, $CDCl_3$): δ −71.3 (s, 2F).

General procedure for Compounds **4a–m**

To a solution of **3** (1 mmol) in dry toluene (3 mL) was added Bu_3SnH (0.58 g, 2 mmol) under an argon atmosphere. Deoxygenation was continued for 5 min. Azobisisobutyronitrile (AIBN) (0.02 g, 0.1 mmol) was added and the solution was heated at reflux for 9 h (TLC). The mixture was concentrated under reduced pressure and the residue was dissolved in EtOAc (5 mL). The solution was stirred with KF/H_2O (15 mg/0.15 mL) for 3 h and extracted with EtOAc (3 × 20 mL). The organic phase was washed successively with water (20 mL) and brine (20 mL), dried over anhydrous Na_2SO_4. After solvent removal, the crude product was purified by chromatography to give desired products **4**.

***N*-(2-(benzylamino)-2-oxo-1-phenylethyl)-2,2-difluoro-*N*-phenylacetamide (4a):**
White solid. Yield: 75%. Mp: 187.0–187.8 °C, ^{1}H NMR (400 MHz, $CDCl_3$): δ 7.76–7.30 (m, 5H), 7.27–6.68 (m, 10H), 6.06 (s, 1H), 5.94 (t, J = 3.9 Hz, 1H), 5.74 (t, J_{HF} = 53.4 Hz, 1H), 4.59–4.47 (m, 2H); ^{13}C NMR (100 MHz, $CDCl_3$): δ 168.4, 162.3 (t, $^{2}J_{CF}$ = 27.4 Hz), 137.7, 136.4, 132.8, 130.8, 130.5, 129.2, 129.1, 128.7, 128.6, 127.6, 127.5, 105.9 (t, $^{1}J_{CF}$ = 246.1 Hz), 65.8, 43.9; ^{19}F NMR (376 MHz, $CDCl_3$): δ −122.9 (dd, J_{HF} = 53.3 Hz, J_{FF} = 320.2 Hz, 1F), −124.1 (dd, J_{HF} = 53.6 Hz, J_{FF} = 320.2 Hz, 1F); HRMS–ESI: $[M + Na]^+$ calcd for $C_{23}H_{20}F_2N_2O_2Na$, 417.1391; found, 417.1389.

***N*-(2-(benzylamino)-2-oxo-1-phenylethyl)-2,2-difluoro-*N*-*o*-tolylacetamide (4b):**
White solid. Yield: 75%. Mp: 187.0–187.8 °C, ^{1}H NMR (400 MHz, $CDCl_3$): δ 7.71–7.29 (m, 4H), 7.27–7.02 (m, 10H), 5.96 (s, 1H), 5.95 (s, 1H), 5.67 (t, J_{HF} = 53.6 Hz, 1H), 4.56–4.45 (m, 2H), 1.83 (s, 3H); ^{13}C NMR (100 MHz, $CDCl_3$): δ 168.5, 162.6 (t, $^{2}J_{CF}$ = 26.7 Hz), 138.1, 135.1, 131.9, 131.4, 130.9, 130.2, 129.8, 129.3, 128.7, 128.4, 127.5, 127.4, 127.0, 105.8 (t, $^{1}J_{CF}$ = 247.5 Hz), 66.1, 43.9, 17.6; ^{19}F NMR (376 MHz, $CDCl_3$): δ −123.8 (dd, J_{HF} = 33.0 Hz, J_{FF} = 301.3 Hz, 1F), −124.8 (dd, J_{HF} = 33.5 Hz, J_{FF} = 301.2 Hz, 1F); HRMS–ESI: $[M - H]^+$ calcd for $C_{24}H_{21}F_2N_2O_2$, 407.1571; found, 407.1569.

N-(2-(benzylamino)-2-oxo-1-p-tolylethyl)-2,2-difluoro-N-o-tolylacetamide (4c):
White solid. Yield: 74%. Mp: 179.6–180.5 °C, ^{1}H NMR (400 MHz, $CDCl_3$): δ 7.69–7.29 (m, 4H), 7.26–6.97 (m, 9H), 5.92 (s, 1H), 5.91 (s, 1H), 5.66 (t, J_{HF} = 53.6 Hz, 1H), 4.56–4.44 (m, 2H), 2.28 (s, 3H), 1.85 (s, 3H); ^{13}C NMR (100 MHz, $CDCl_3$): δ 168.7, 162.5 (t, $^2J_{CF}$ = 26.6 Hz), 139.2, 138.1, 137.9, 135.3, 131.4, 131.0, 130.7, 130.3, 129.7, 129.1, 128.6, 127.5, 127.4, 126.9, 105.8 (t, $^1J_{CF}$ = 248.1 Hz), 65.9, 43.8, 21.2, 17.7; ^{19}F NMR (376 MHz, $CDCl_3$): δ −123.8 (dd, J_{HF} = 35.0 Hz, J_{FF} = 302.5 Hz, 1F), −124.8 (dd, J_{HF} = 34.6 Hz, J_{FF} = 302.5 Hz, 1F); HRMS–ESI: [M + Na]$^+$ calcd for $C_{25}H_{24}F_2N_2O_2Na$, 445.1704; found, 445.1701.

N-(2-(benzylamino)-2-oxo-1-phenylethyl)-2,2-difluoro-N-(4-methoxyphenyl)acetamide (4d):
White solid. Yield: 75%. Mp: 187.0–187.8 °C, ^{1}H NMR (400 MHz, $CDCl_3$): δ 7.34–7.23 (m, 9H), 7.06–6.70 (m, 5H), 6.03 (s, 1H), 5.94 (s, 1H), 5.72 (t, J_{HF} = 53.4 Hz, 1H), 4.57–4.46 (m, 2H), 3.76 (s, 3H); ^{13}C NMR (100 MHz, $CDCl_3$): δ 168.6, 162.2 (t, $^2J_{CF}$ = 26.7 Hz), 160.0, 137.7, 136.4, 131.9, 130.8, 129.2, 129.1, 128.7, 127.6, 127.5, 124.7, 114.0, 105.9 (t, $^1J_{CF}$ = 245.9 Hz), 65.2, 55.2, 43.9; ^{19}F NMR (376 MHz, $CDCl_3$): δ −122.9 (dd, J_{HF} = 53.3 Hz, J_{FF} = 320.1 Hz, 1F), −124.1 (dd, J_{HF} = 53.7 Hz, J_{FF} = 320.1 Hz, 1F); HRMS–ESI: [M - H]$^+$ calcd for $C_{24}H_{21}F_2N_2O_3$, 423.1520; found, 423.1517.

N-(2-(benzylamino)-1-(4-methoxyphenyl)-2-oxoethyl)-2,2-difluoro-N-phenylacetamide (4e):
White solid. Yield: 70%. Mp: 193.6–194.7 °C, ^{1}H NMR (400 MHz, $CDCl_3$): δ 7.68–7.30 (m, 3H), 7.28–6.49 (m, 11H), 6.08 (s, 1H), 5.93 (t, J = 4.4 Hz, 1H), 5.75 (t, J_{HF} = 53.5 Hz, 1H), 4.57–4.46 (m, 2H), 3.76 (s, 3H); ^{13}C NMR (100 MHz, $CDCl_3$): δ 168.5, 162.6 (t, $^2J_{CF}$ = 26.5 Hz), 159.8, 137.8, 132.9, 132.1, 132.0, 130.6, 130.2, 129.0, 128.6, 127.6, 127.5, 114.1, 105.9 (t, $^1J_{CF}$ = 245.0 Hz), 65.5, 55.4, 43.8; ^{19}F NMR (376 MHz, $CDCl_3$): δ −123.1 (dd, J_{HF} = 53.5 Hz, J_{FF} = 319.5 Hz, 1F), −124.3 (dd, J_{HF} = 53.5 Hz, J_{FF} = 319.8 Hz, 1F); HRMS–ESI: [M + Na]$^+$ calcd for $C_{24}H_{22}F_2N_2O_3Na$, 447.1496; found, 447.1497.

N-(2-(benzylamino)-1-(4-methoxyphenyl)-2-oxoethyl)-2,2-difluoro-N-o-tolylacetamide (4f):
White solid. Yield: 67%. Mp: 139.5–141.0 °C, ^{1}H NMR (400 MHz, $CDCl_3$): δ 7.69–7.30 (m, 5H), 7.26–7.00 (m, 8H), 5.94 (s, 1H), 5.90 (s, 1H), 5.66 (t, J_{HF} = 53.6 Hz, 1H), 4.56–4.46 (m, 2H), 3.75 (s, 3H), 1.83 (s, 3H); ^{13}C NMR (100 MHz, $CDCl_3$): δ 168.8, 162.5 (t, $^2J_{CF}$ = 26.7 Hz), 160.1, 138.1, 137.9, 135.1, 132.2, 131.0, 129.7, 129.1, 128.7, 127.5, 127.4, 126.9, 123.8, 113.7, 105.8 (t, $^1J_{CF}$ = 246.1 Hz), 65.3, 55.2, 43.8, 17.7; ^{19}F NMR (376 MHz, $CDCl_3$): δ −123.8 (dd, J_{HF} = 31.8 Hz, J_{FF} = 299.7 Hz, 1F), −124.8 (dd, J_{HF} = 31.4 Hz, J_{FF} = 299.7 Hz, 1F); HRMS–ESI: [M + Na]$^+$ calcd for $C_{25}H_{24}F_2N_2O_3Na$, 461.1653; found, 461.1656.

N-(2-(benzylamino)-1-(4-methoxyphenyl)-2-oxoethyl)-2,2-difluoro-N-p-tolylacetamide (4g):
White solid. Yield: 78%. Mp: 169.4–170.5 °C, ^{1}H NMR (400 MHz, $CDCl_3$): δ 7.54–7.30 (m, 3H), 7.26–6.52 (m, 10H), 6.01 (s, 1H), 5.93 (s, 1H), 5.72 (t, J_{HF} = 53.5 Hz, 1H), 4.56–4.45 (m, 2H), 3.77 (s, 3H), 2.32 (s, 3H); ^{13}C NMR (100 MHz, $CDCl_3$): δ 168.8, 162.3 (t, $^2J_{CF}$ = 26.5 Hz), 159.9, 139.2, 137.9, 133.7, 131.9, 130.5, 129.7, 128.6, 127.6, 127.4, 124.9, 113.9, 105.8 (t, $^1J_{CF}$ = 245.5 Hz), 65.0, 55.2, 43.8, 21.1; ^{19}F NMR (376 MHz, $CDCl_3$): δ −123.1 (dd, J_{HF} = 53.5 Hz, J_{FF} = 319.9 Hz, 1F), −124.2 (dd, J_{HF} = 53.4 Hz, J_{FF} = 320.0 Hz, 1F); HRMS–ESI: [M + Na]$^+$ calcd for $C_{25}H_{24}F_2N_2O_3Na$, 461.1653; found, 461.1654.

***N*-(2-(benzylamino)-1-(4-methoxyphenyl)-2-oxoethyl)-2,2-difluoro-*N*-(4-fluorophenyl)acetamide (4h):**

White solid. Yield: 71%. Mp: 155.3–156.1 °C, ^{1}H NMR (400 MHz, $CDCl_3$): δ 7.82–7.31 (m, 4H), 7.27–6.54 (m, 9H), 6.06 (s, 1H), 5.88 (s, 1H), 5.74 (t, J_{HF} = 53.4 Hz, 1H), 4.57–4.45 (m, 2H), 3.77 (s, 3H); ^{13}C NMR (100 MHz, $CDCl_3$): δ 168.7, 162.5 (d, $^{1'}J_{CF}$ = 250.4 Hz), 162.2 (t, $^{2}J_{CF}$ = 26.6 Hz), 160.1, 137.7, 133.0, 132.1 (d, $^{3'}J_{CF}$ = 3.1 Hz), 131.9, 128.7, 127.6, 127.5, 124.4, 116.0 (d, $^{2'}J_{CF}$ = 24.2 Hz), 114.1, 106.1 (t, $^{1}J_{CF}$ = 246.7 Hz), 64.7, 55.2, 43.9; ^{19}F NMR (376 MHz, $CDCl_3$): δ (−111.0)–(−111.1) (m, 1F), −122.6 (dd, J_{HF} = 53.3 Hz, J_{FF} = 319.9 Hz, 1F), −124.1 (dd, J_{HF} = 53.5 Hz, J_{FF} = 319.7 Hz, 1F); HRMS–ESI: $[M + Na]^+$ calcd for $C_{24}H_{21}F_3N_2O_3Na$, 465.1402; found, 465.1402.

***N*-(2-(benzylamino)-1-(4-fluorophenyl)-2-oxoethyl)-2,2-difluoro-*N*-phenylacetamide (4i):**

White solid. Yield: 75%. Mp: 177.2–177.9 °C, ^{1}H NMR (400 MHz, $CDCl_3$): δ 7.34–7.28 (m, 6H), 7.27–6.63 (m, 8H), 6.04 (s, 1H), 6.00 (t, J = 5.0 Hz, 1H), 5.71 (t, J_{HF} = 53.4 Hz, 1H), 4.57–4.45 (m, 2H); ^{13}C NMR (100 MHz, $CDCl_3$): δ 168.3, 163.0 (d, $^{1'}J_{CF}$ = 249.5 Hz), 162.3 (t, $^{2}J_{CF}$ = 26.7 Hz), 137.7, 136.0, 132.5 (d, $^{3'}J_{CF}$ = 8.4 Hz), 130.8, 129.4, 129.2, 128.7, 128.6, 127.6, 127.5, 115.6 (d, $^{2'}J_{CF}$ = 21.6 Hz), 105.8 (t, $^{1}J_{CF}$ = 246.4 Hz), 64.6, 43.9; ^{19}F NMR (376 MHz, $CDCl_3$): δ (−111.5)–(−111.6) (m, 1F), −122.9 (dd, J_{HF} = 53.3 Hz, J_{FF} = 320.6 Hz, 1F), −124.2 (dd, J_{HF} = 53.5 Hz, J_{FF} = 320.7 Hz, 1F); HRMS–ESI: $[M + Na]^+$ calcd for $C_{23}H_{19}F_3N_2O_2Na$, 435.1296; found, 435.1297.

***N*-(2-(benzylamino)-1-(4-fluorophenyl)-2-oxoethyl)-2,2-difluoro-*N*-*o*-tolylacetamide (4j):**

White solid. Yield: 69%. Mp: 181.9–182.5 °C, ^{1}H NMR (400 MHz, $CDCl_3$): δ 7.69–7.30 (m, 3H), 7.26–6.83 (m, 10H), 6.10 (s, 1H), 5.99 (s, 1H), 5.66 (t, J_{HF} = 53.5 Hz, 1H), 4.55–4.43 (m, 2H), 1.81 (s, 3H); ^{13}C NMR (100 MHz, $CDCl_3$): δ 168.5, 163.0 (d, $^{1'}J_{CF}$ = 249.9 Hz), 162.6 (t, $^{2}J_{CF}$ = 26.8 Hz), 138.0, 137.8, 134.7, 132.8 (d, $^{3'}J_{CF}$ = 8.3 Hz), 131.5, 131.0, 129.9, 129.6, 128.7, 127.6, 127.5, 127.1, 115.3 (d, $^{2'}J_{CF}$ = 21.6 Hz), 105.8 (t, $^{1}J_{CF}$ = 247.2 Hz), 65.9, 43.8, 17.7; ^{19}F NMR (376 MHz, $CDCl_3$): δ (−111.3)–(−111.4) (m, 1F), −123.9 (dd, J_{HF} = 30.5 Hz, J_{FF} = 298.7 Hz, 1F), −124.8 (dd, J_{HF} = 30.1 Hz, J_{FF} = 298.6 Hz, 1F); HR-MS(ESI): $[M + Na]^+$ calcd for $C_{24}H_{21}F_3N_2O_2Na$, 449.1453; found, 449.1453.

***N*-(2-(benzylamino)-1-(4-fluorophenyl)-2-oxoethyl)-2,2-difluoro-*N*-(4-methoxyphenyl)acetamide (4k):**

White solid. Yield: 74%. Mp: 186.2–187.4 °C, ^{1}H NMR (400 MHz, $CDCl_3$): δ 7.64–7.29 (m, 4H), 7.25–6.46 (m, 9H), 6.07 (s, 1H), 5.99 (t, J = 4.9 Hz, 1H), 5.74 (t, J_{HF} = 53.5 Hz, 1H), 4.57–4.46 (m, 2H), 3.79 (s, 3H); ^{13}C NMR (100 MHz, $CDCl_3$): δ 168.6, 162.9 (d, $^{1'}J_{CF}$ = 249.3 Hz), 162.6 (t, $^{2}J_{CF}$ = 26.7 Hz), 159.9, 137.8, 132.5 (d, $^{3'}J_{CF}$ = 8.3 Hz), 132.1, 128.8 (d, $^{4'}J_{CF}$ = 3.3 Hz), 128.7, 128.4, 128.2, 127.5, 127.4, 115.6 (d, $^{2'}J_{CF}$ = 21.6 Hz), 114.2, 105.8 (t, $^{1}J_{CF}$ = 245.5 Hz), 64.3, 55.4, 43.8; ^{19}F NMR (376 MHz, $CDCl_3$): δ (−111.5)–(−111.6) (m, 1F), −123.1 (dd, J_{HF} = 53.3 Hz, J_{FF} = 320.1 Hz, 1F), −124.4 (dd, J_{HF} = 53.4 Hz, J_{FF} = 320.0 Hz, 1F); HRMS–ESI: $[M + Na]^+$ calcd for $C_{24}H_{21}F_3N_2O_3Na$, 465.1402; found, 465.1400.

2,2-Difluoro-*N*-(2-oxo-1-phenyl-2-(phenylamino)ethyl)-*N*-phenylacetamide (4l):
White solid. Yield: 74%. Mp: 186.2–187.4 °C, ^{1}H NMR (400 MHz, $CDCl_3$): δ 7.51–7.29 (m, 9H), 7.27–7.10 (m, 6H), 6.34 (s, 1H), 6.26 (s, 1H), 5.76 (t, J_{HF} = 53.4 Hz, 1H); ^{13}C NMR (100 MHz, $CDCl_3$): δ 166.8, 162.5 (t, $^{2}J_{CF}$ = 26.6 Hz), 137.4, 136.1, 132.3, 130.9, 130.6, 129.3, 129.2, 129.1, 128.9, 128.8, 124.6, 120.1, 105.9 (t, $^{1}J_{CF}$ = 246.0 Hz), 66.2; ^{19}F NMR (376 MHz, $CDCl_3$): δ −122.9 (dd, J_{HF} = 53.3 Hz, J_{FF} = 320.4 Hz, 1F), −124.1 (dd, J_{HF} = 53.5 Hz, J_{FF} = 320.6 Hz, 1F); HRMS–ESI: $[M + Na]^+$ calcd for $C_{22}H_{18}F_2N_2O_2Na$, 403.1234; found, 403.1233.

2,2-Difluoro-*N*-(1-(4-methoxyphenyl)-2-oxo-2-(phenylamino)ethyl)-*N*-phenylacetamide (4m):
White solid. Yield: 74%. Mp: 186.2–187.4 °C, ^{1}H NMR (400 MHz, $CDCl_3$): δ 7.81–7.29 (m, 9H), 7.14–6.76 (m, 5H), 6.61 (s, 1H), 6.22 (s, 1H), 5.75 (t, J_{HF} = 53.4 Hz, 1H), 3.78 (s, 3H); ^{13}C NMR (100 MHz, $CDCl_3$): δ 166.9, 162.3 (t, $^{2}J_{CF}$ = 25.6 Hz), 160.2, 137.3, 136.1, 132.0, 130.9, 129.3, 129.0, 124.7, 124.2, 120.1, 114.1, 105.9 (t, $^{1}J_{CF}$ = 245.6 Hz), 100.5, 55.3, 18.4; ^{19}F NMR (376 MHz, $CDCl_3$): δ −122.9 (dd, J_{HF} = 53.4 Hz, J_{FF} = 320.4 Hz, 1F), −124.1 (dd, J_{HF} = 53.4 Hz, J_{FF} = 320.4 Hz, 1F); HRMS–ESI: $[M + Na]^+$ calcd for $C_{23}H_{20}F_2N_2O_3Na$, 433.1340; found, 433.1339.

Preparation of *N*-benzyl-2-phenyl-2-(N-phenylacetamido)acetamide (**5**)

To a stirred aniline (0.093 g, 1 mmol), benzaldehyde (0.106 g, 1 mmol) was added in portions for about 5 min. The mixture was stirred for 30 min at rt. Then, the reaction mixture was heated to 60 °C and (isocyanomethyl)benzene (0.117 g, 1 mmol) and acetic acid (0.060 g, 1 mmol) were added. Stirring was continued at 60 °C. After completion of the reaction (monitored by TLC), the crude residue was diluted with CH_2Cl_2 and purified by chromatography to give the desired product **5**. White solid, yield: 75%. Mp: 152.5–153.4 °C, ^{1}H NMR (400 MHz, $CDCl_3$): δ 7.33–7.14 (m, 15H), 6.15 (s, 1H), 6.09 (s, 1H), 4.55–4.47 (m, 2H), 1.89 (s, 1H); ^{13}C NMR (100 MHz, $CDCl_3$): δ 171.3, 169.9, 140.7, 138.2, 134.6, 130.4, 130.3, 128.9, 128.6, 128.4, 128.3, 128.0, 127.5, 127.3, 65.1, 43.7, 23.3.

Preparation of *N*-(2-(benzylamino)-2-oxo-1-phenylethyl)-2,2,2-trifluoro-*N*-phenylacetamide (**6**)

To a stirred aniline (0.093 g, 1 mmol), benzaldehyde (0.106 g, 1 mmol) was added in portions for about 5 min. The mixture was stirred for 30 min at rt. Then, the reaction mixture was heated to 60 °C and (isocyanomethyl)benzene (0.117 g, 1 mmol) and 2,2,2-trifluoroacetic acid (0.114 g, 1 mmol) were added. Stirring was continued at 60 °C. After completion of the reaction (monitored by TLC), the crude residue was diluted with CH_2Cl_2 and purified by chromatography to give the desired product **6**. White solid, yield: 80%. Mp: 177.8–179.3 °C, ^{1}H NMR (400 MHz, $CDCl_3$): δ 7.74–7.19 (m, 11H), 7.13–6.65 (m, 4 H), 5.97 (s, 1H), 5.84 (s, 1H), 4.58–4.47 (m, 2H); ^{13}C NMR (100 MHz, $CDCl_3$): δ 168.1, 157.5 (q, $^{2}J_{CF}$ = 36.1 Hz), 137.7, 136.4, 132.5, 130.9, 130.7, 129.2, 129.0, 128.7, 128.4, 128.2, 127.5, 127.4, 116.3 (q, $^{1}J_{CF}$ = 288.1 Hz), 67.3, 43.9; ^{19}F NMR (376 MHz, $CDCl_3$): δ −67.3 (s, 3F).

References

1. Wu, J.; Cao, S.; Liu, N.; Shen, L.; Yu, J.; Zhang, J.; Li, H.; Qian, X. *Org. Biomol. Chem.* **2010**, *8*, 2386–2391. doi:10.1039/c000835d

NMR spectra and MS spectra for all products

^{1}H NMR of **3a**

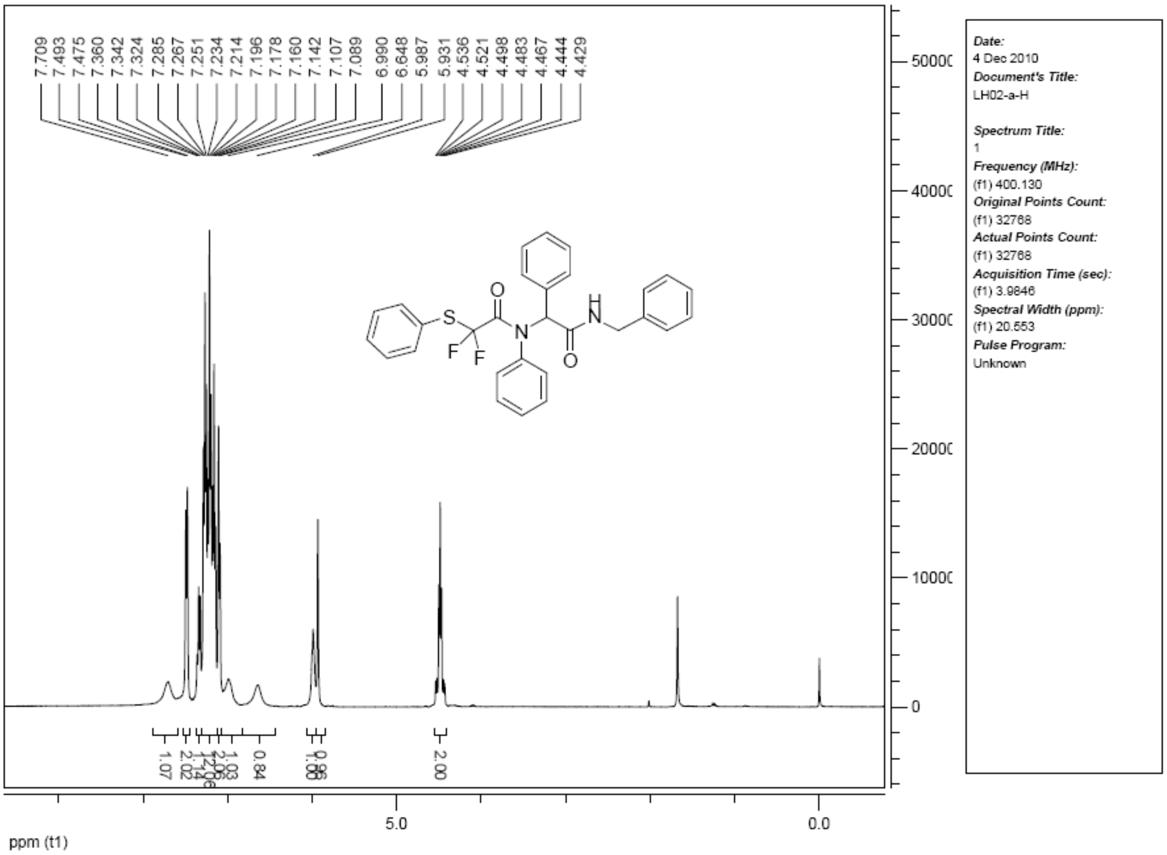

^{13}C NMR of **3a**

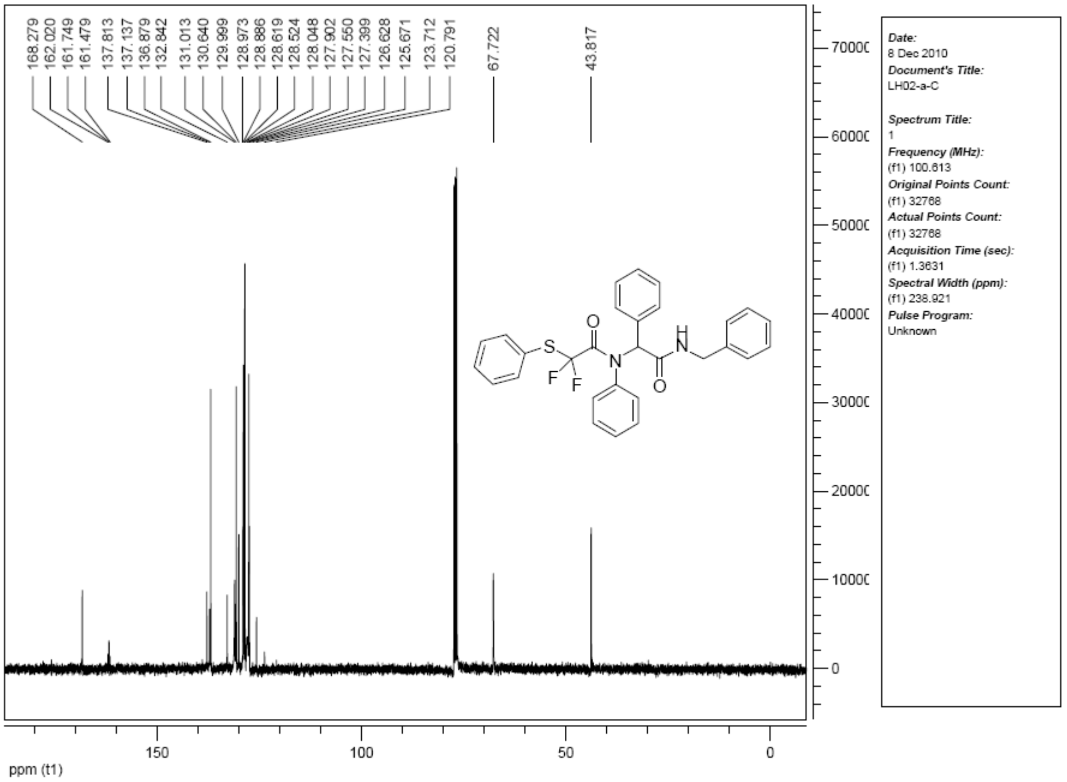

^{19}F NMR of **3a**

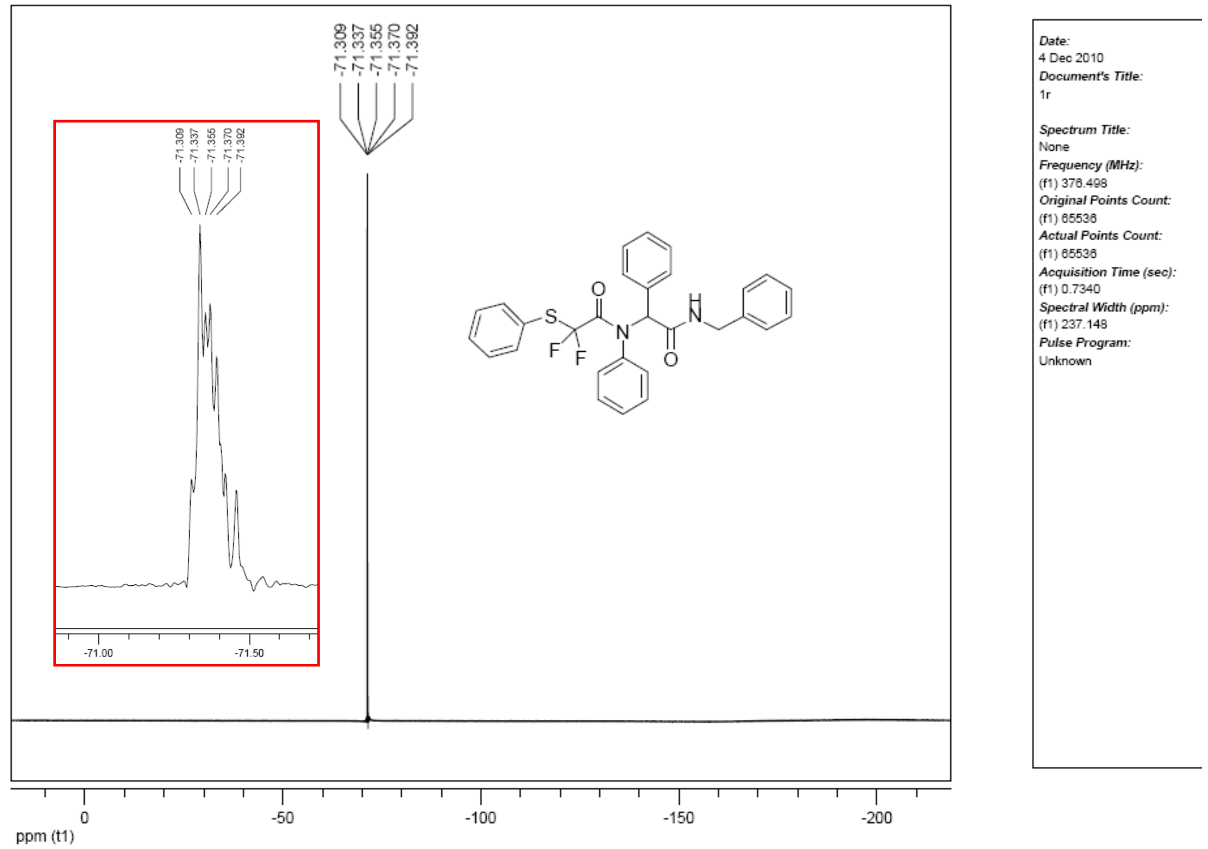

^{1}H NMR of **3b**

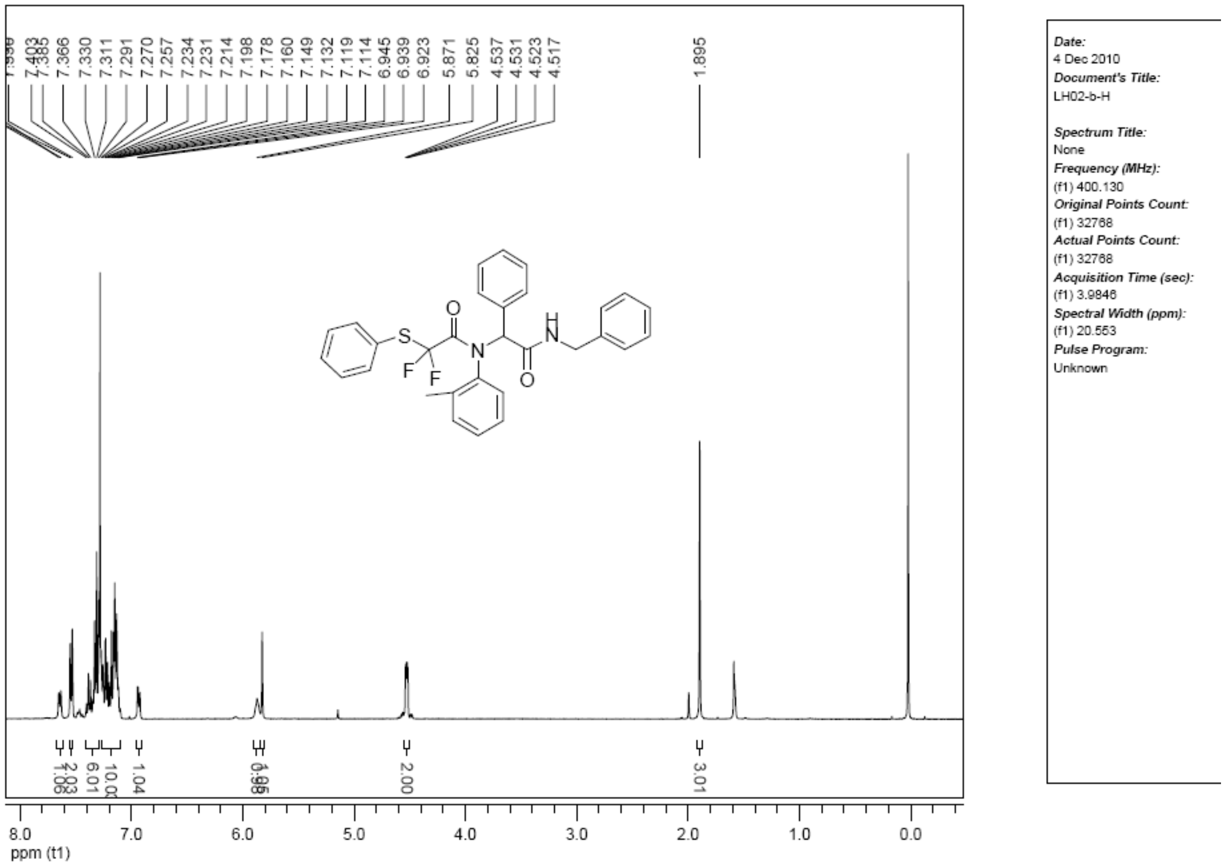

^{13}C NMR of **3b**

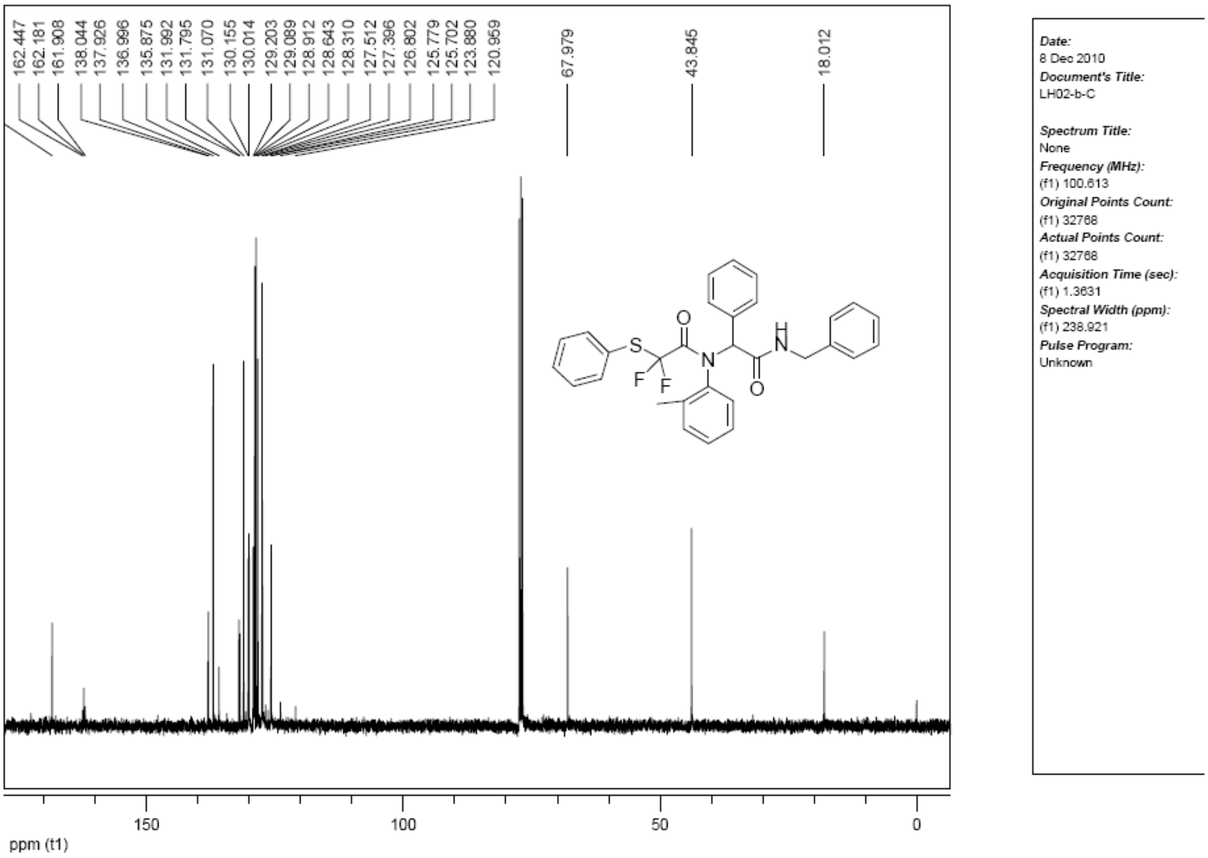

^{19}F NMR of **3b**

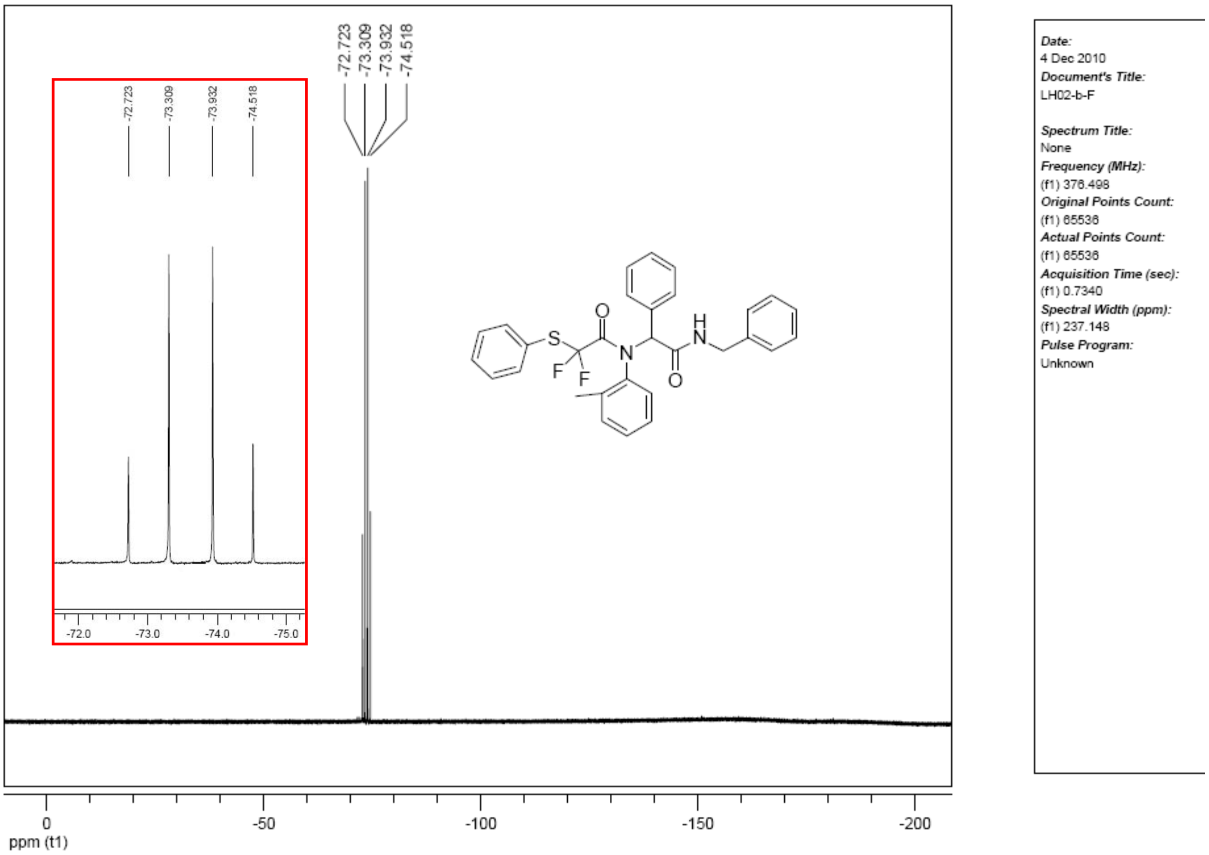

^{1}H NMR of **3c**

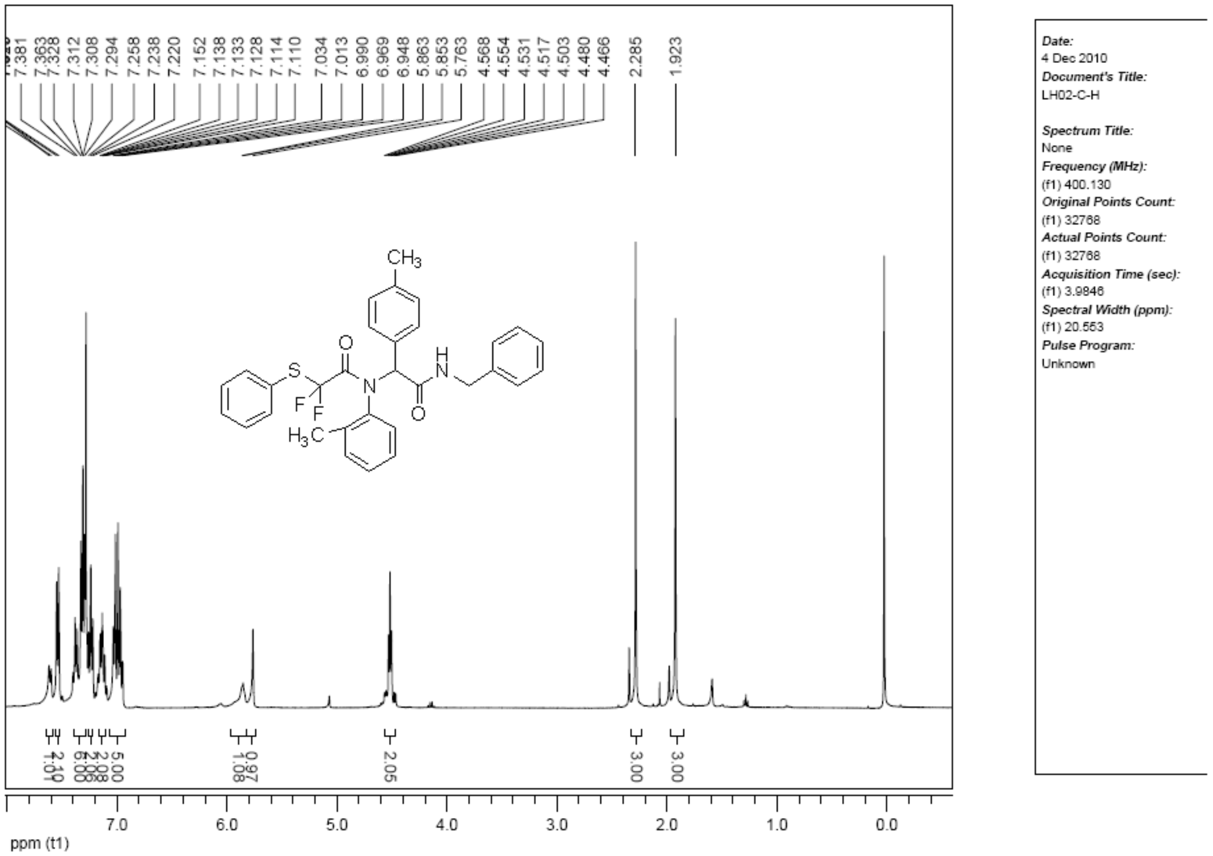

^{13}C NMR of **3c**

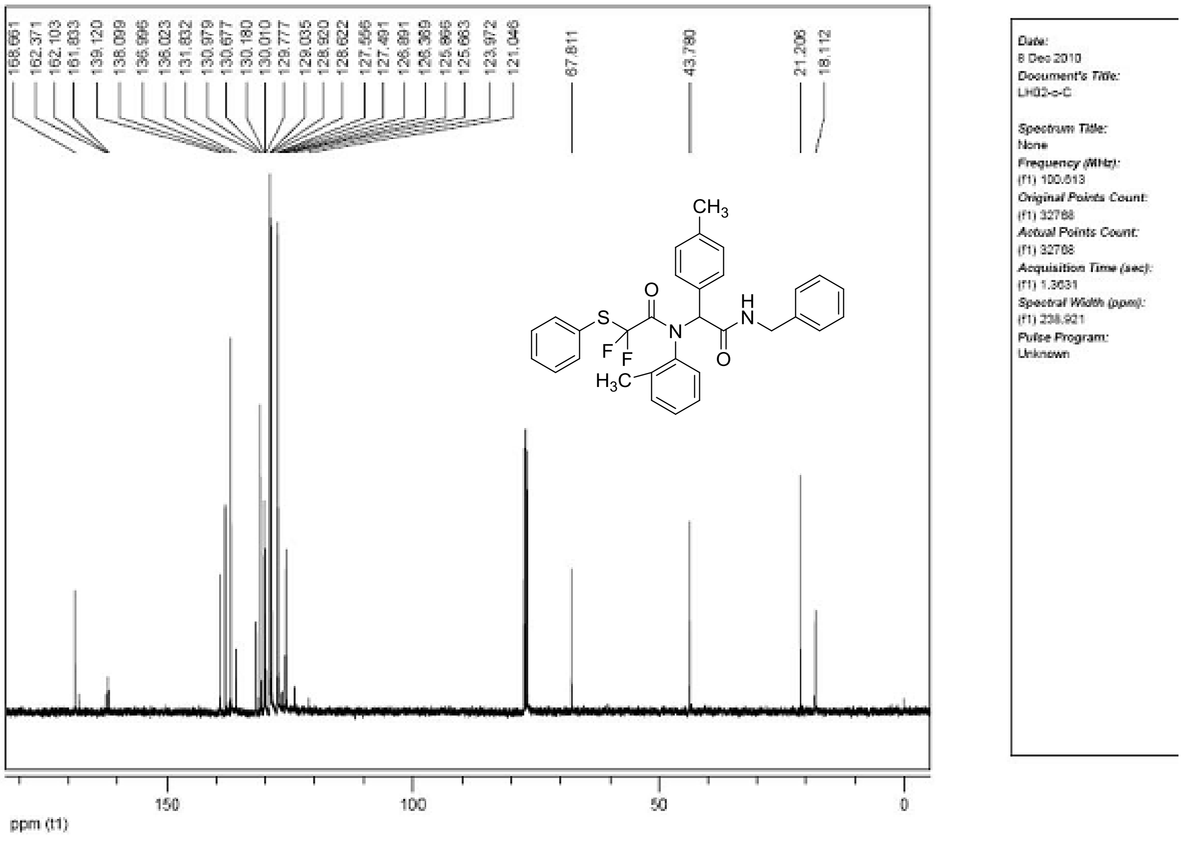

^{19}F NMR of **3c**

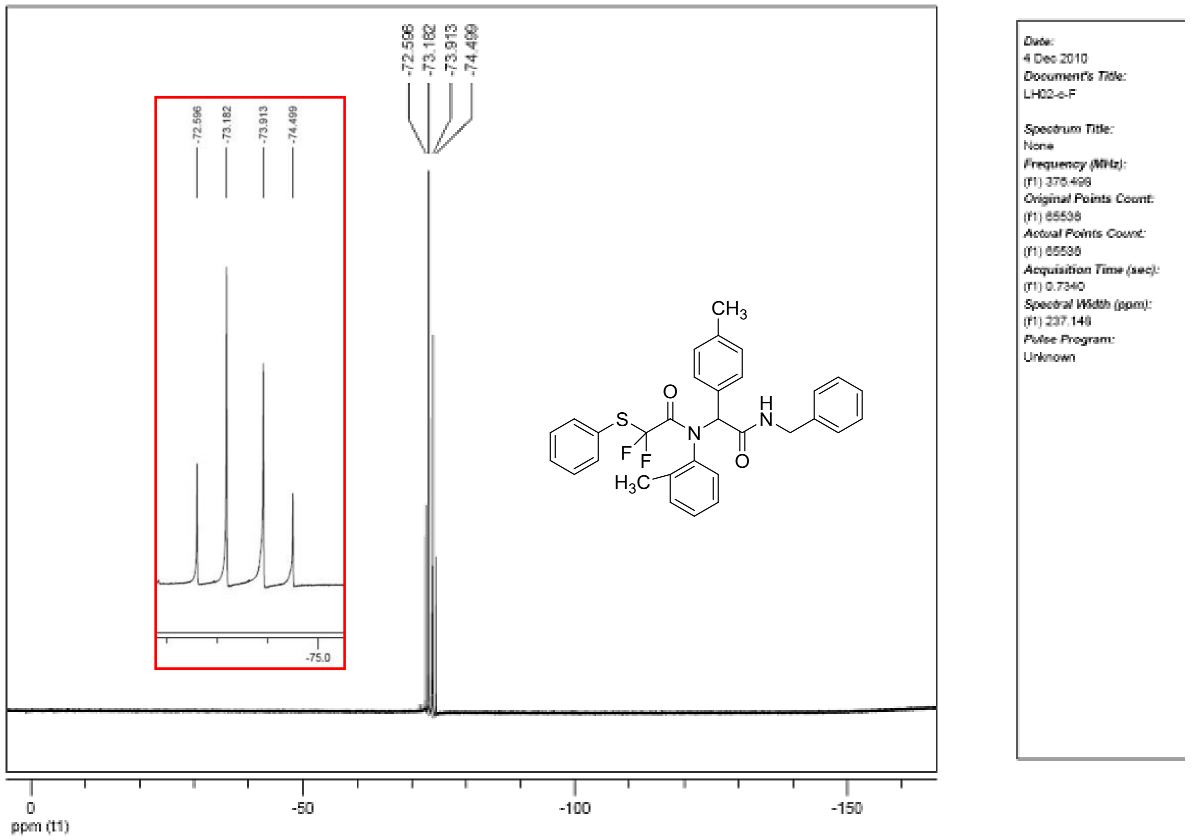

^{1}H NMR of **3d**

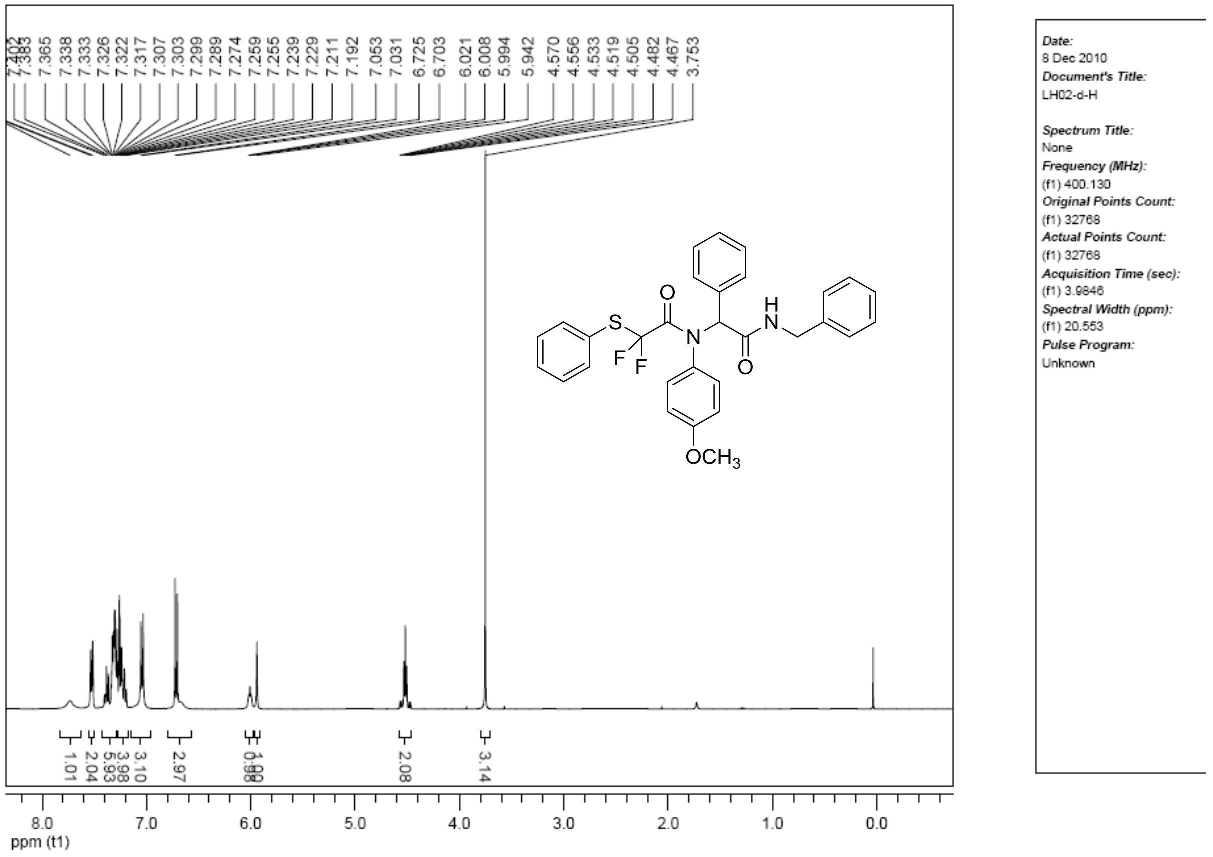

^{13}C NMR of **3d**

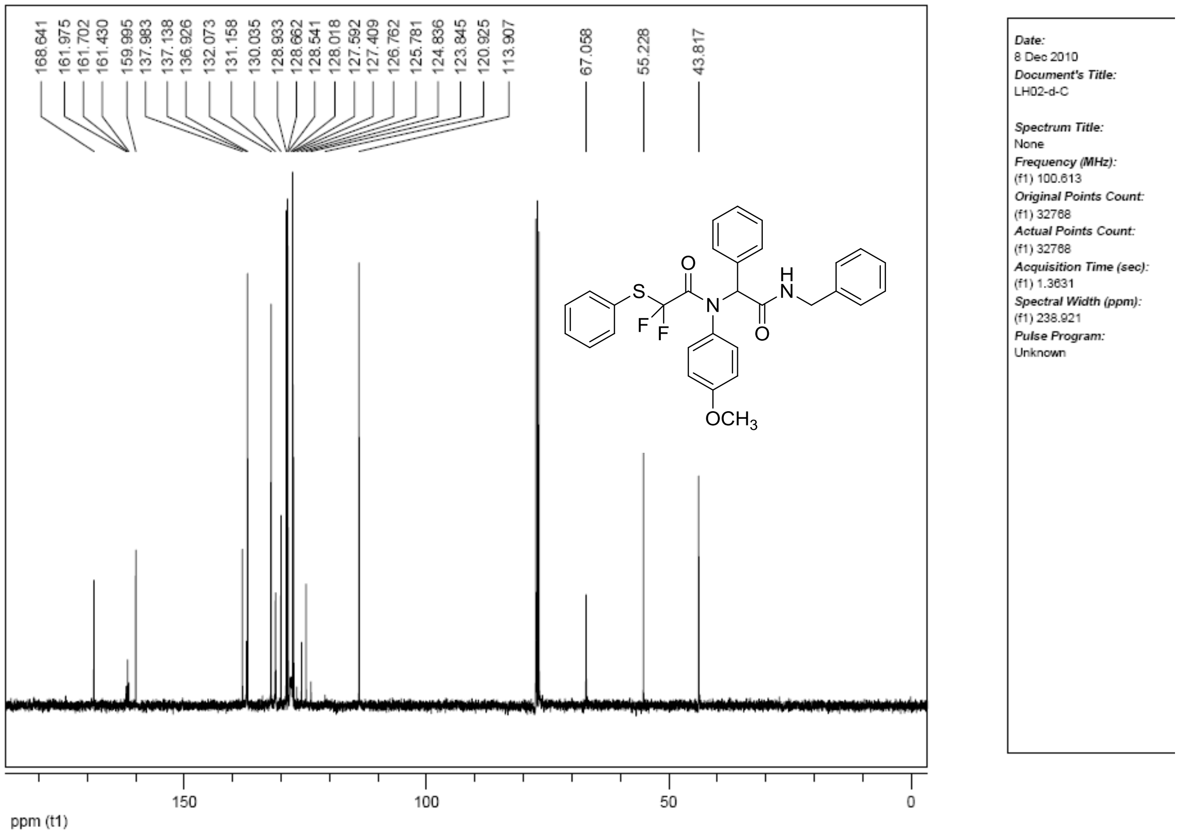

^{19}F NMR of **3d**

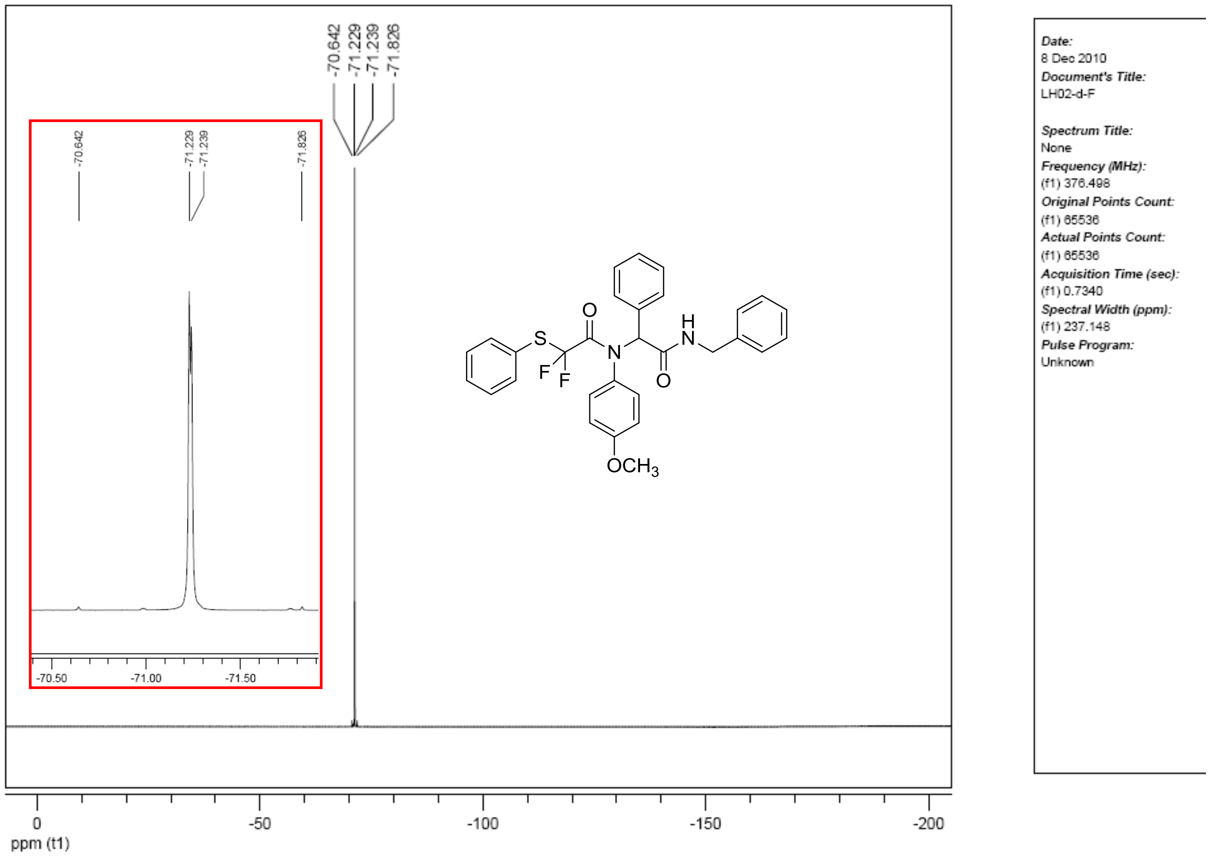

^{1}H NMR of **3e**

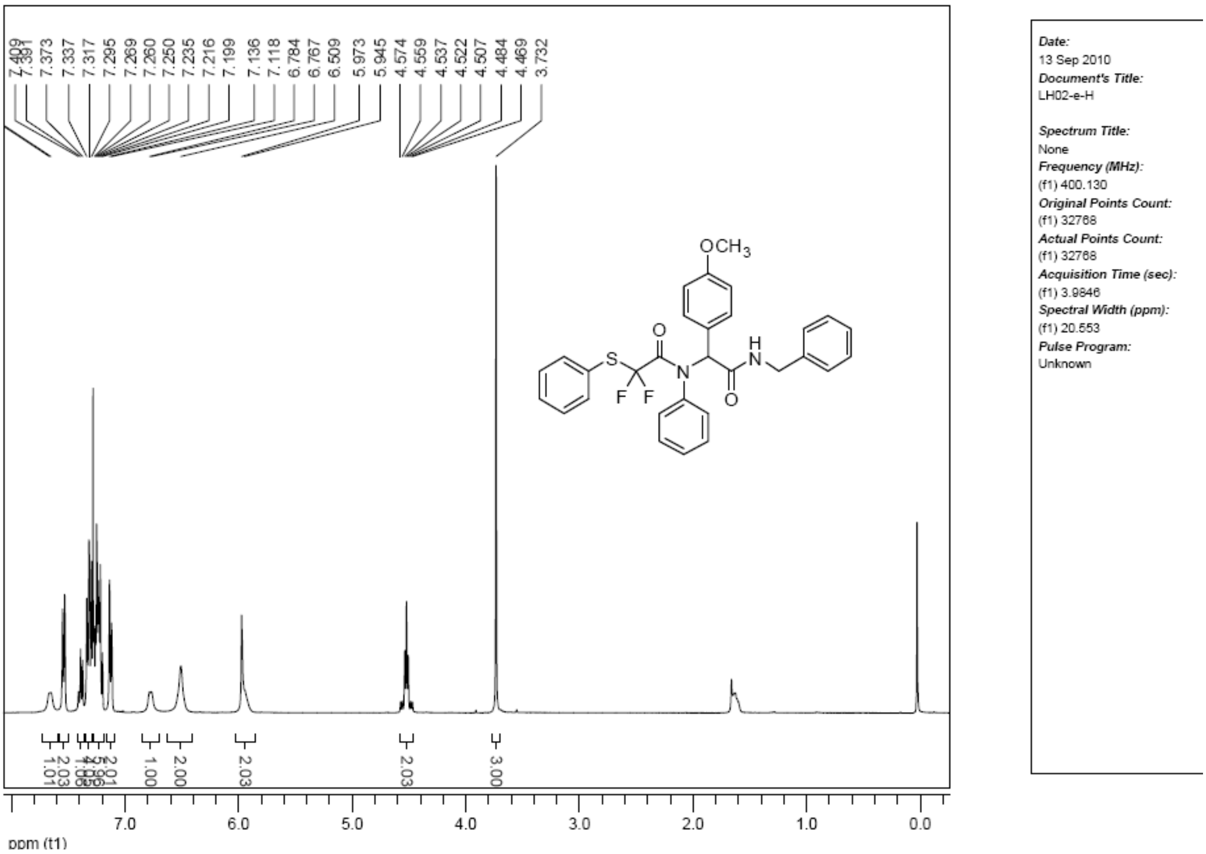

^{13}C NMR of **3e**

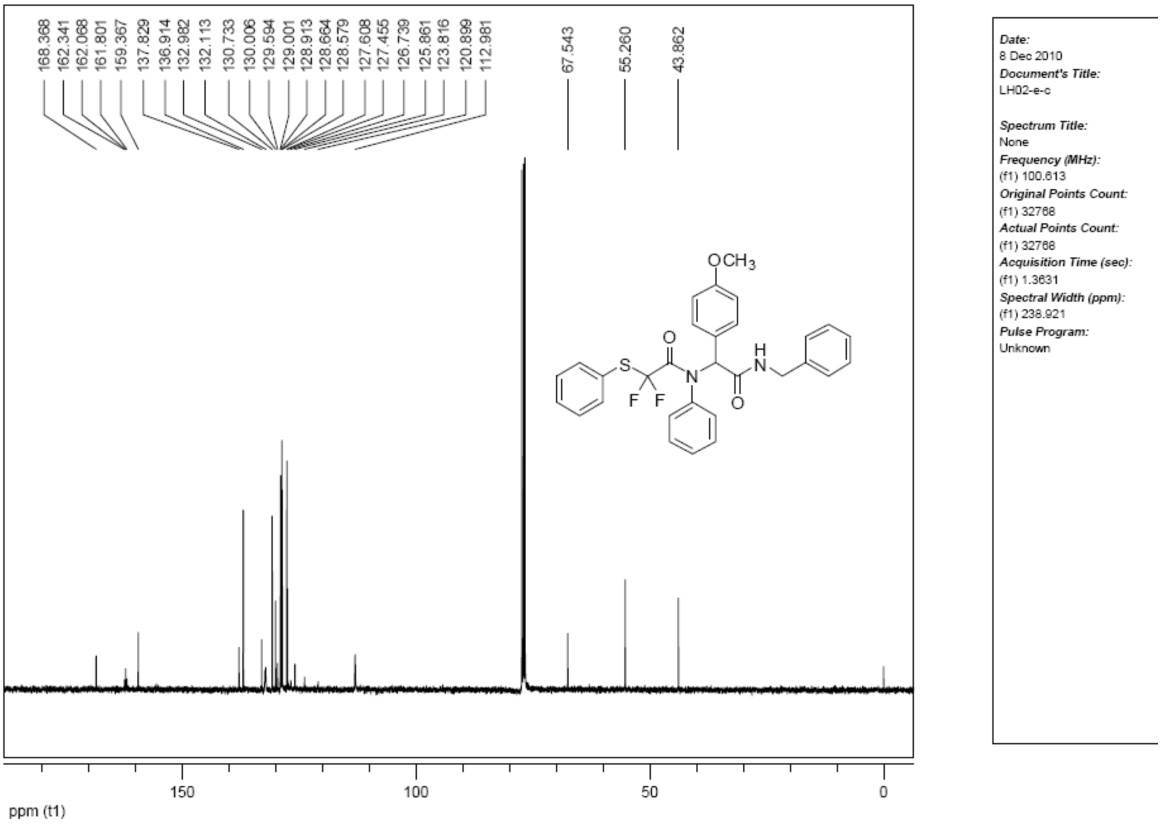

^{19}F NMR of **3e**

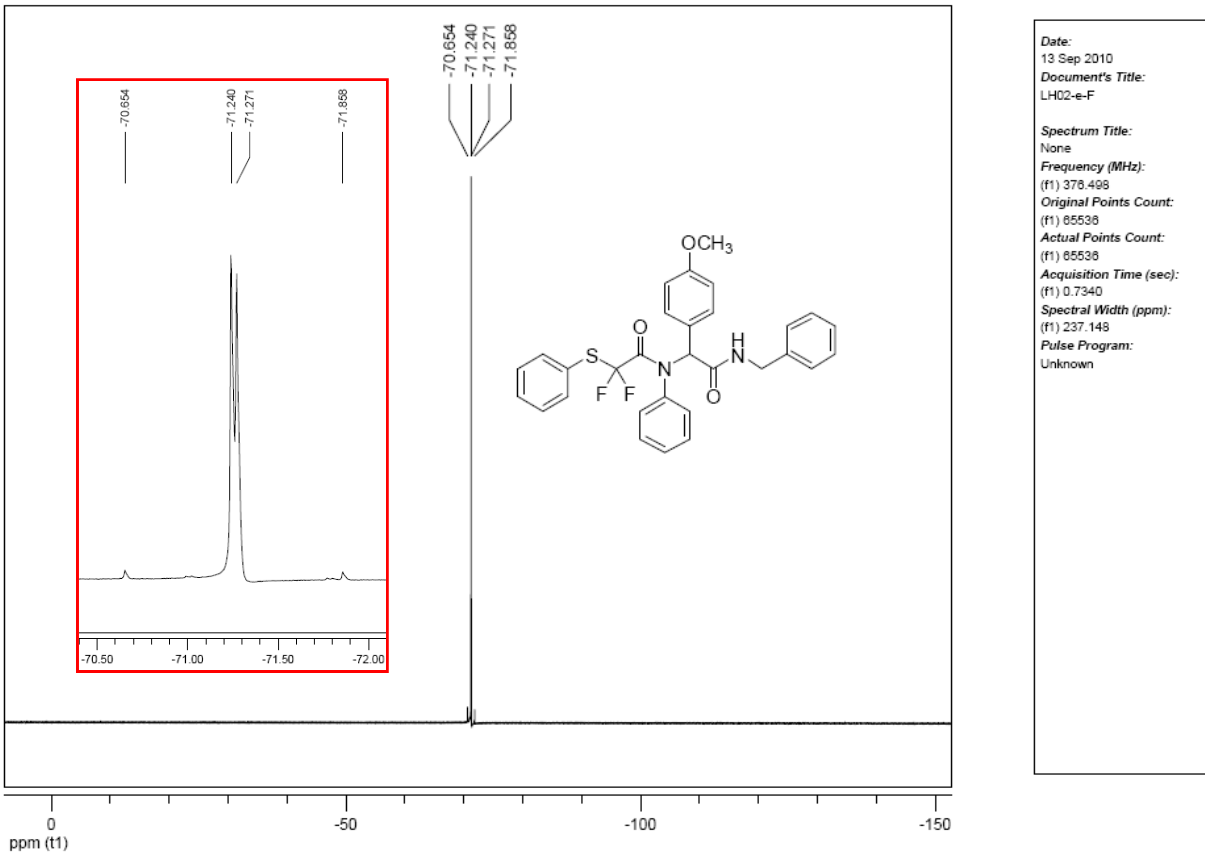

^{1}H NMR of **3f**

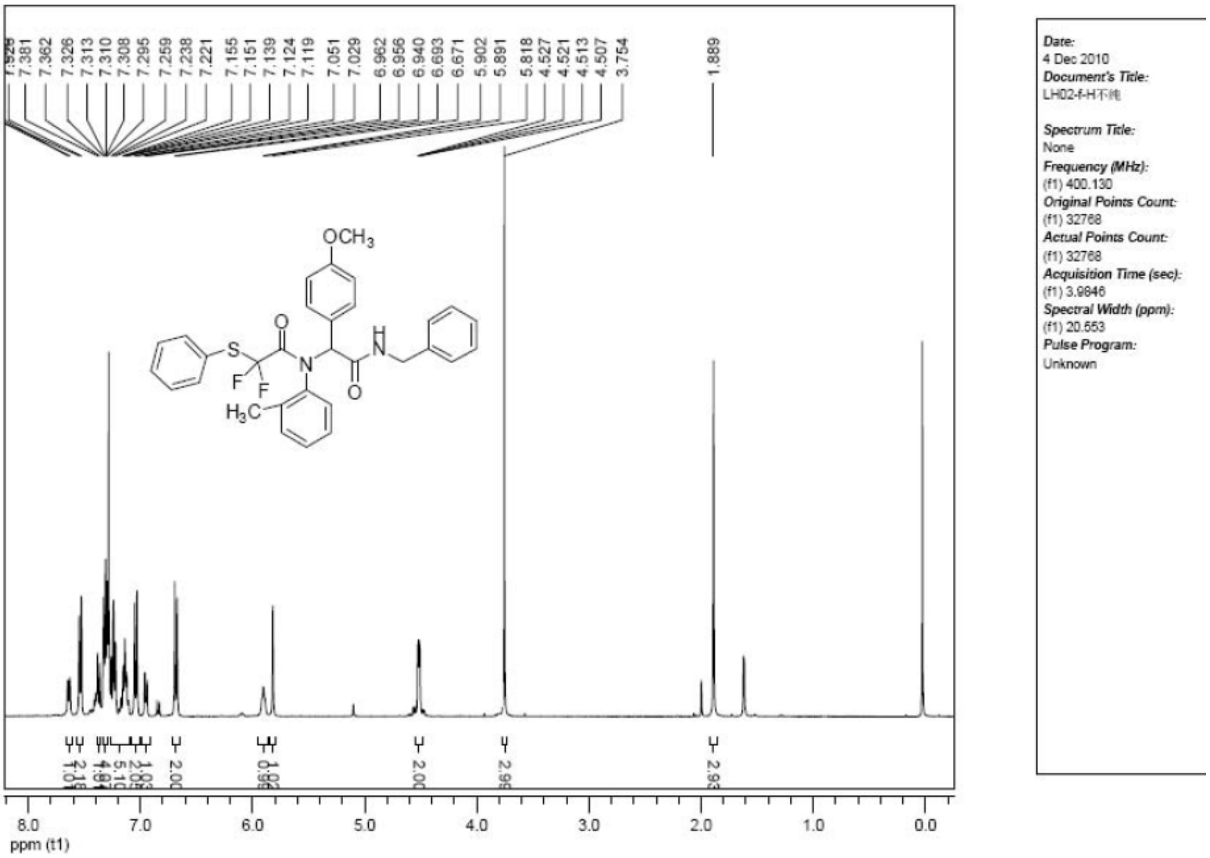

^{13}C NMR of **3f**

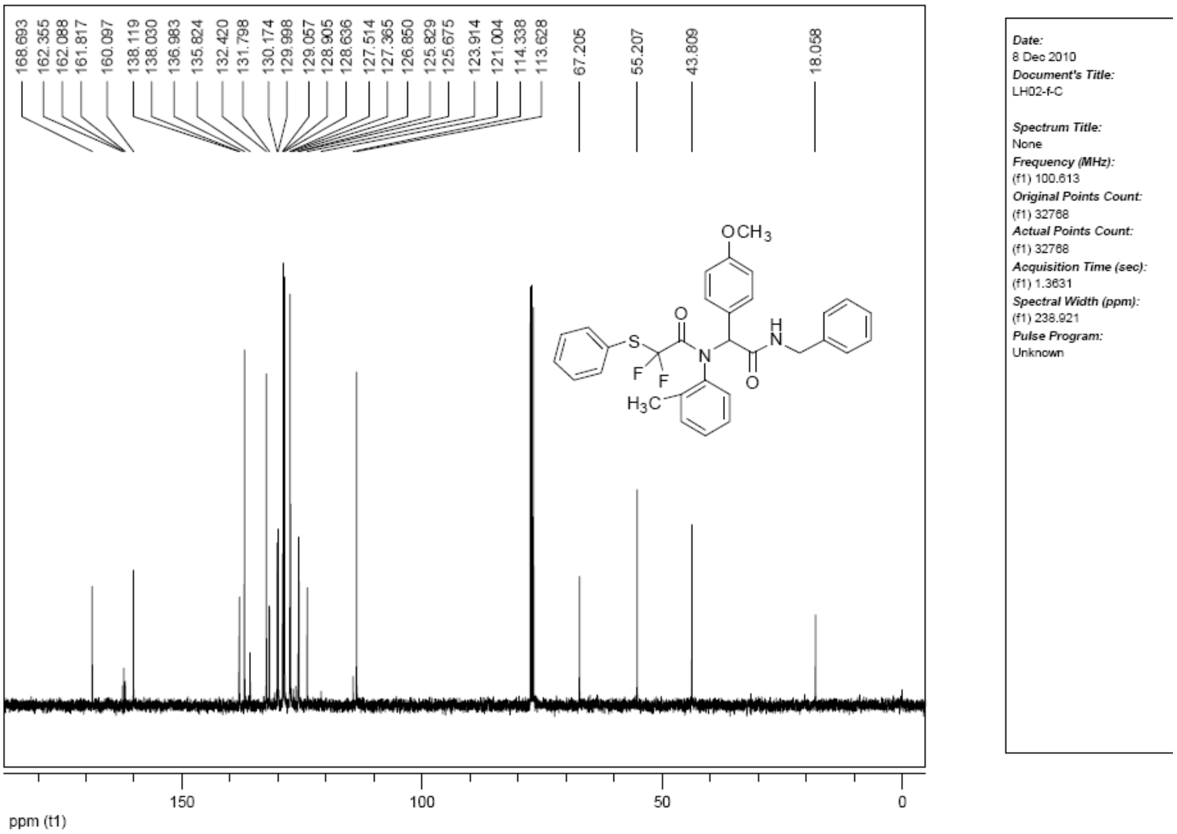

^{19}F NMR of **3f**

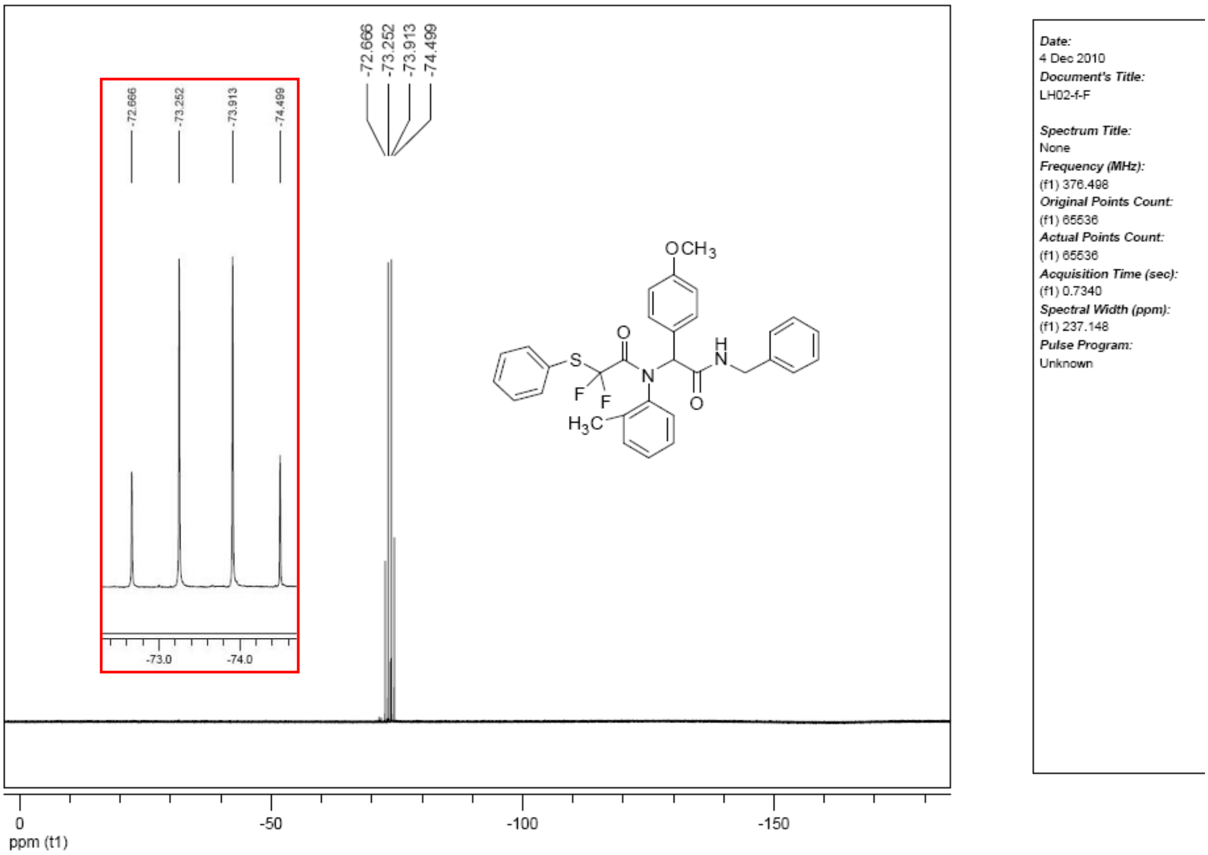

^{1}H NMR of **3g**

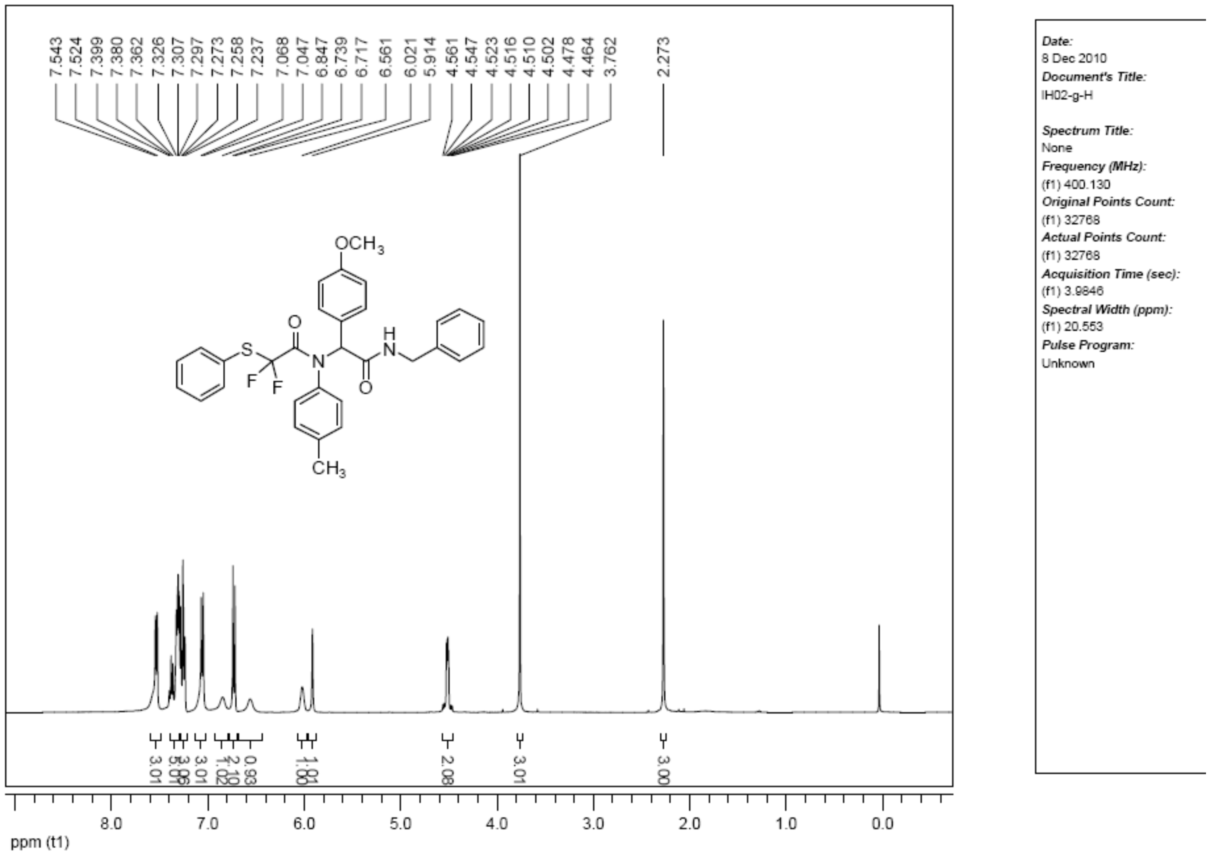

^{13}C NMR of **3g**

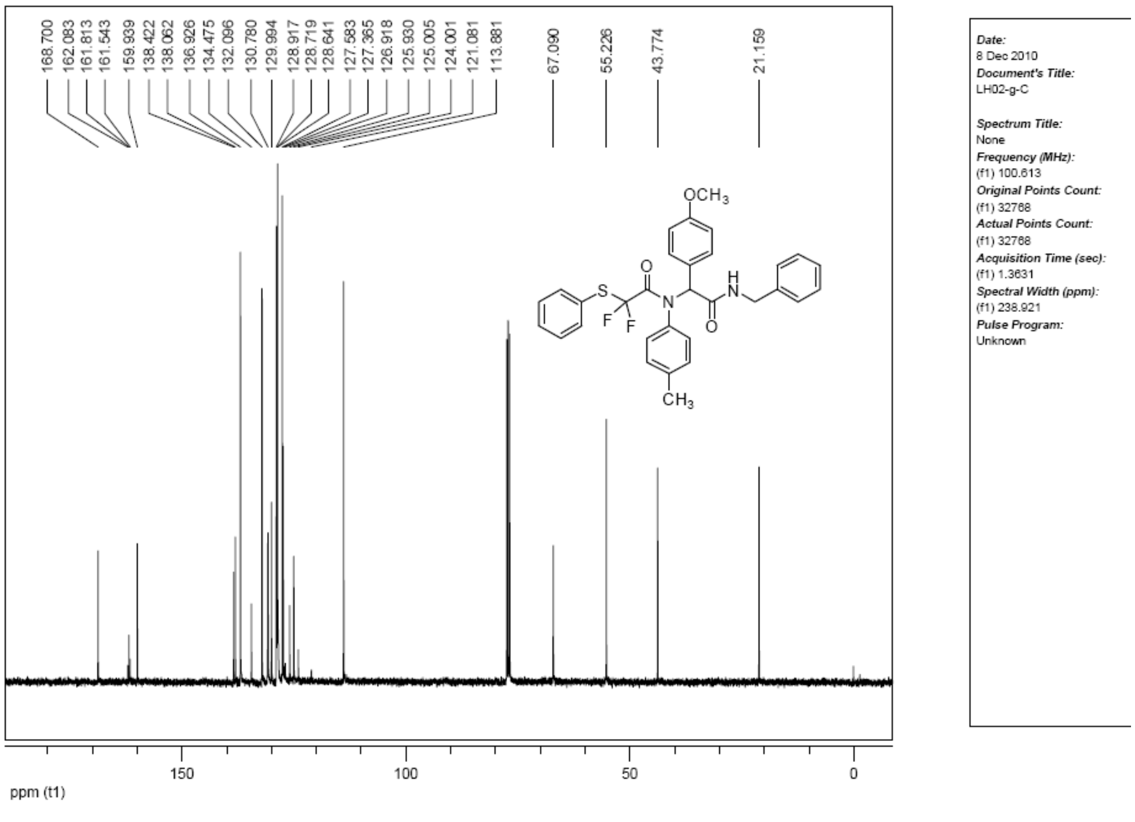

^{19}F NMR of **3g**

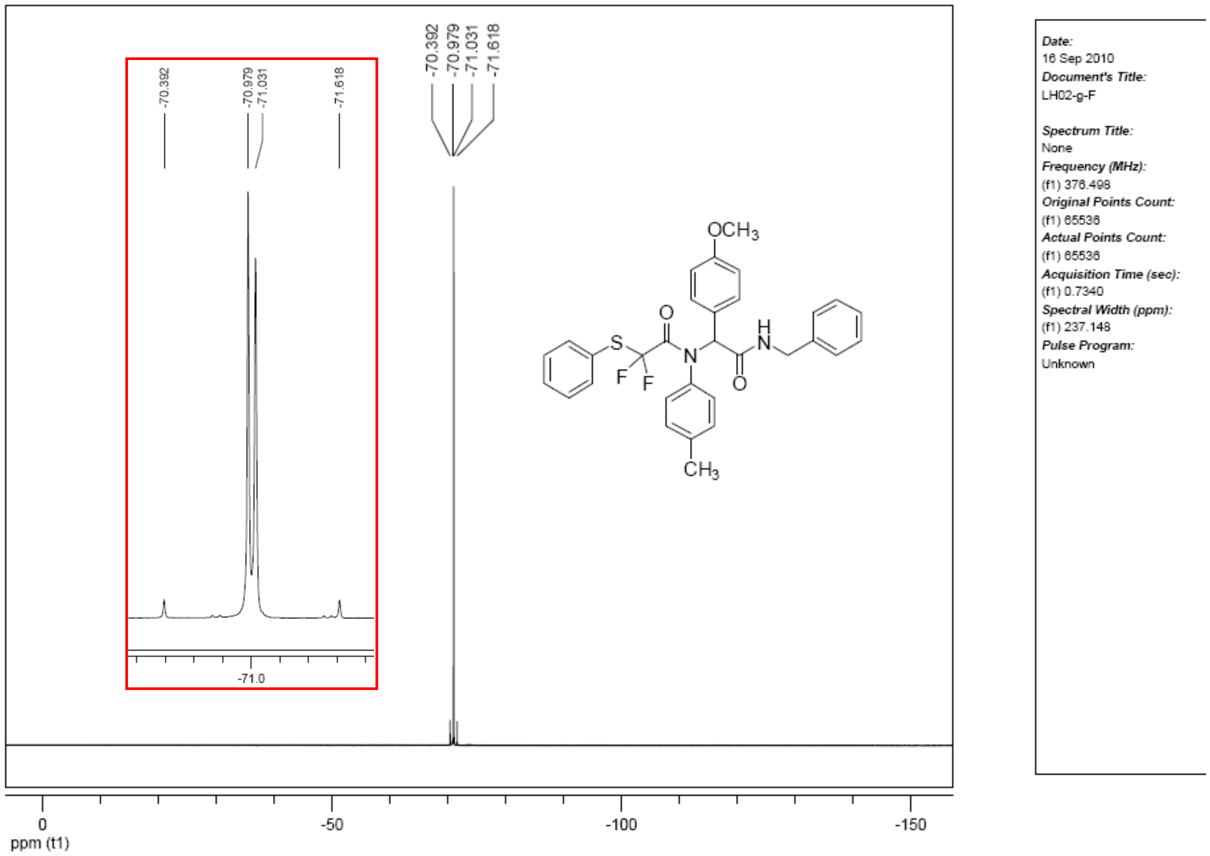

^{1}H NMR of **3h**

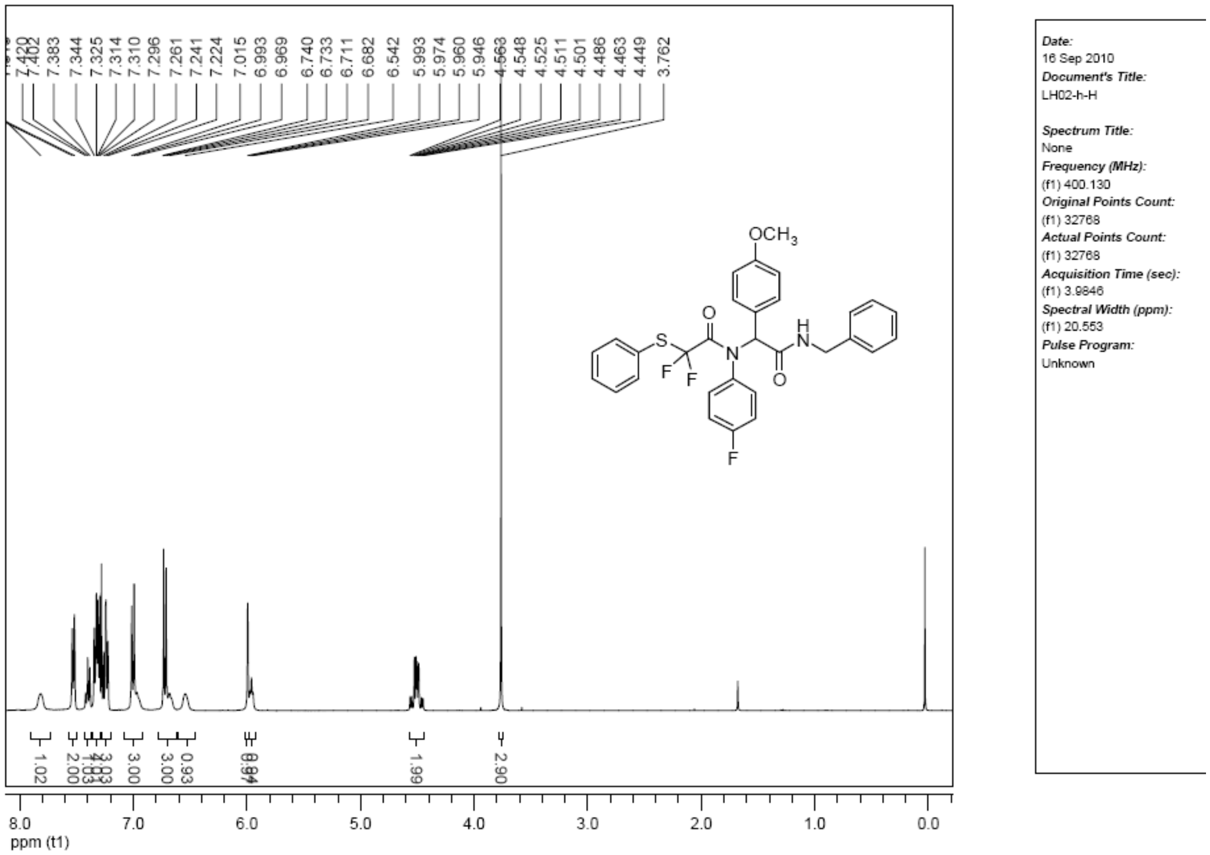

^{13}C NMR of **3h**

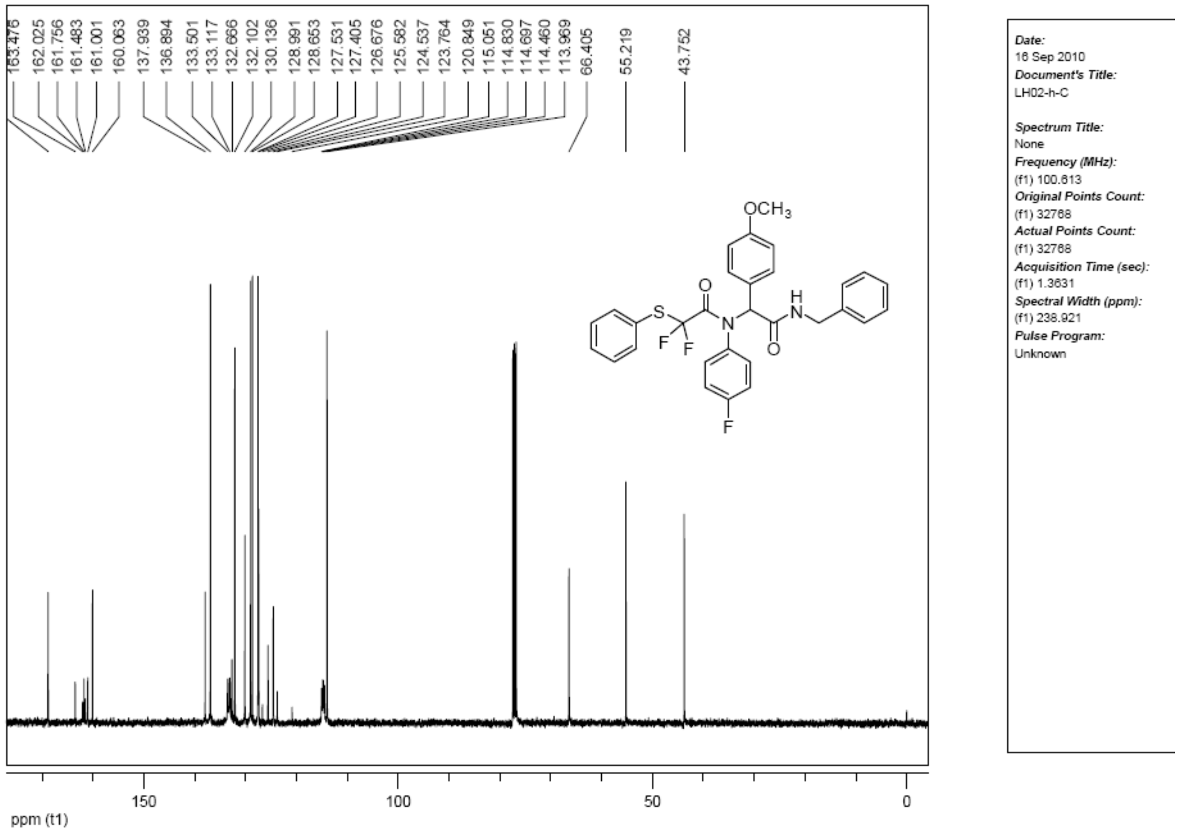

^{19}F NMR of **3h**

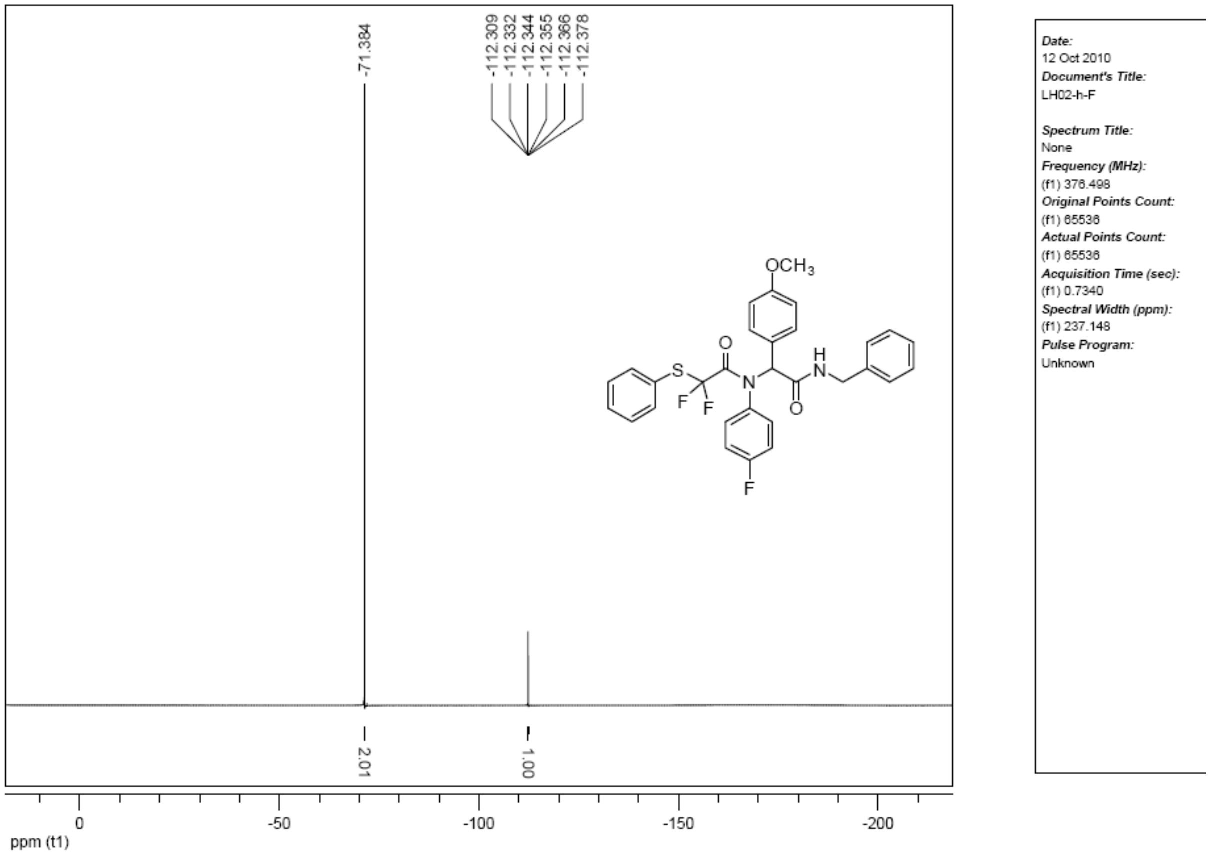

^{1}H NMR of **3i**

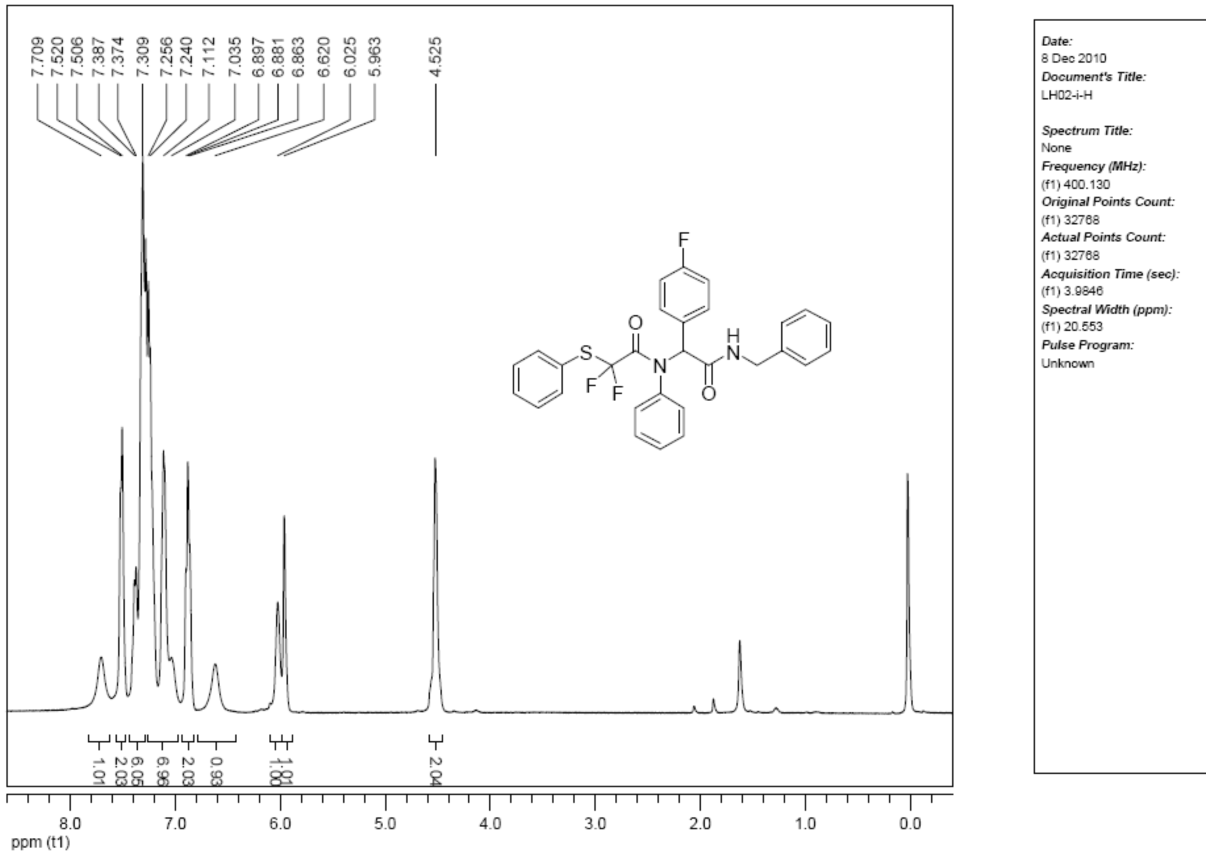

^{13}C NMR of **3i**

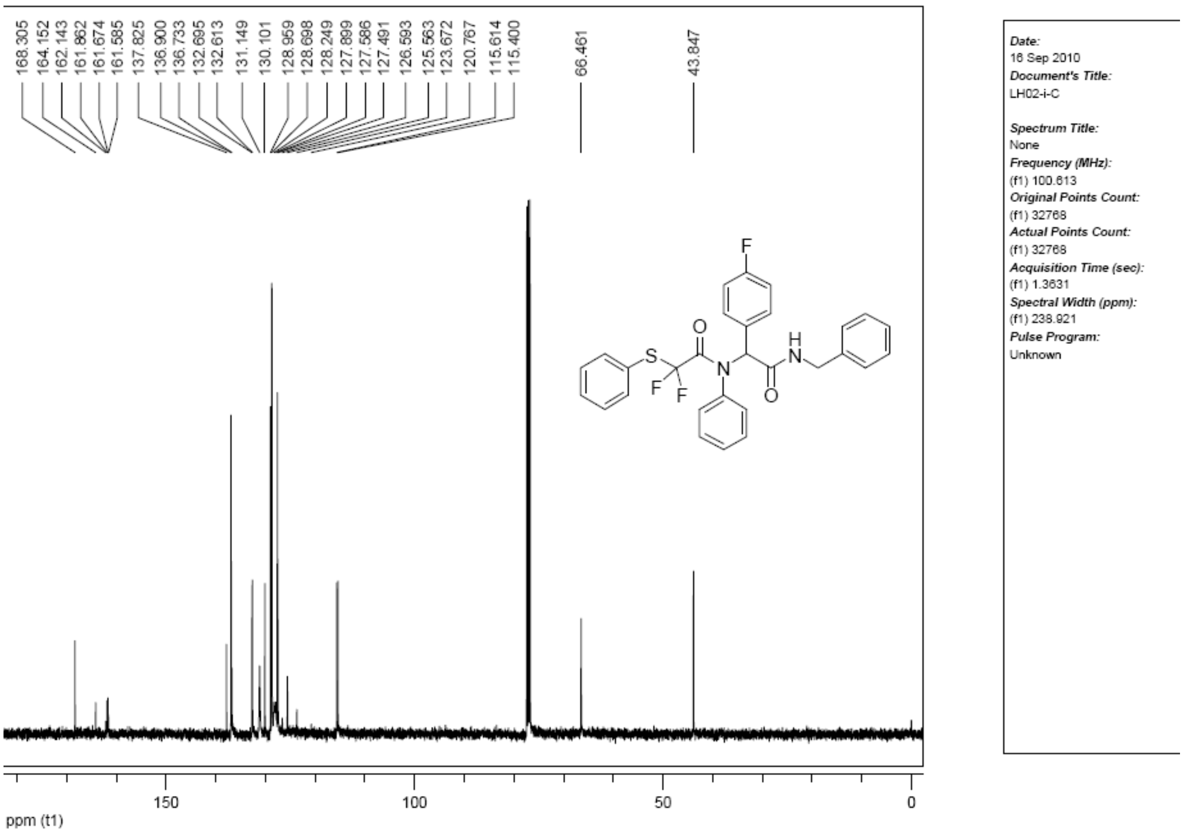

^{19}F NMR of **3i**

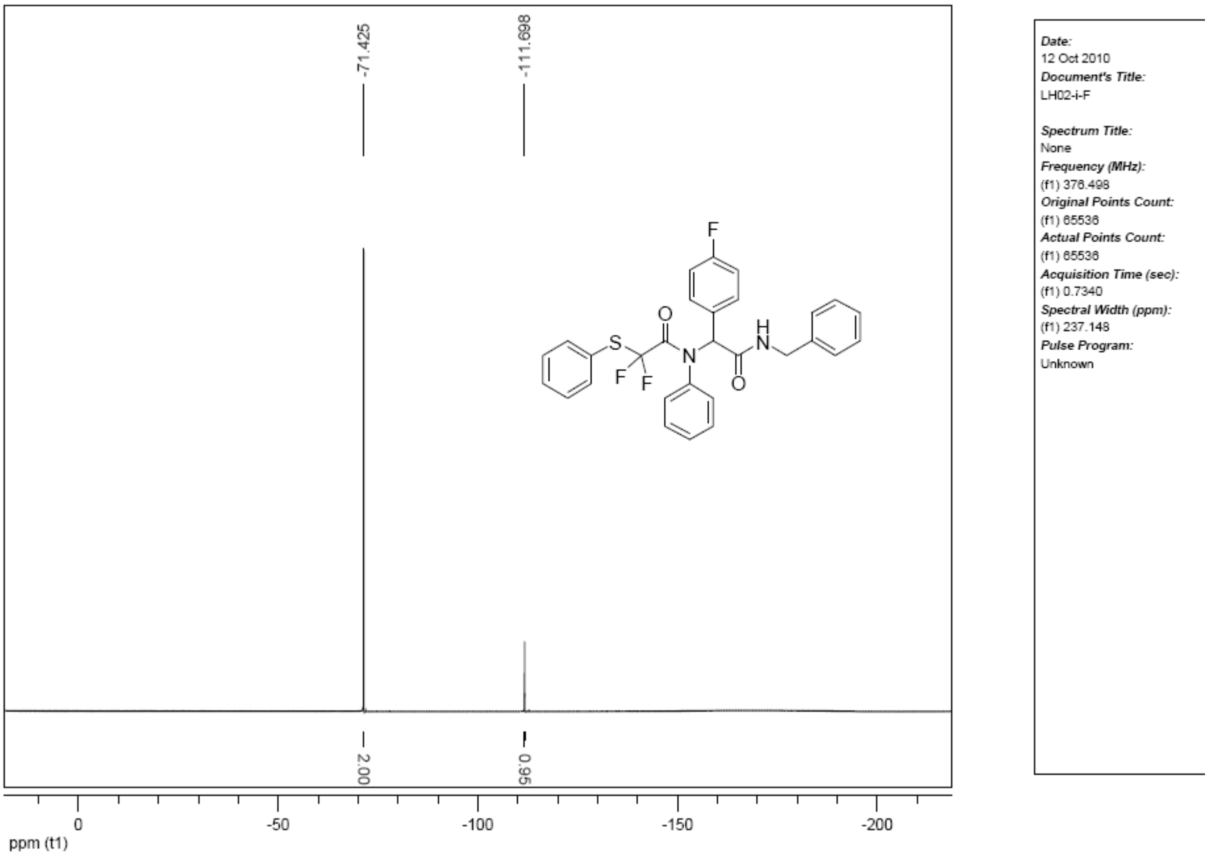

^{1}H NMR of **3j**

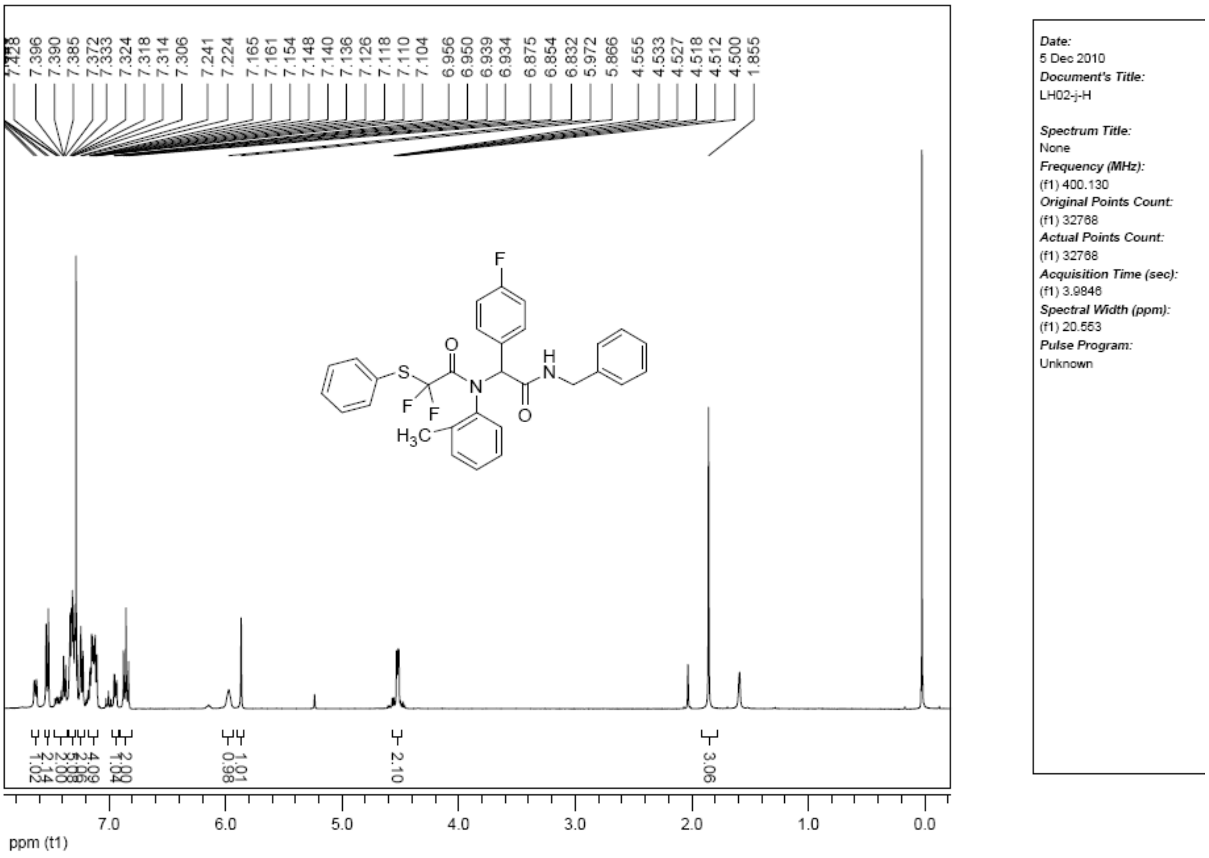

^{13}C NMR of **3j**

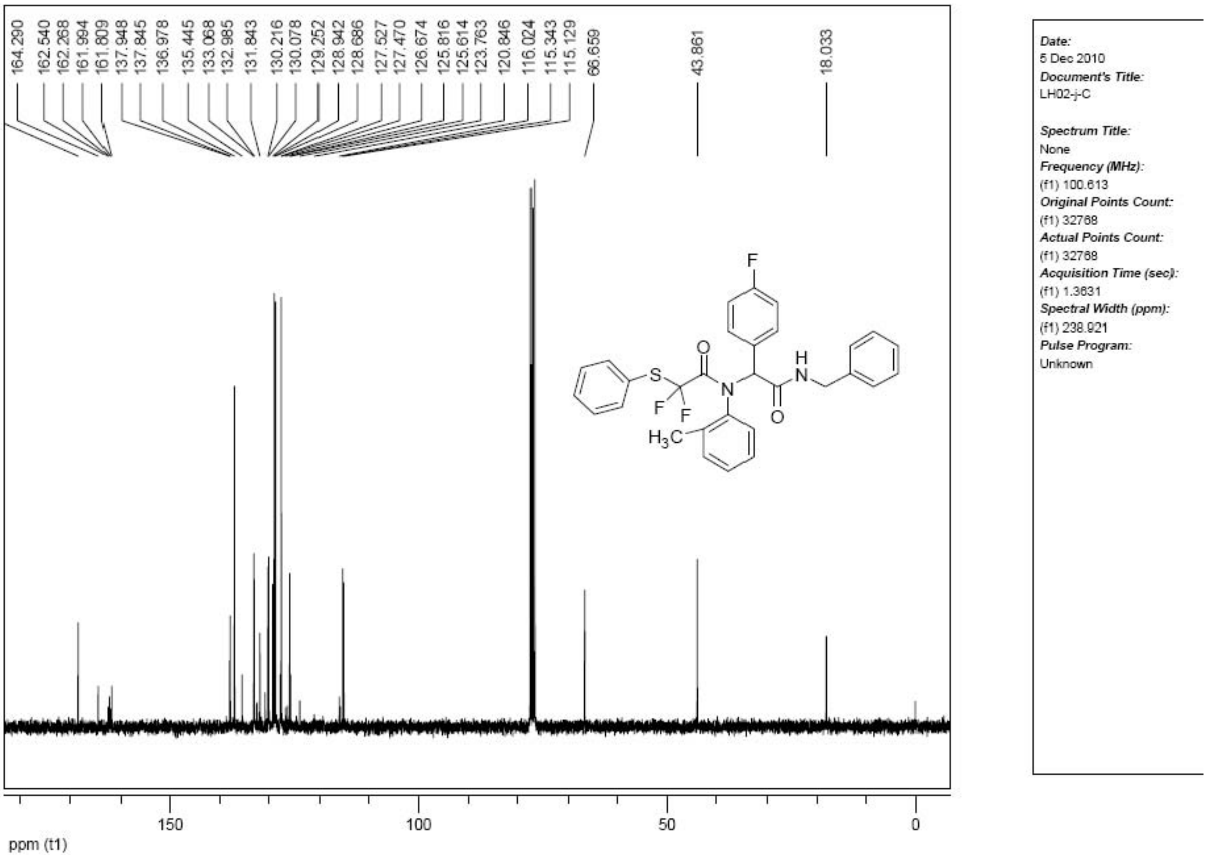

^{19}F NMR of **3j**

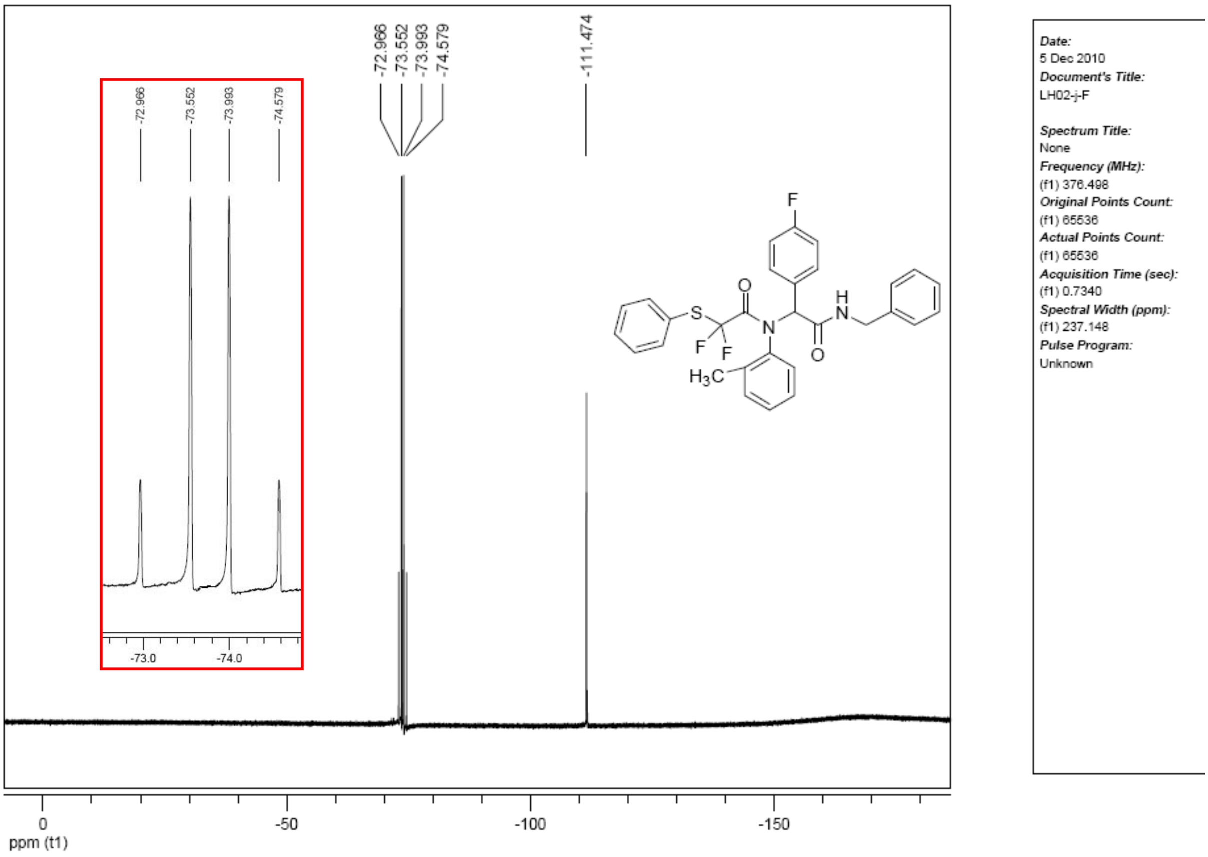

^{1}H NMR of **3k**

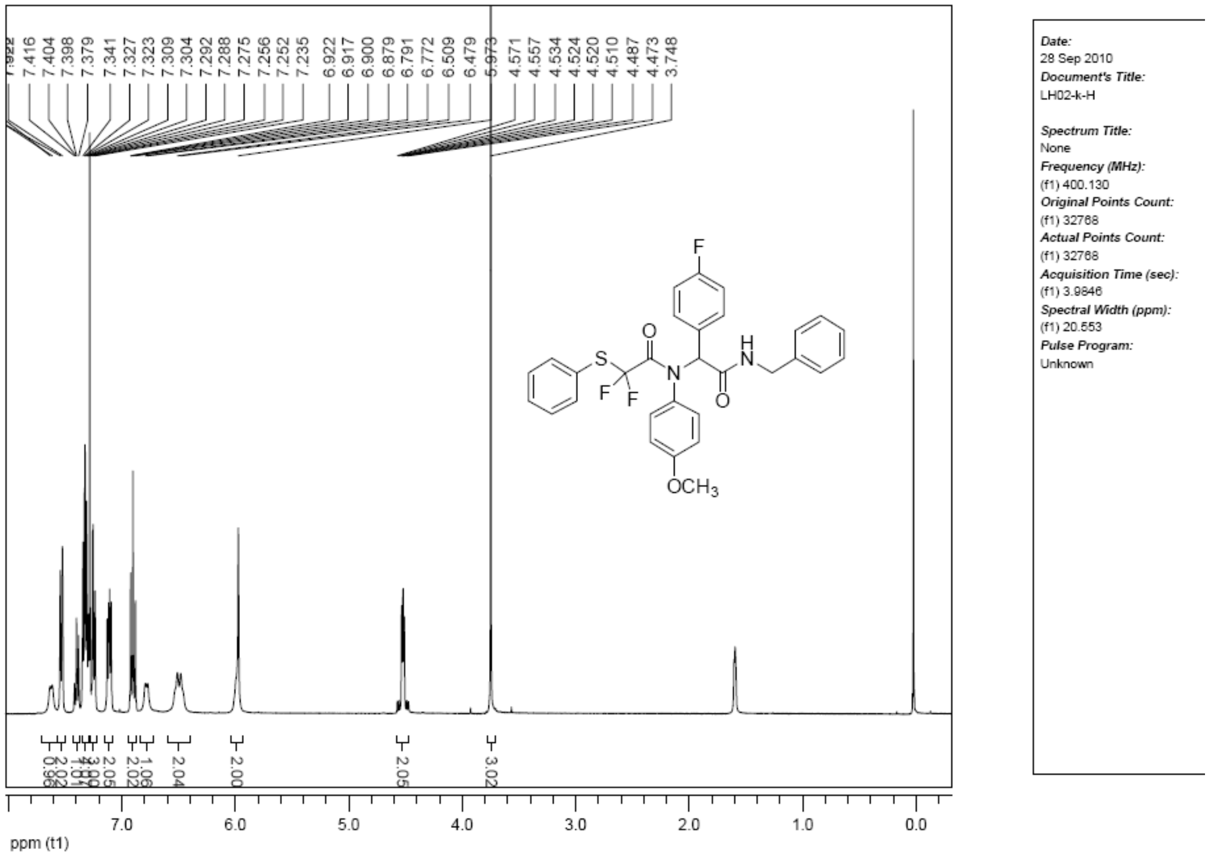

^{13}C NMR of **3k**

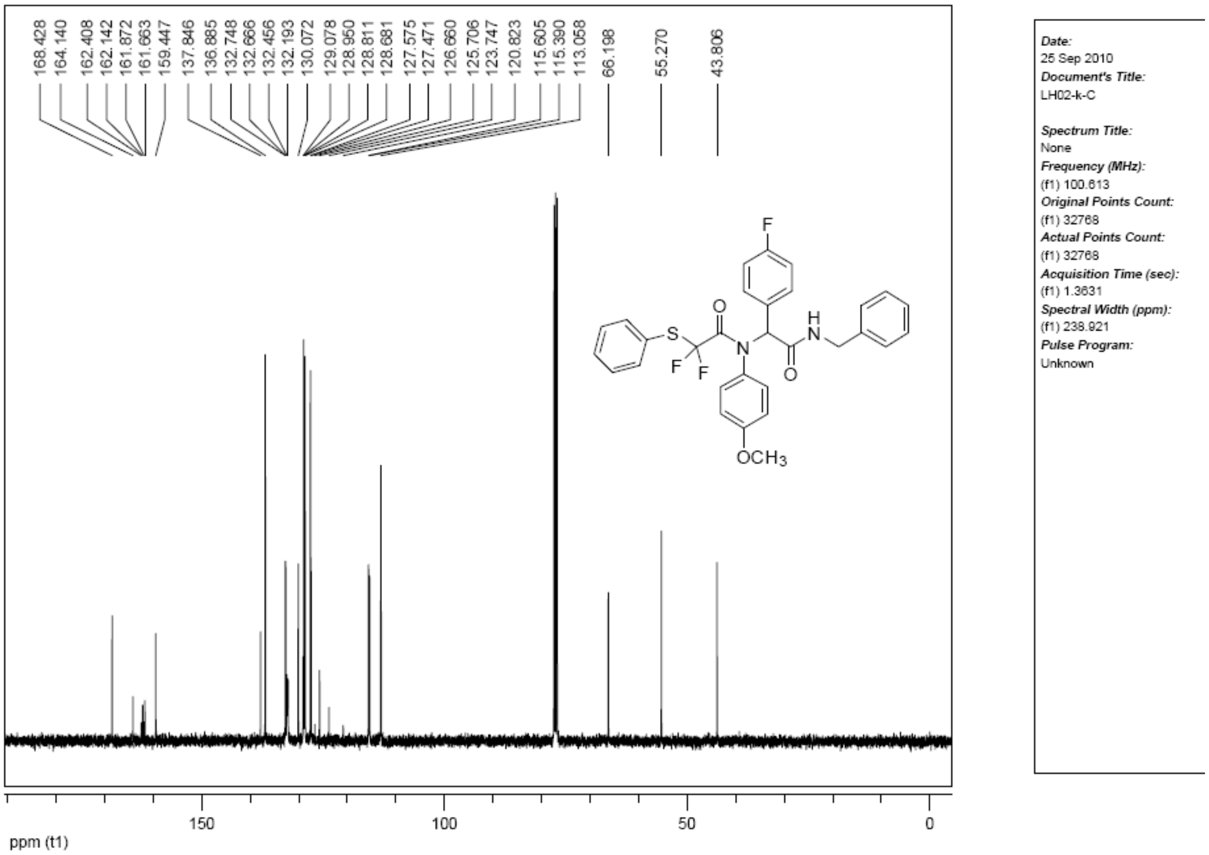

^{19}F NMR of **3k**

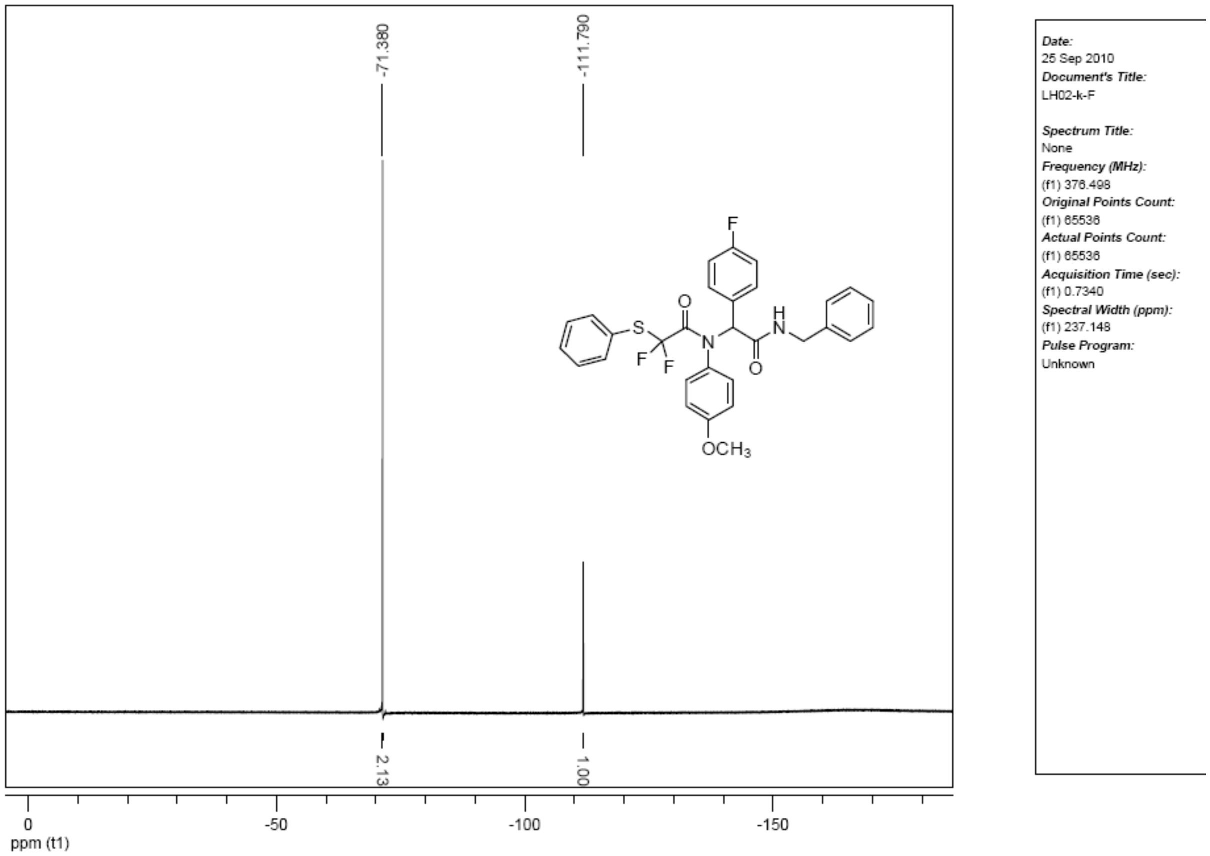

^{1}H NMR of **3l**

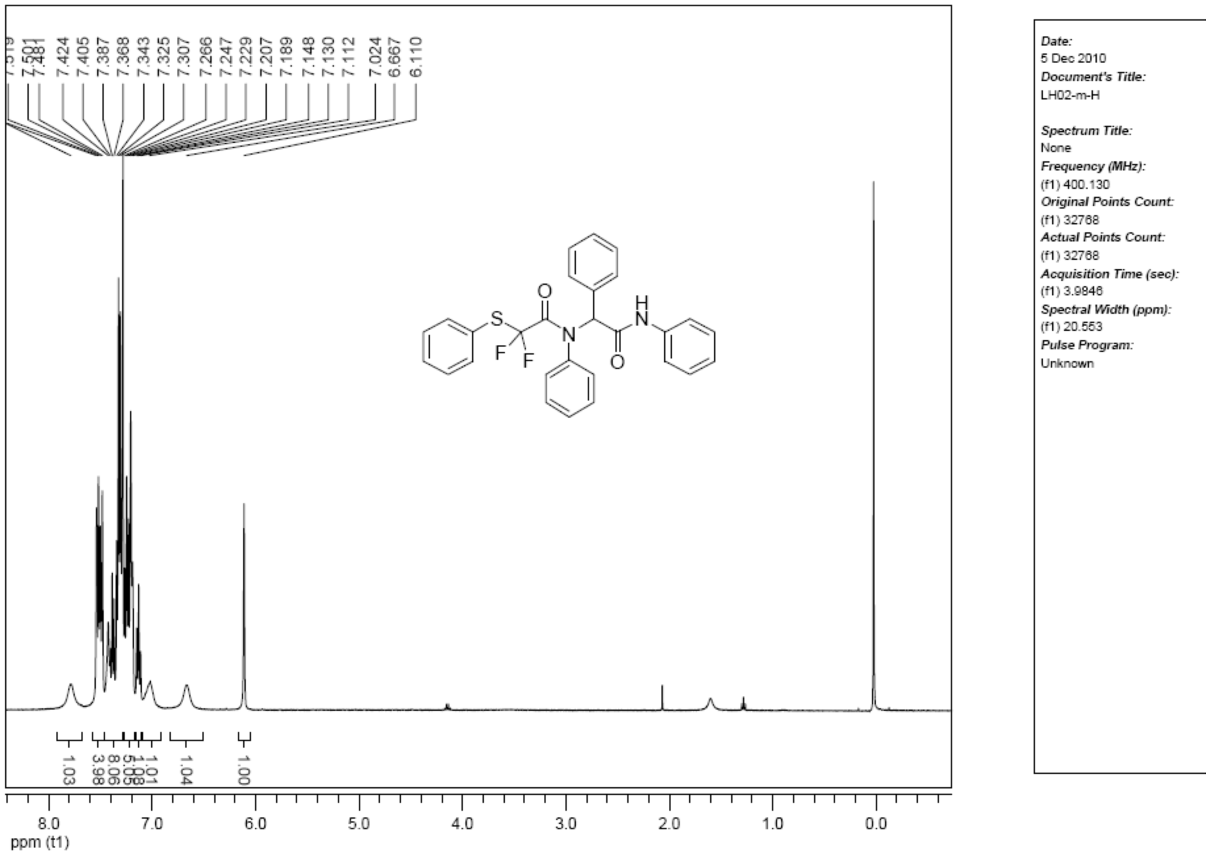

^{13}C NMR of **3l**

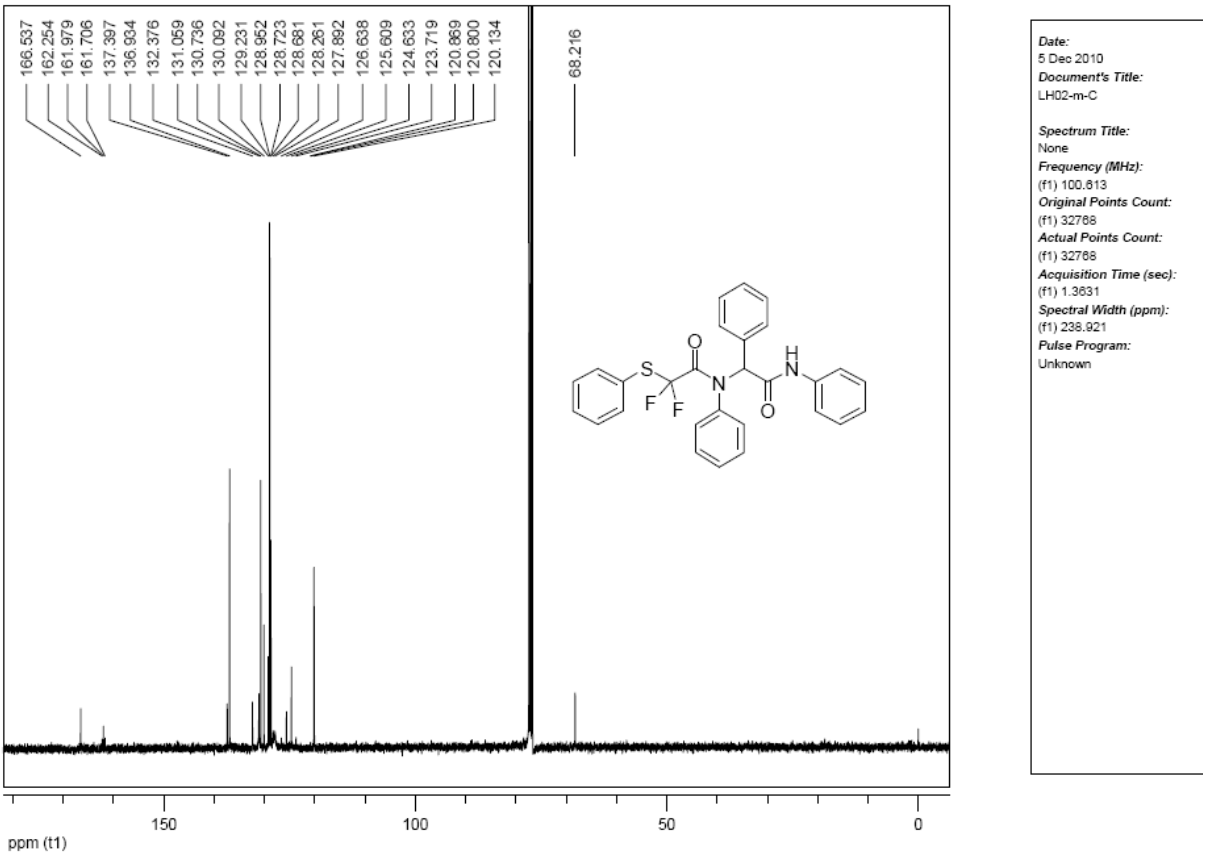

^{19}F NMR of **3l**

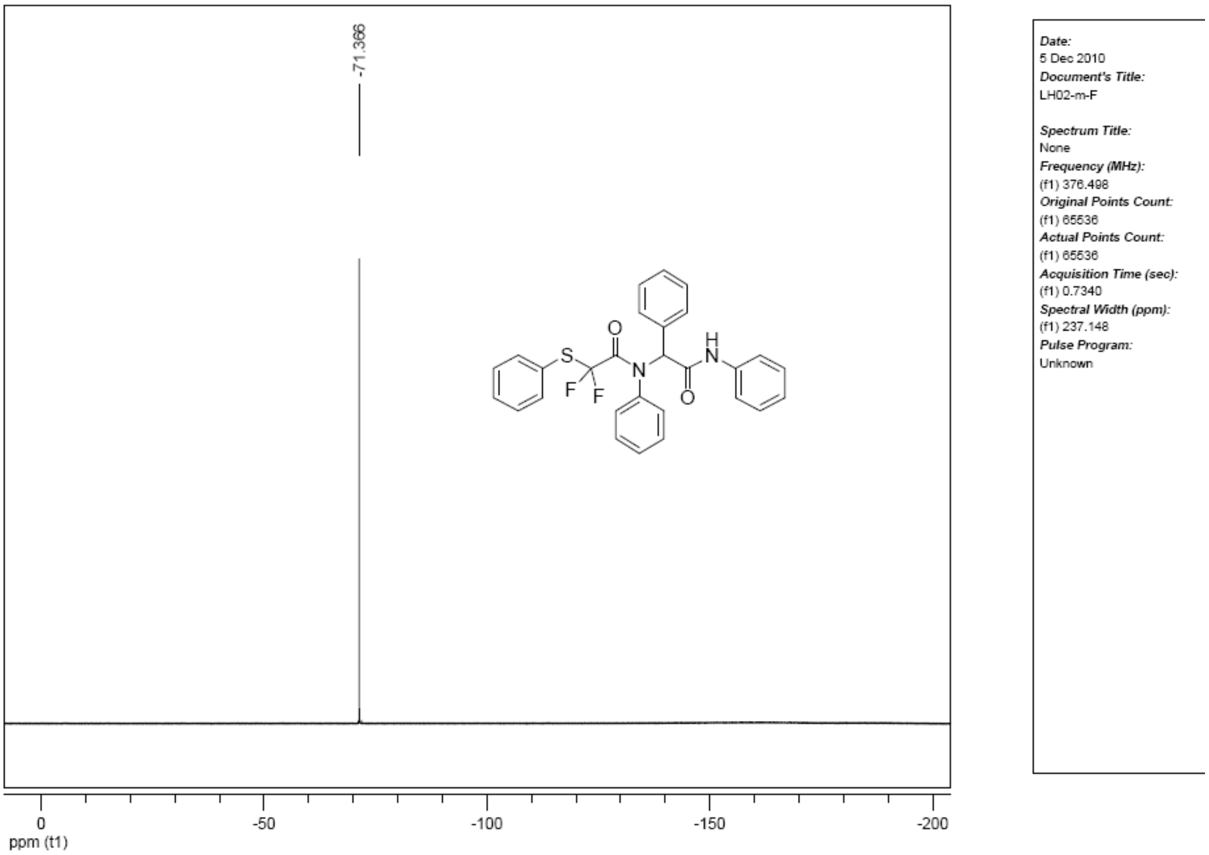

^{1}H NMR of **3m**

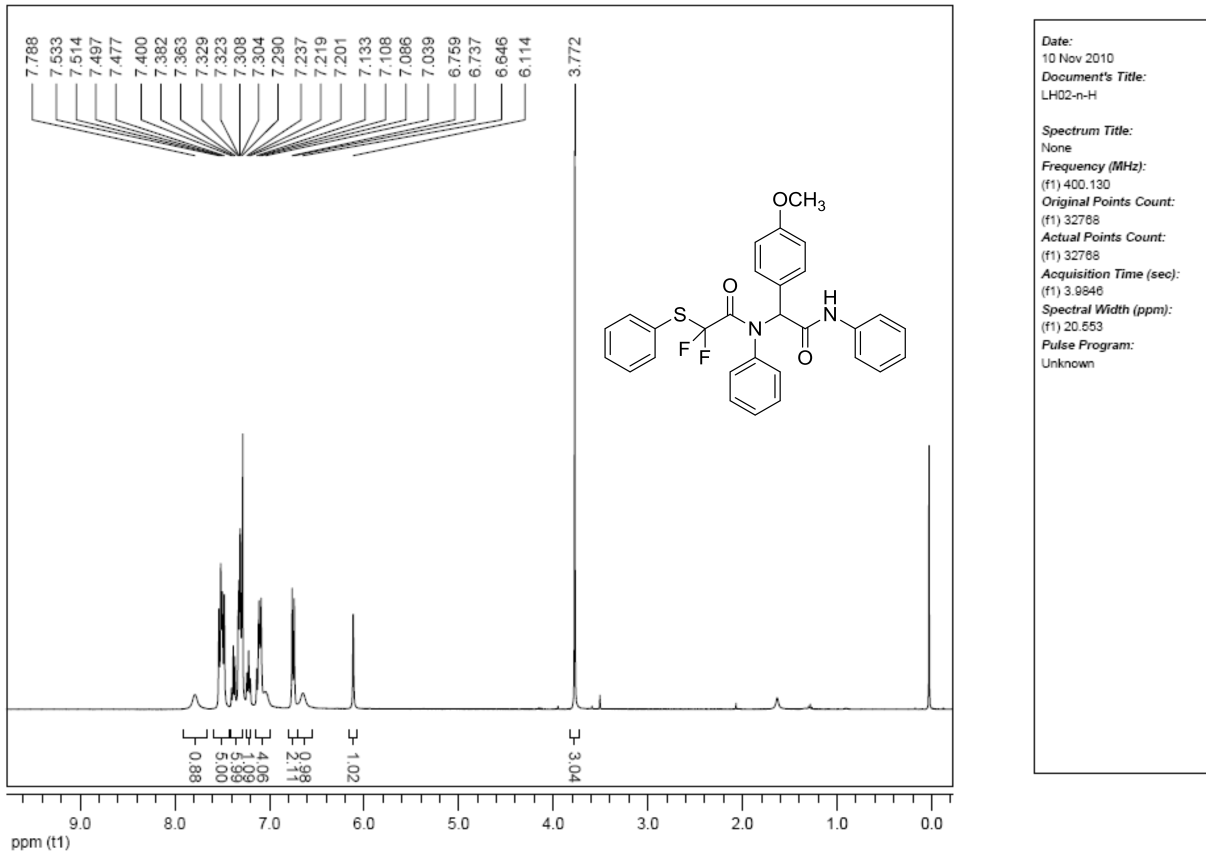

^{13}C NMR of **3m**

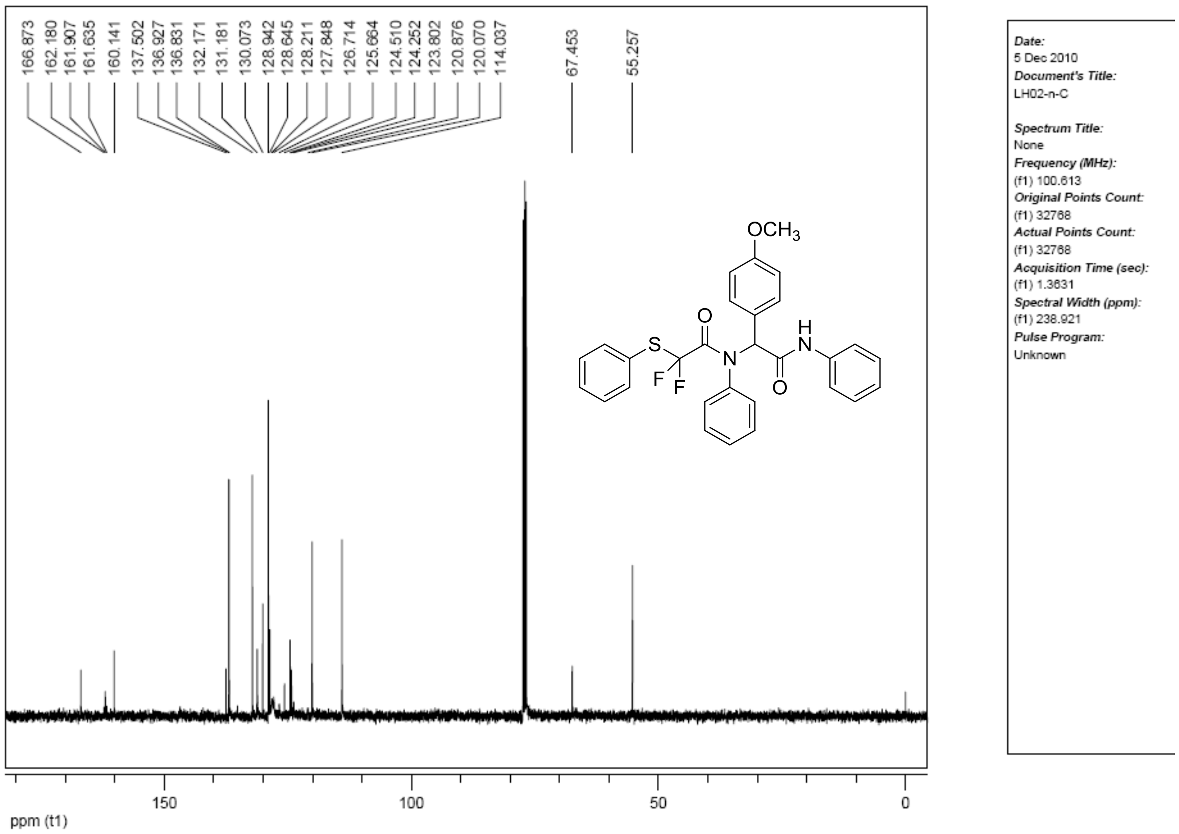

^{19}F NMR of **3m**

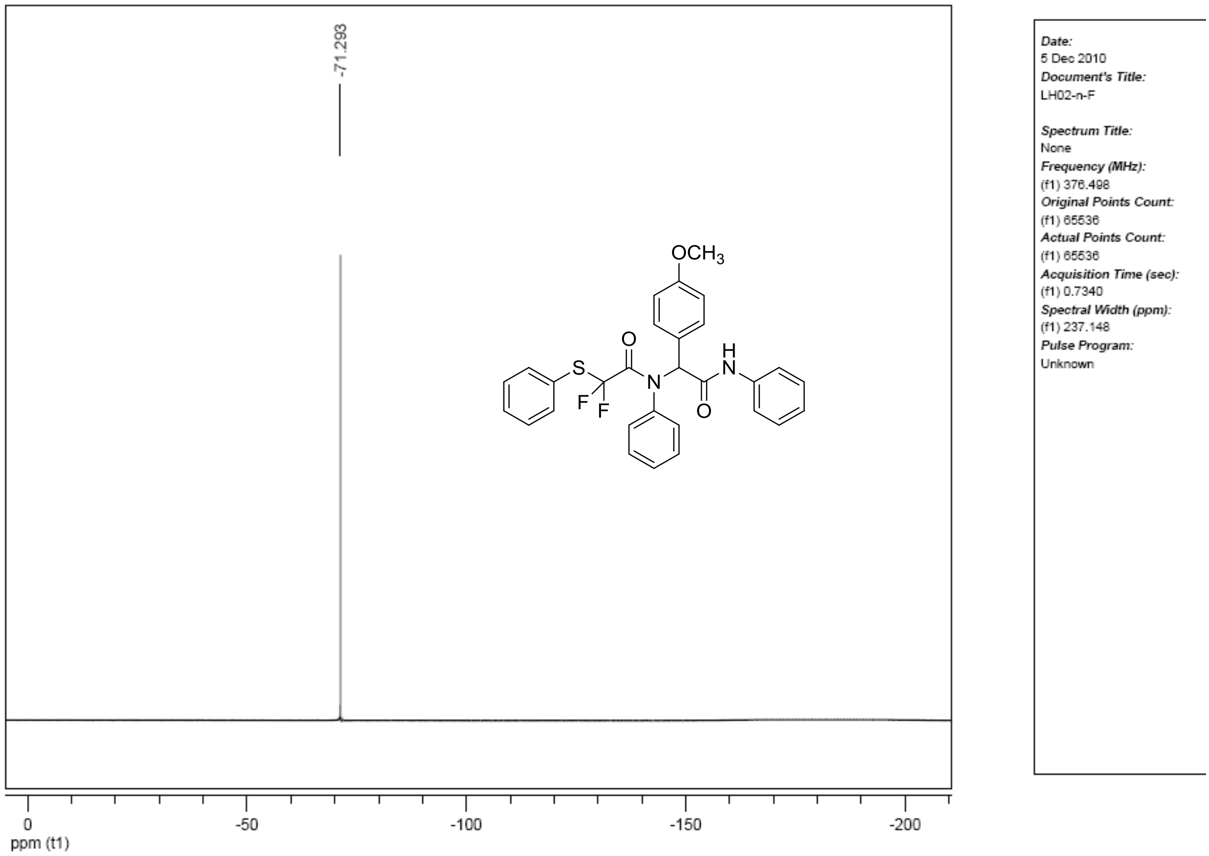

^{1}H NMR of **4a**

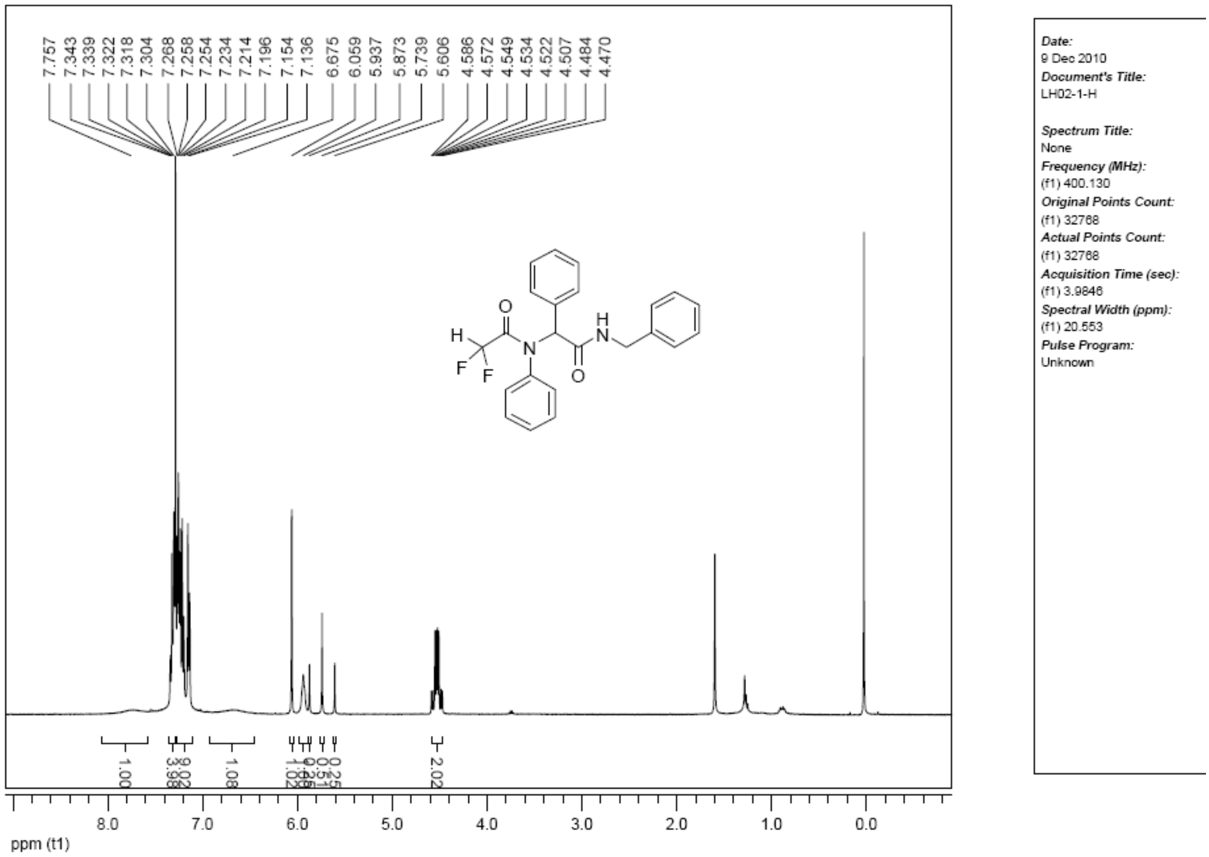

^{13}C NMR of **4a**

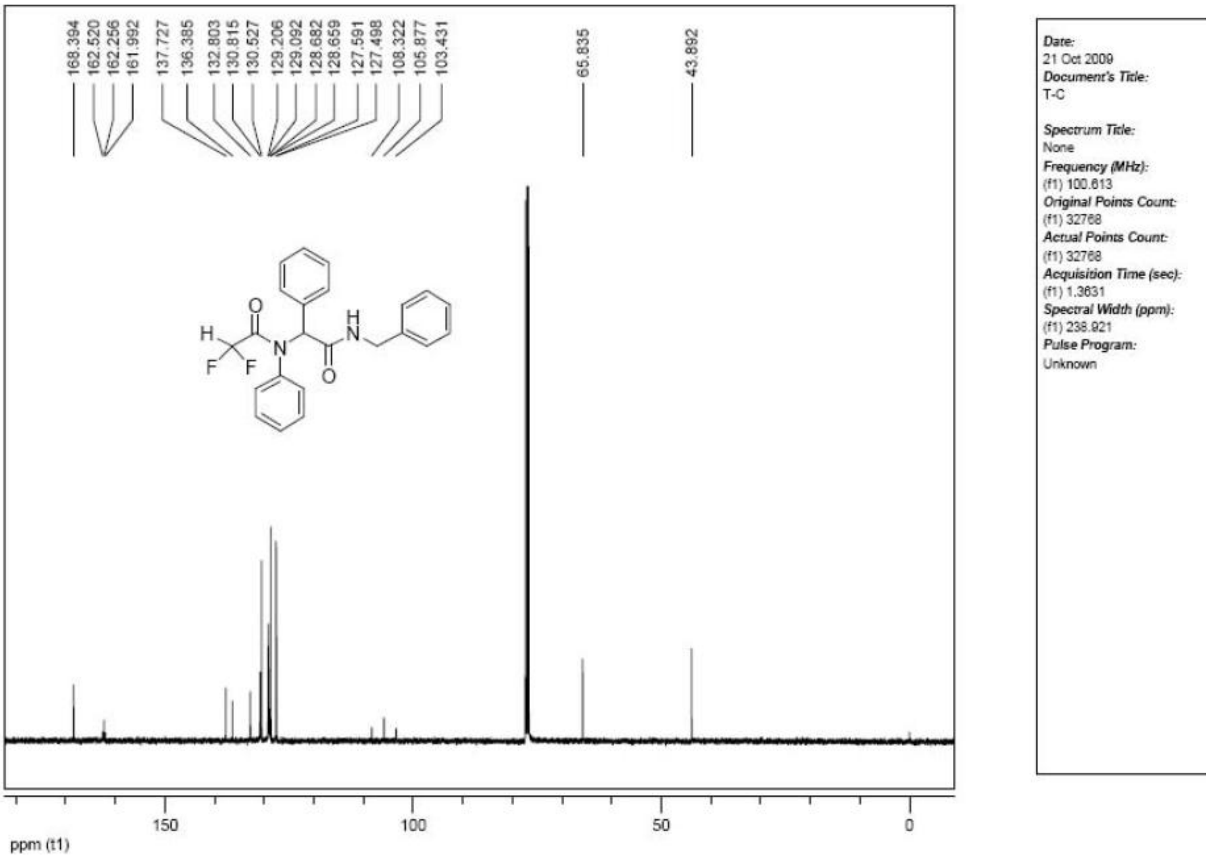

^{19}F NMR of **4a**

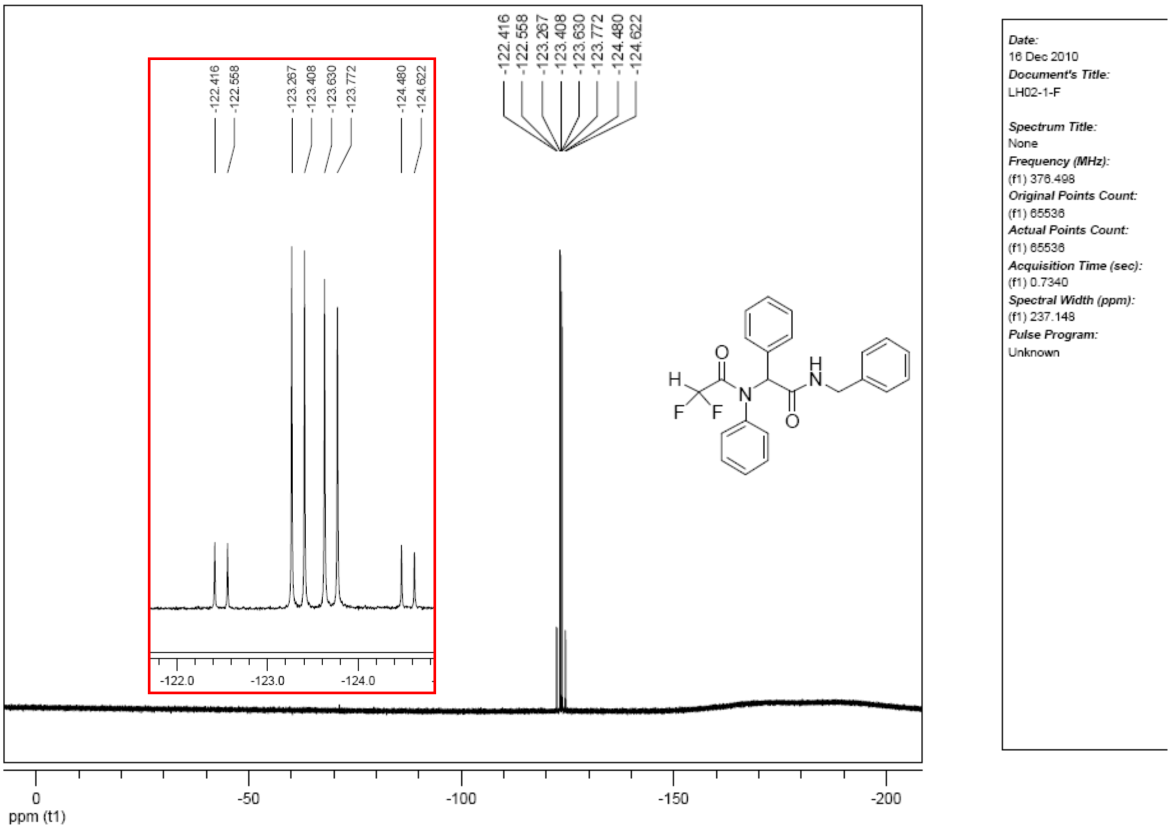

MS (ESI) of **4a**

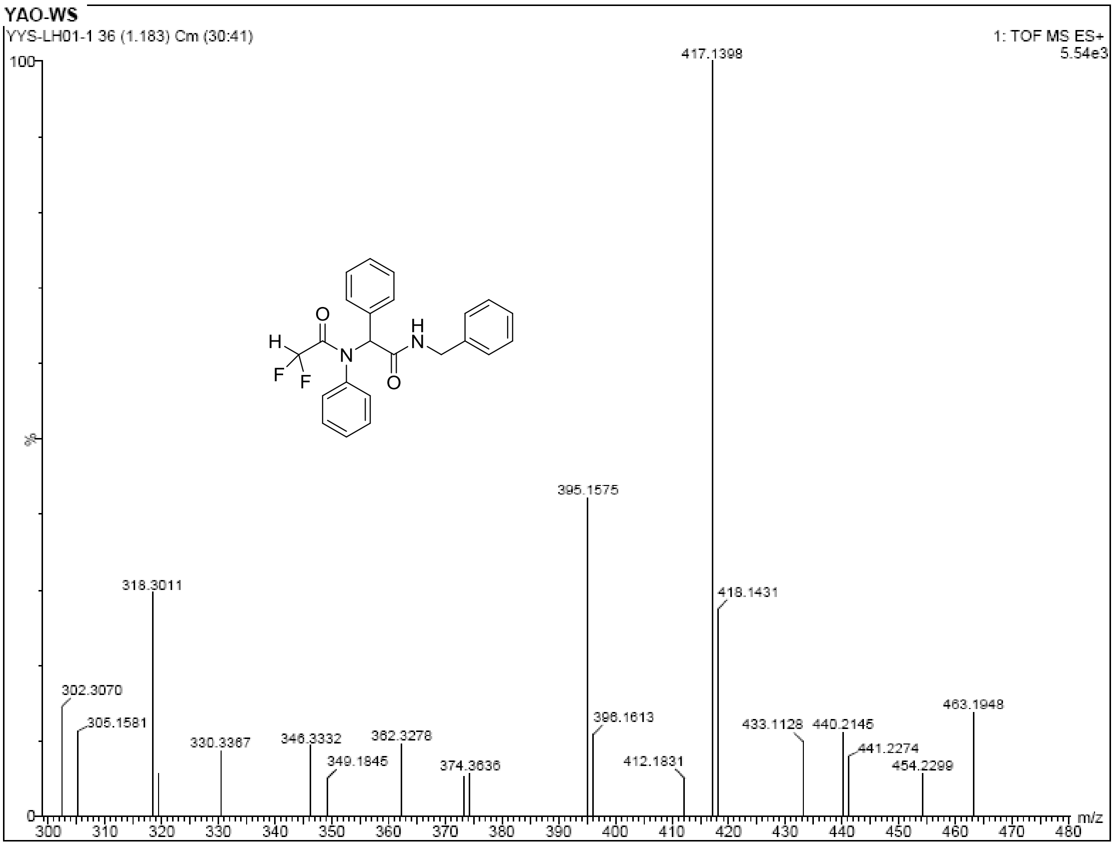

^{1}H NMR of **4b**

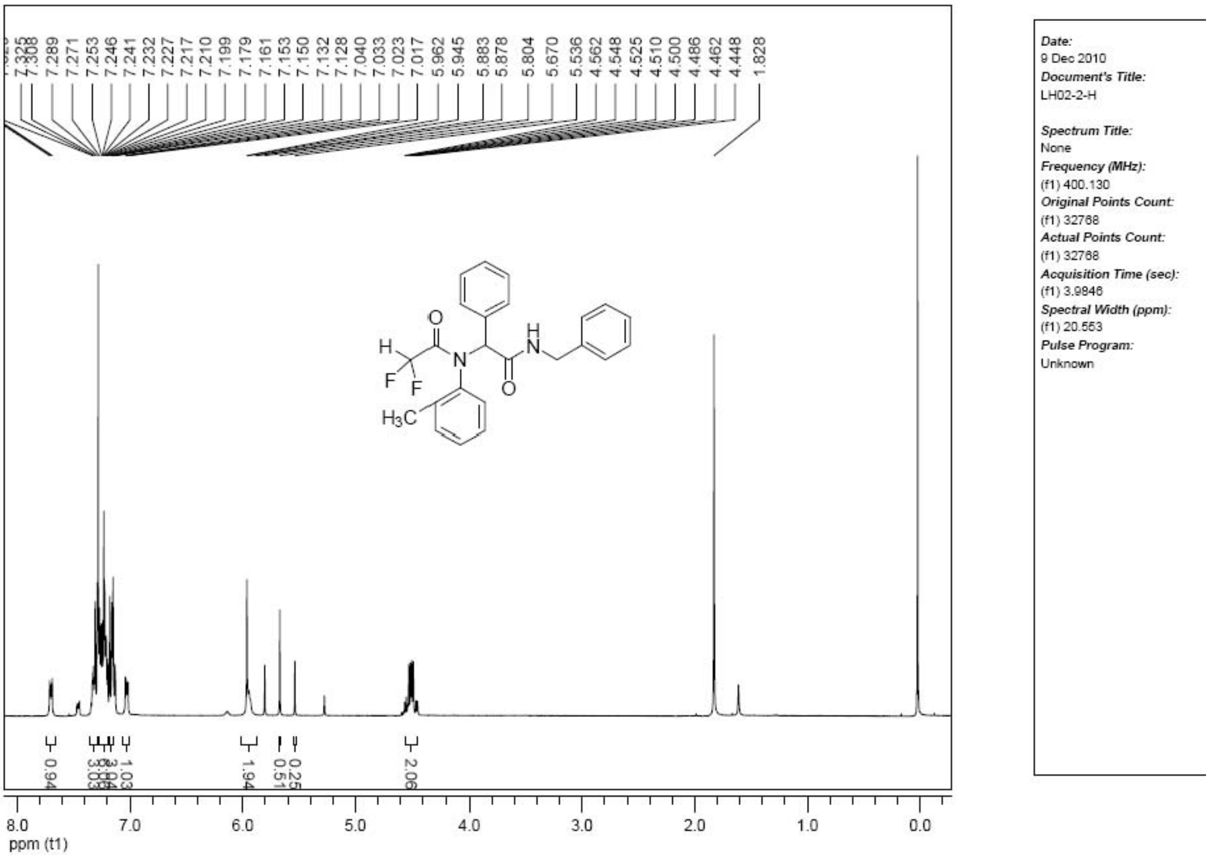

^{13}C NMR of **4b**

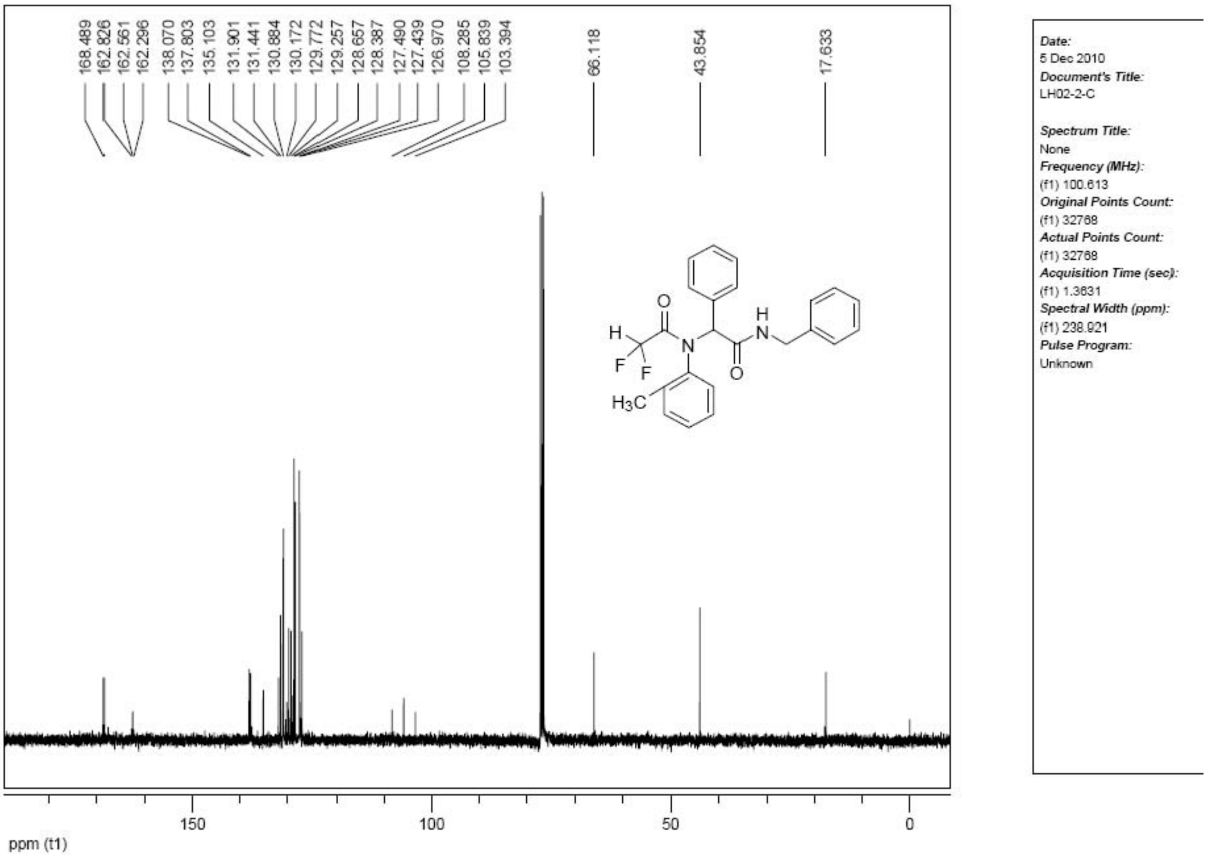

^{19}F NMR of **4b**

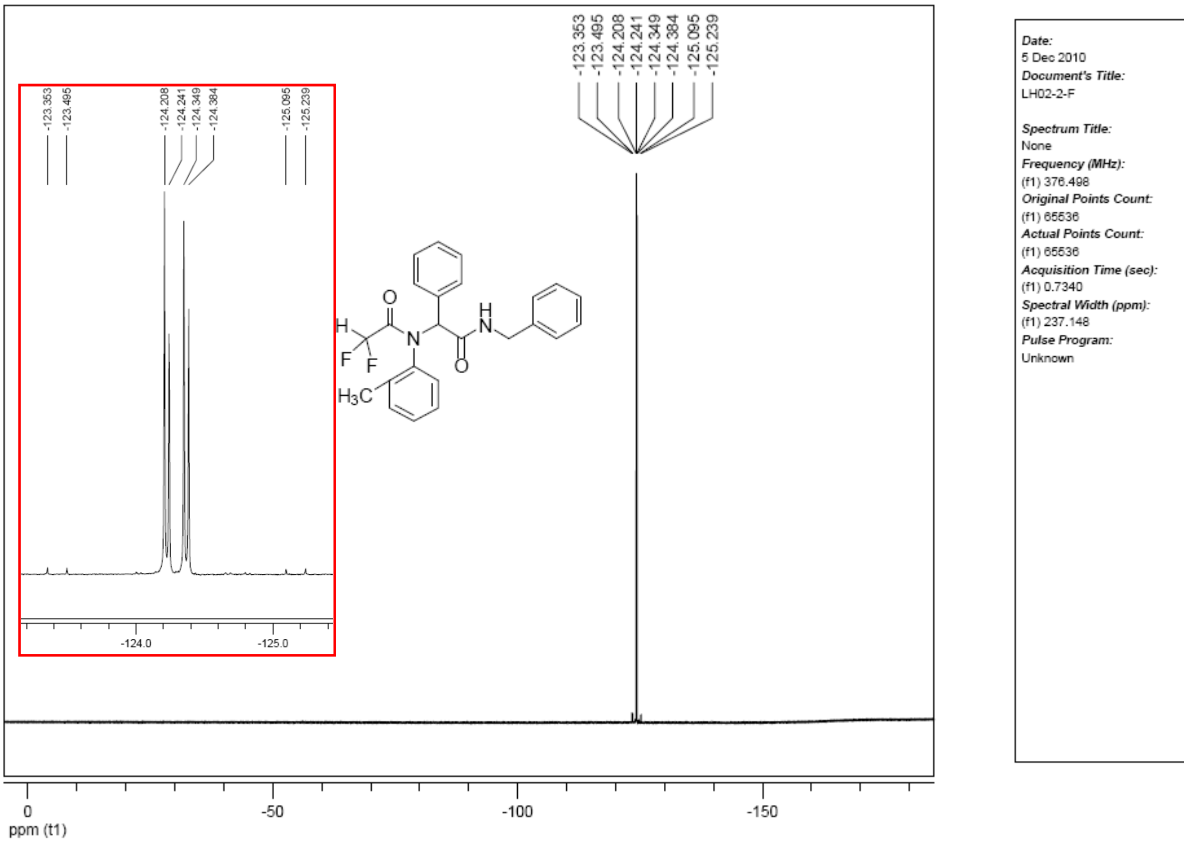

MS (ESI) of **4b**

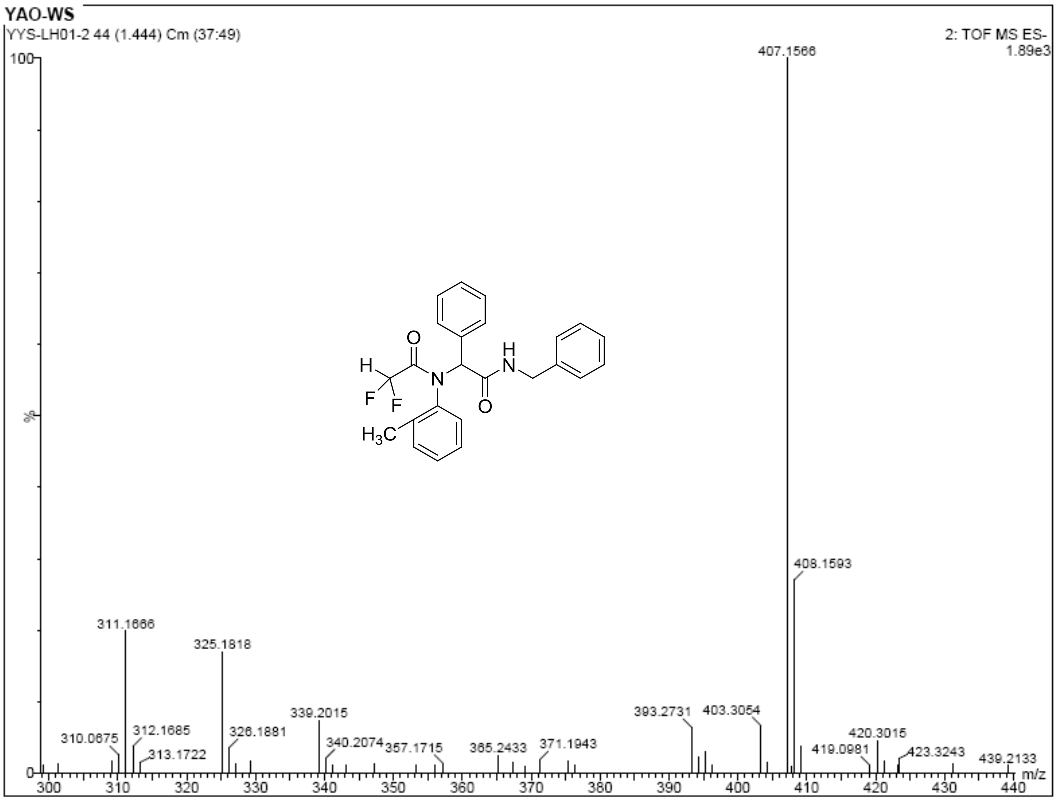

^{1}H NMR of **4c**

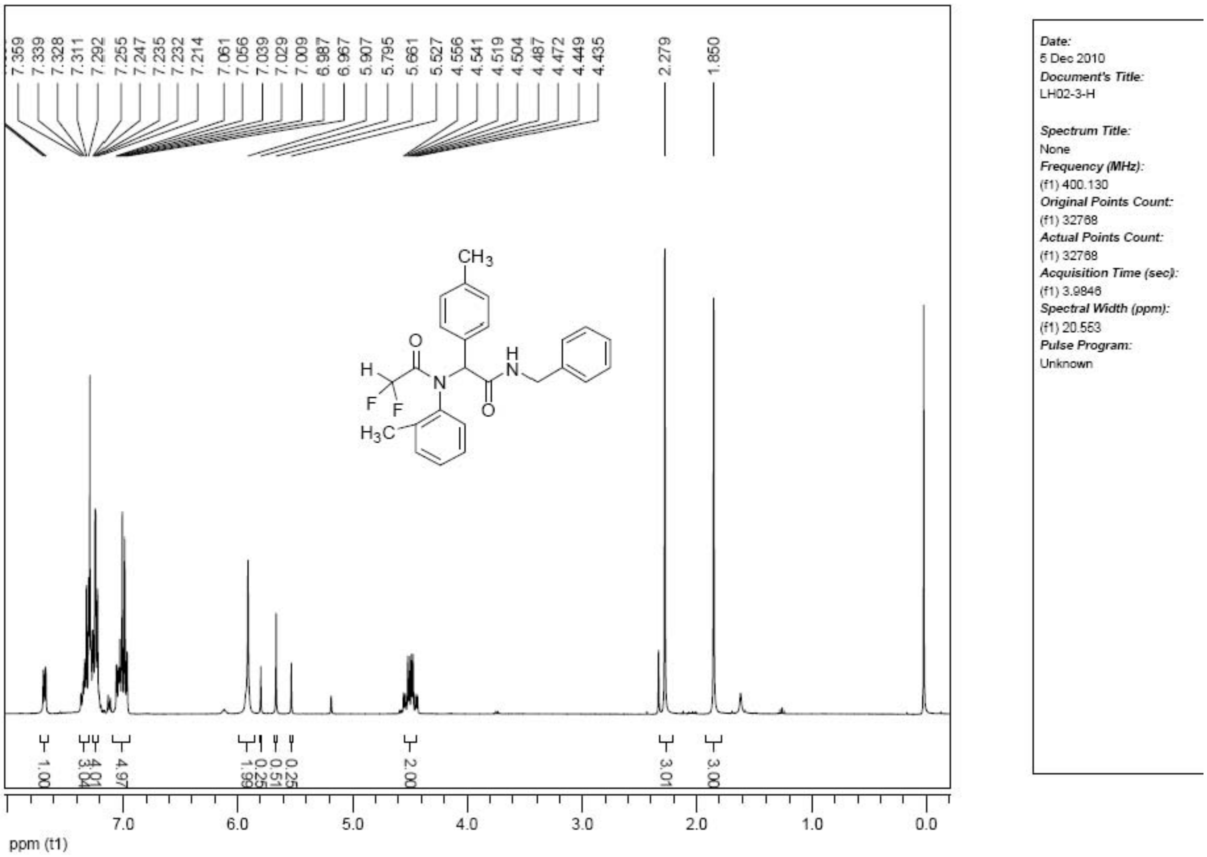

^{13}C NMR of **4c**

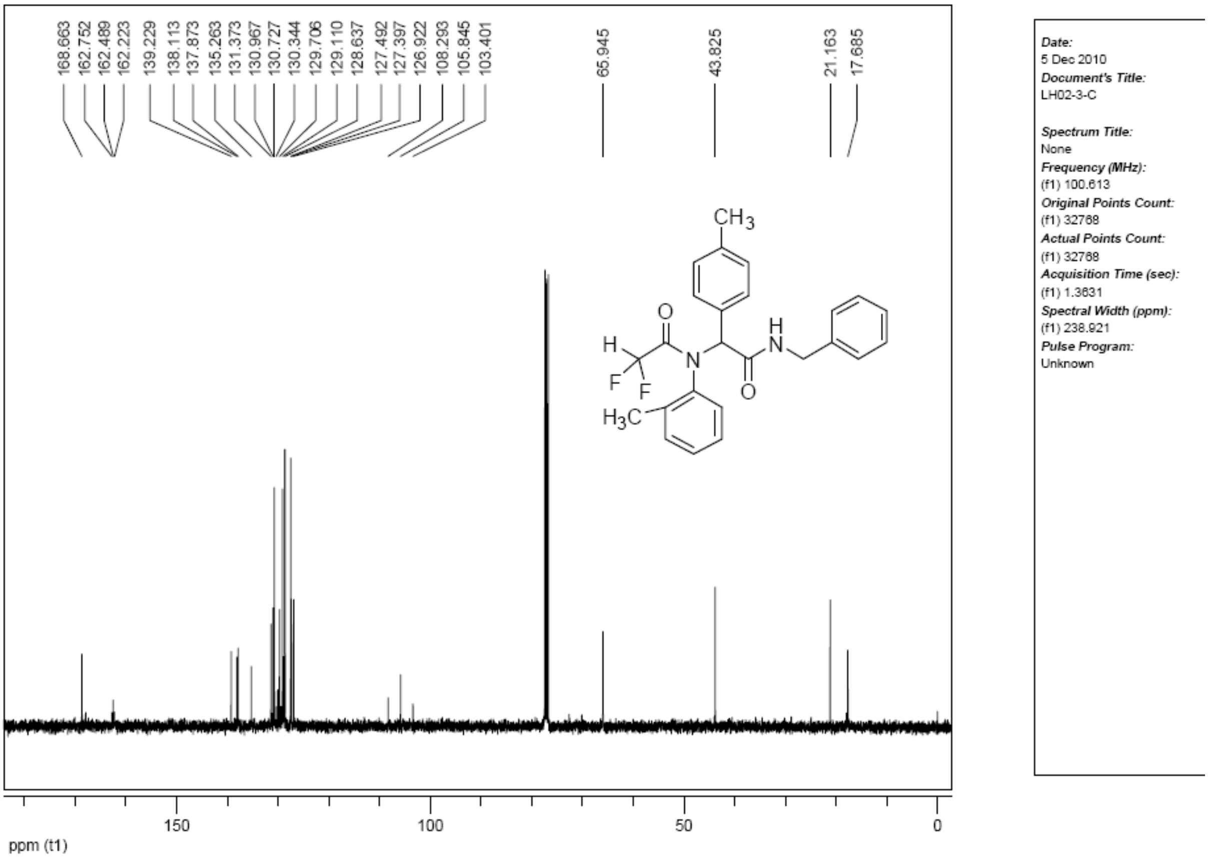

^{19}F NMR of **4c**

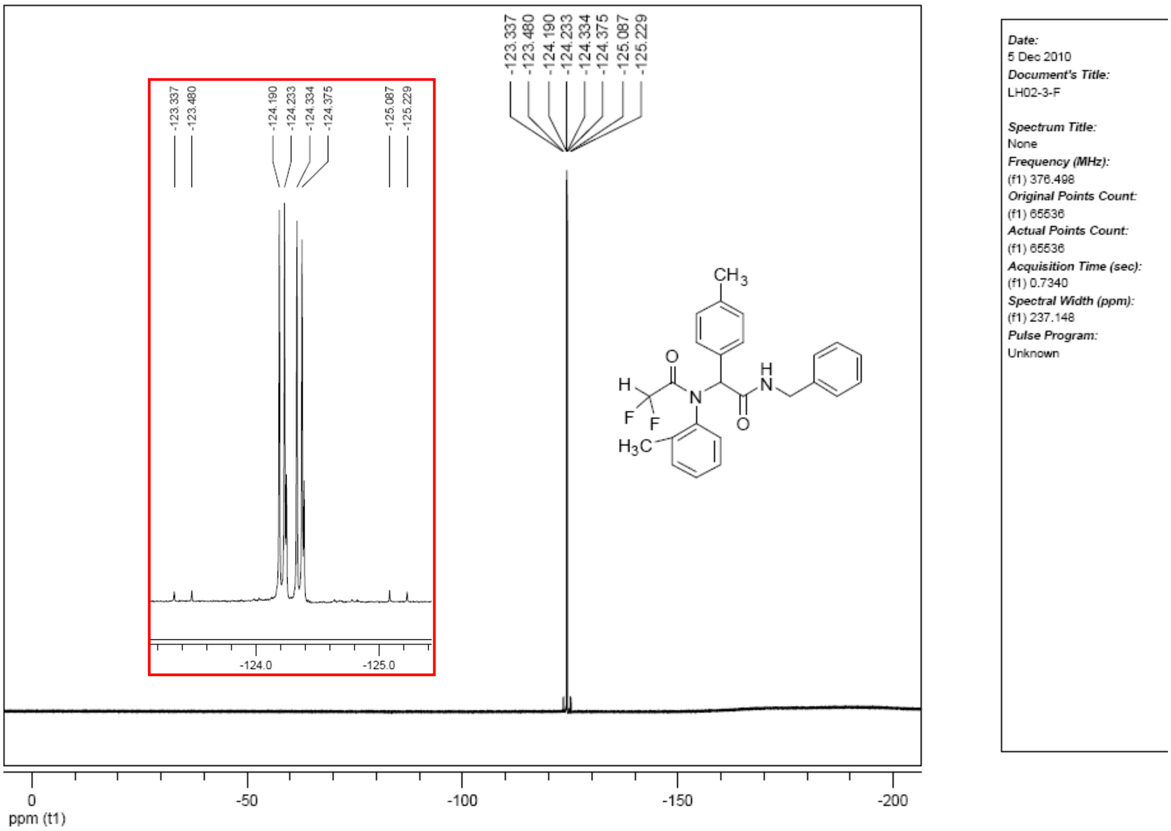

MS (ESI) of **4c**

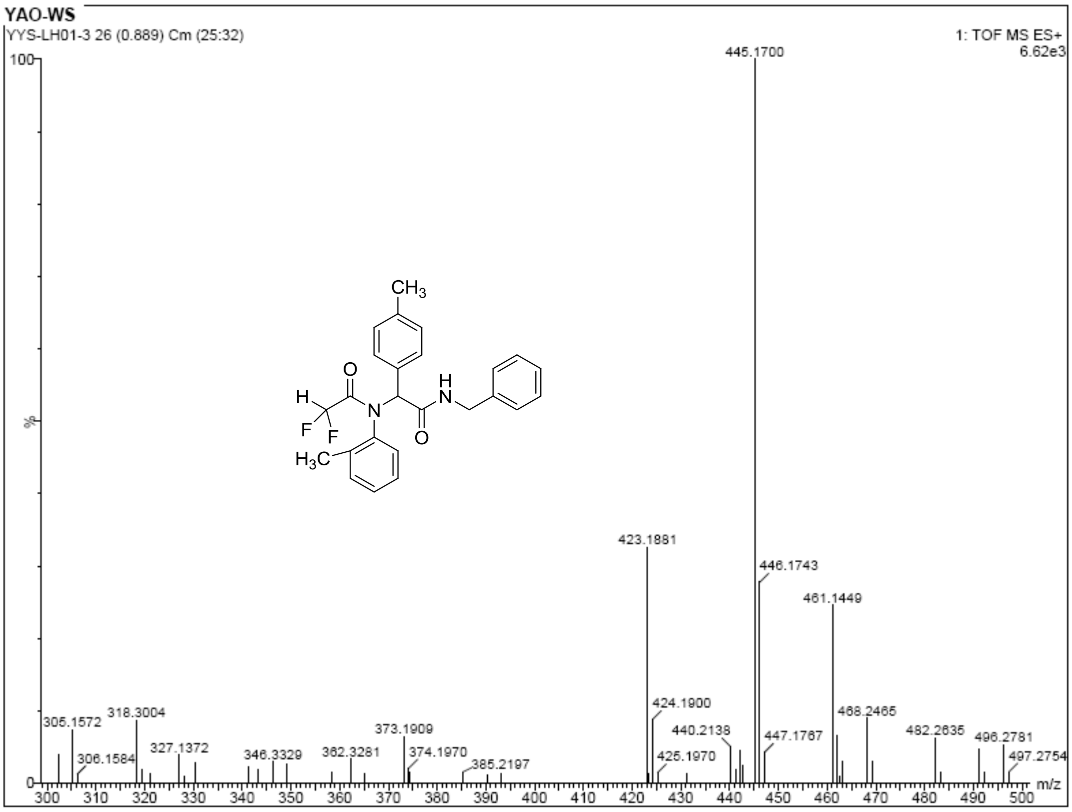

^{1}H NMR of **4d**

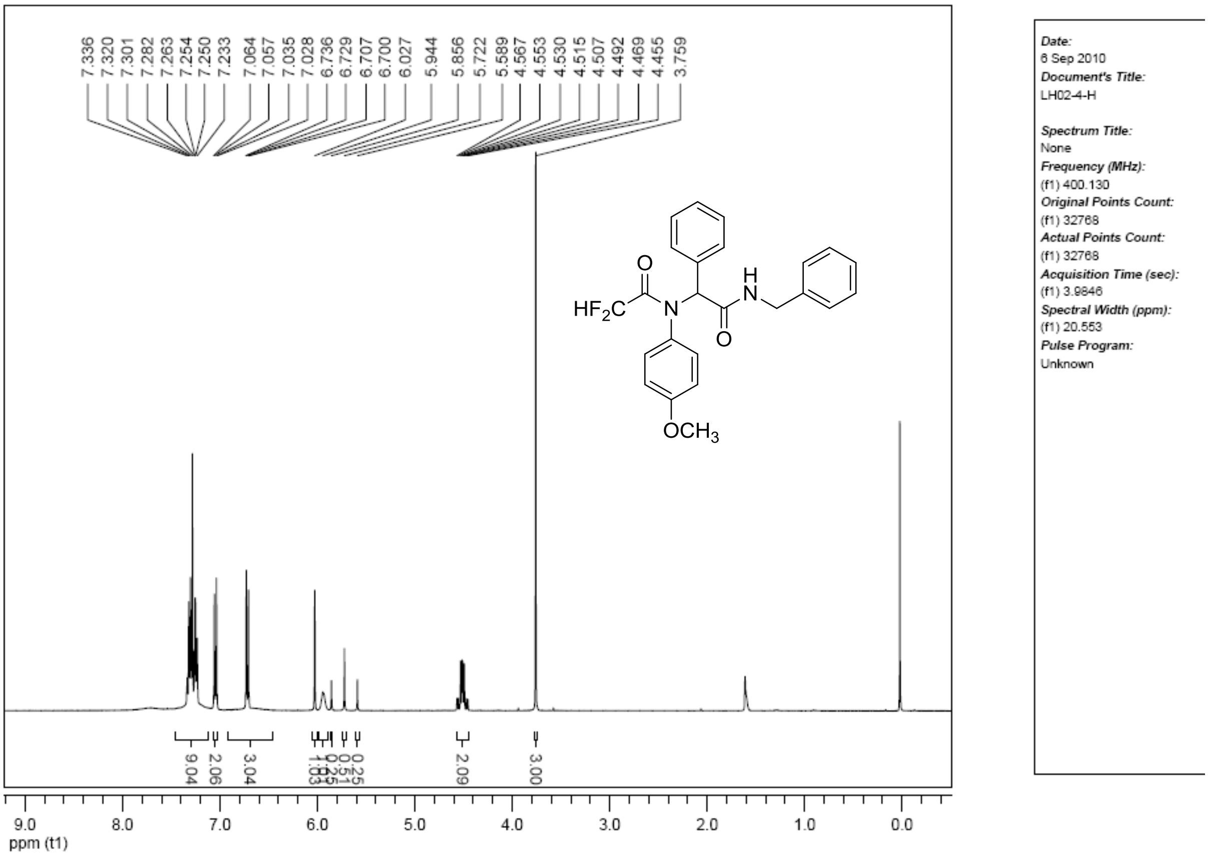

^{13}C NMR of **4d**

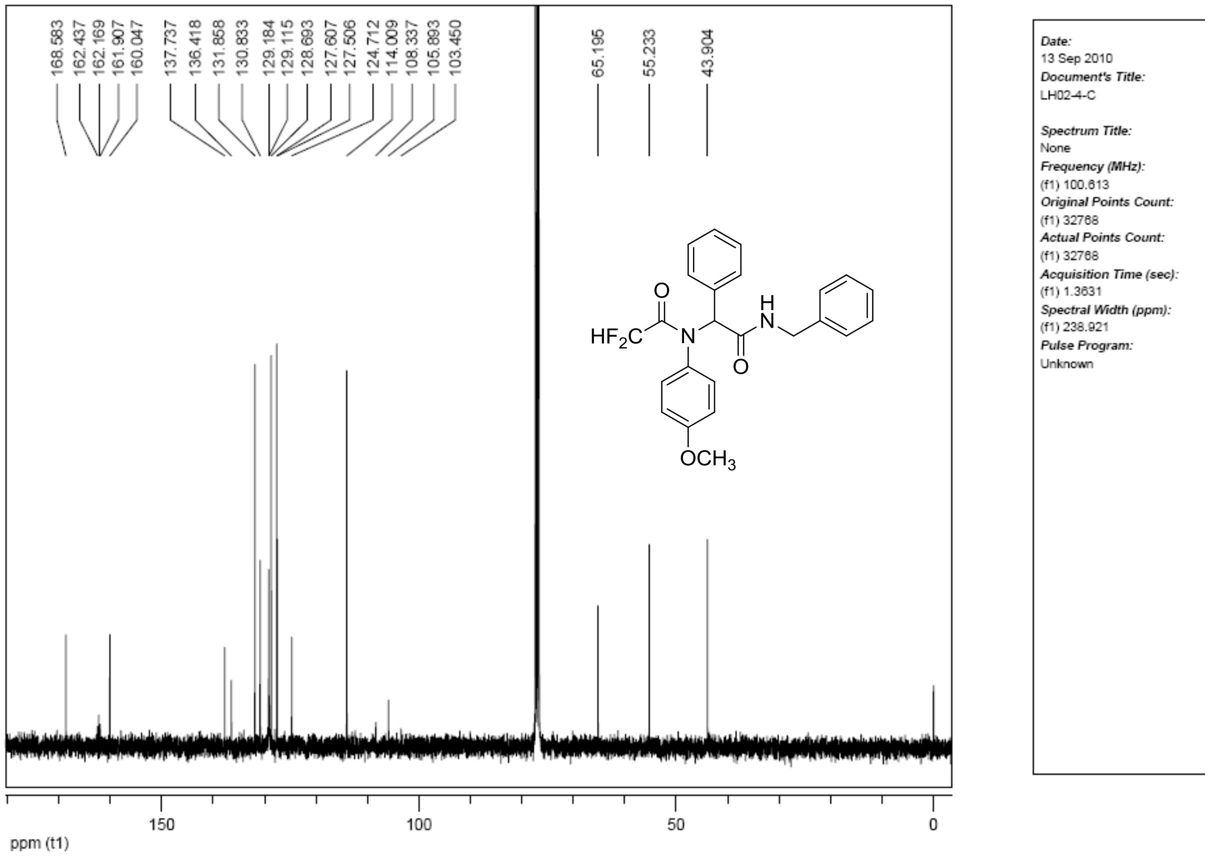

^{19}F NMR of **4d**

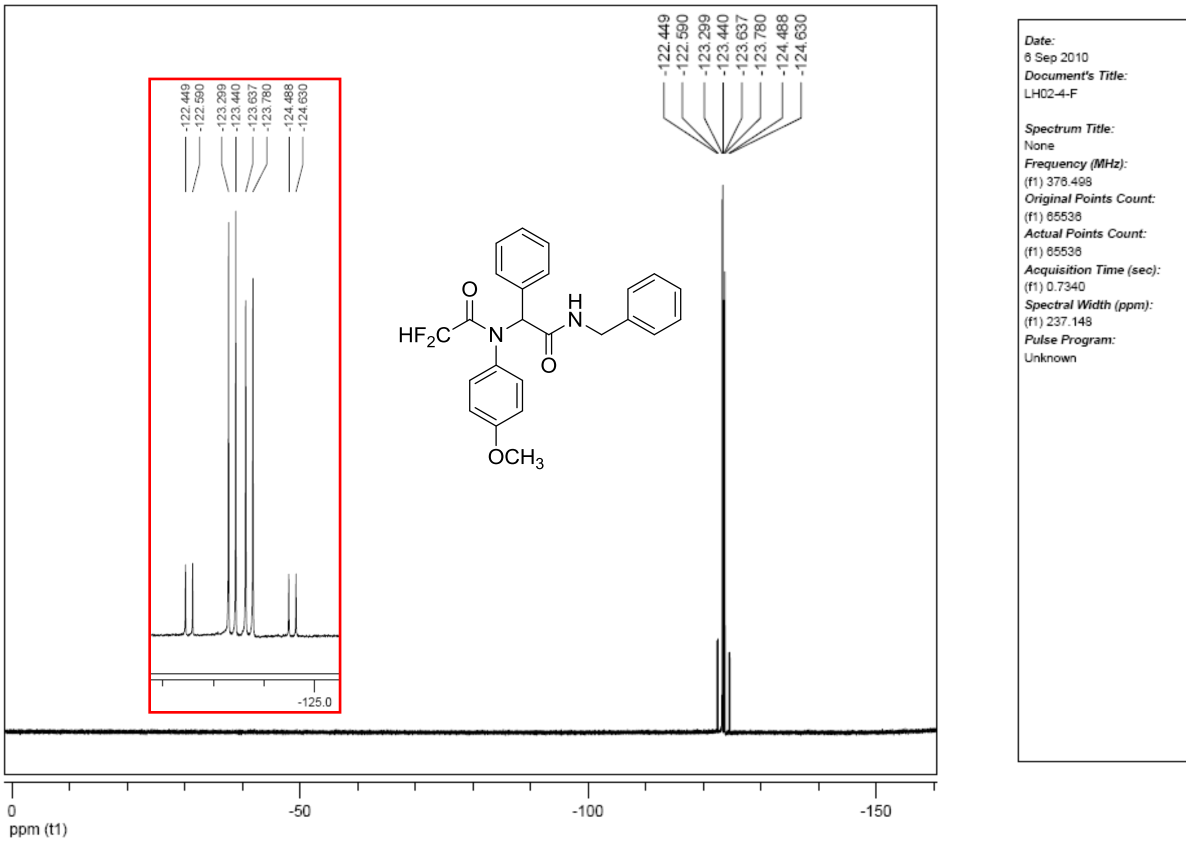

MS (ESI) of **4d**

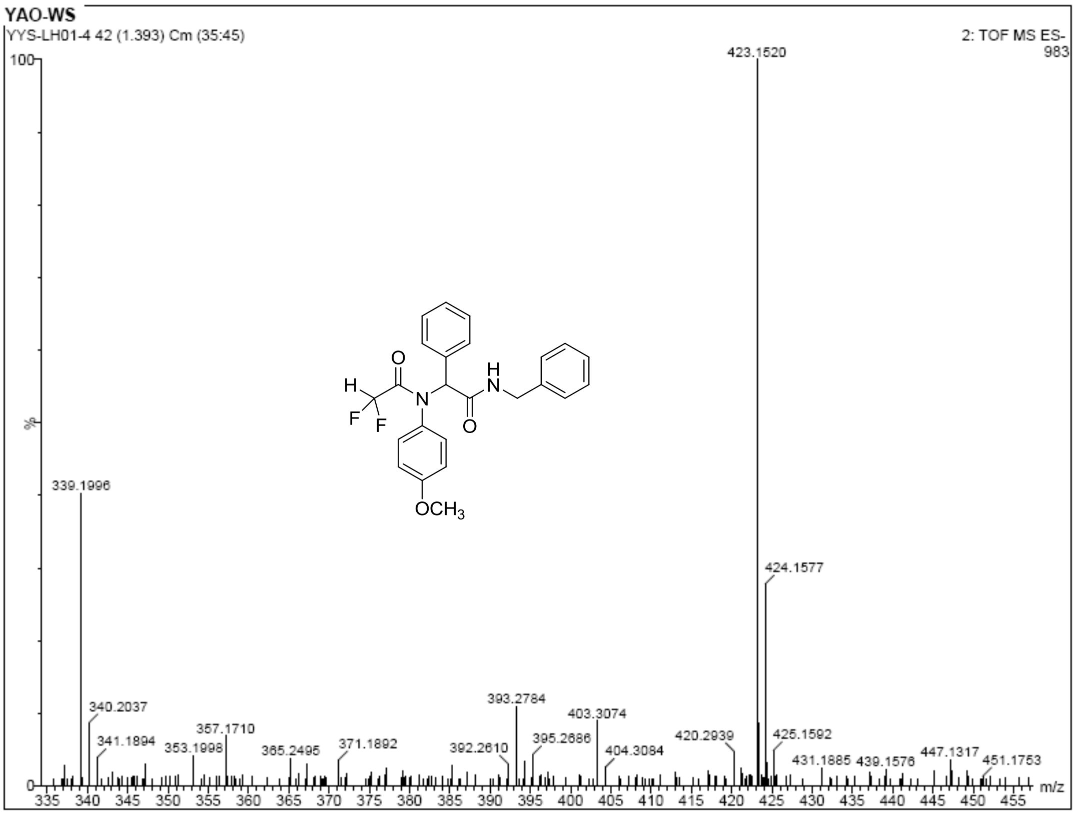

^{1}H NMR of **4e**

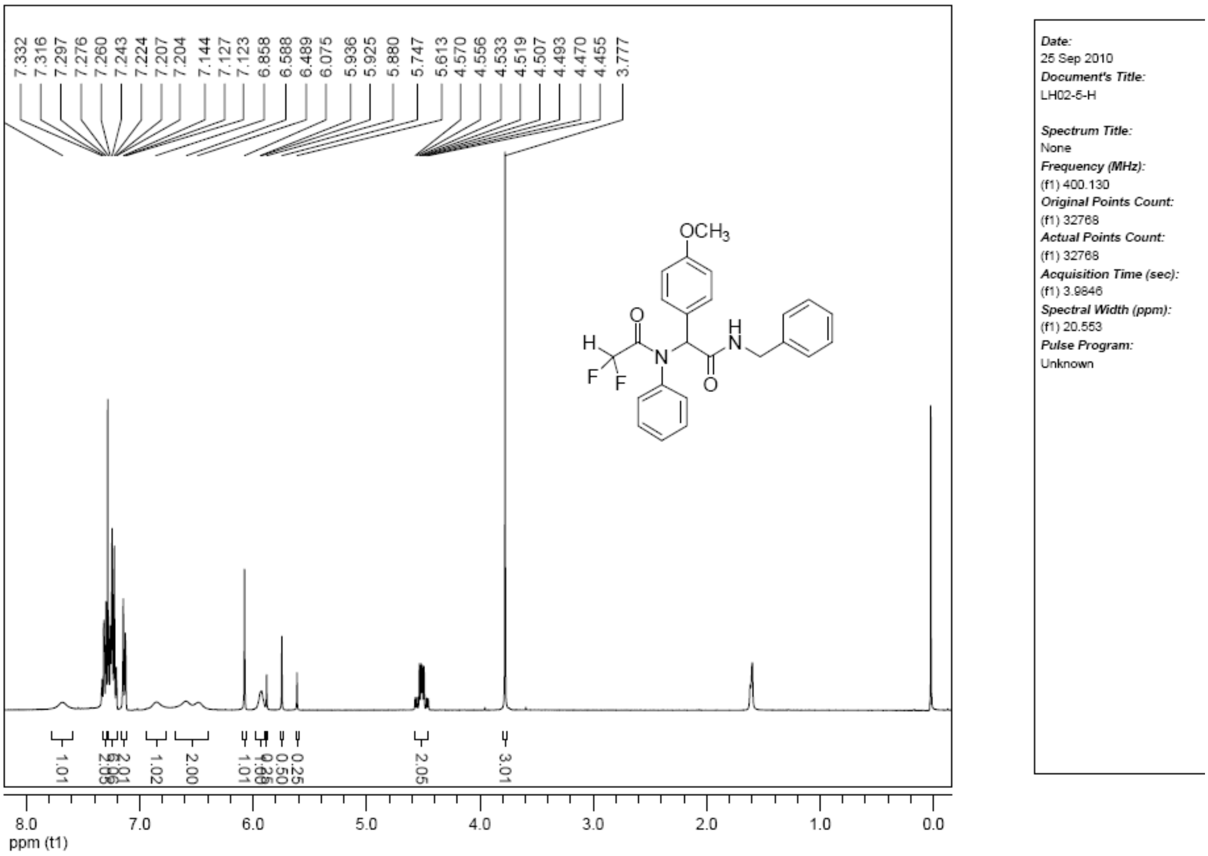

^{13}C NMR of **4e**

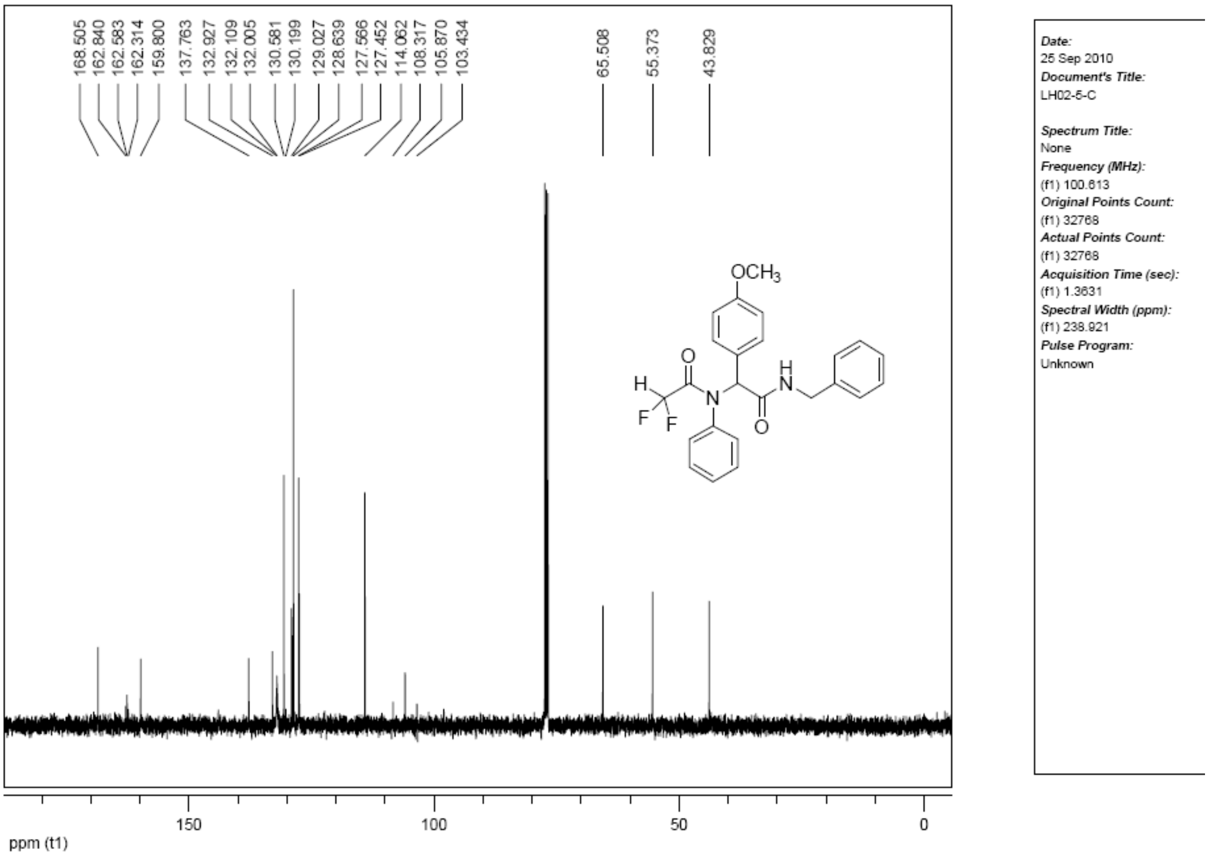

^{19}F NMR of **4e**

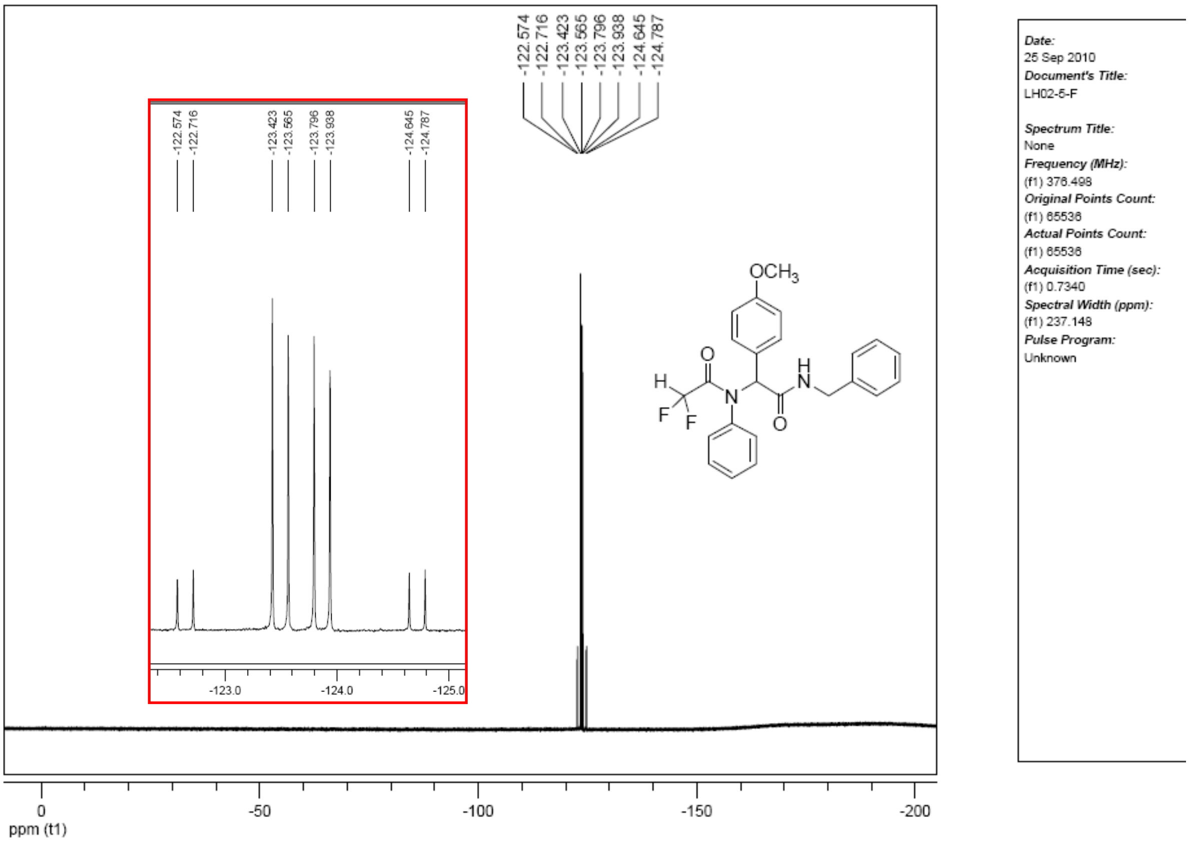

MS (ESI) of **4e**

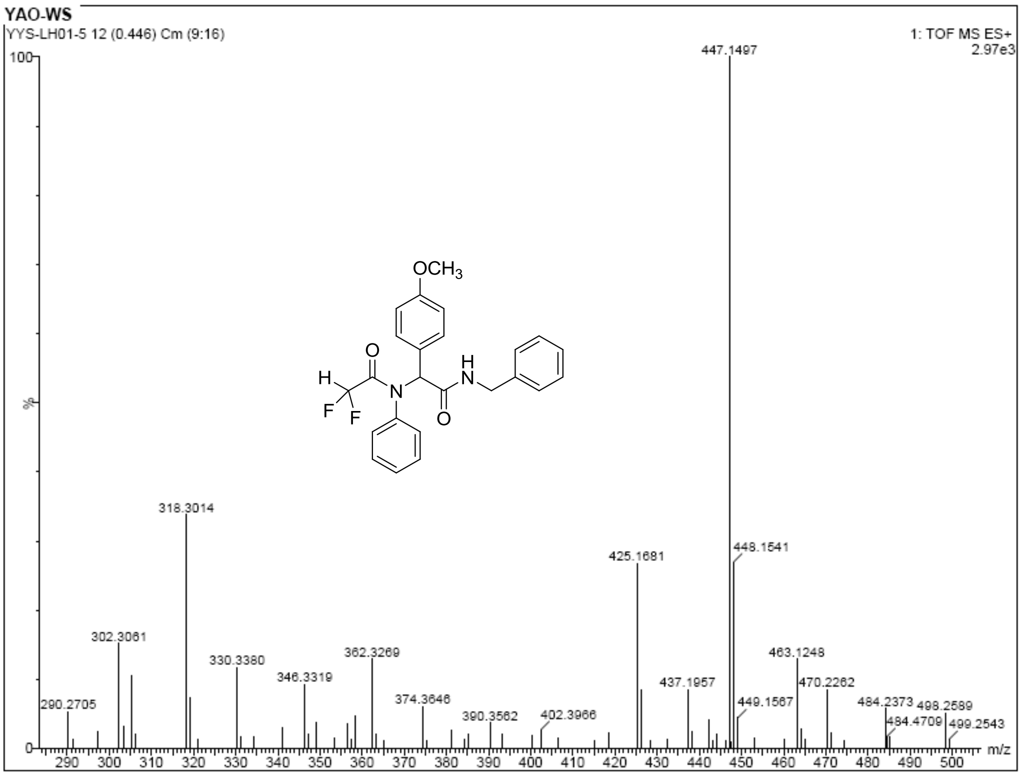

^{1}H NMR of **4f**

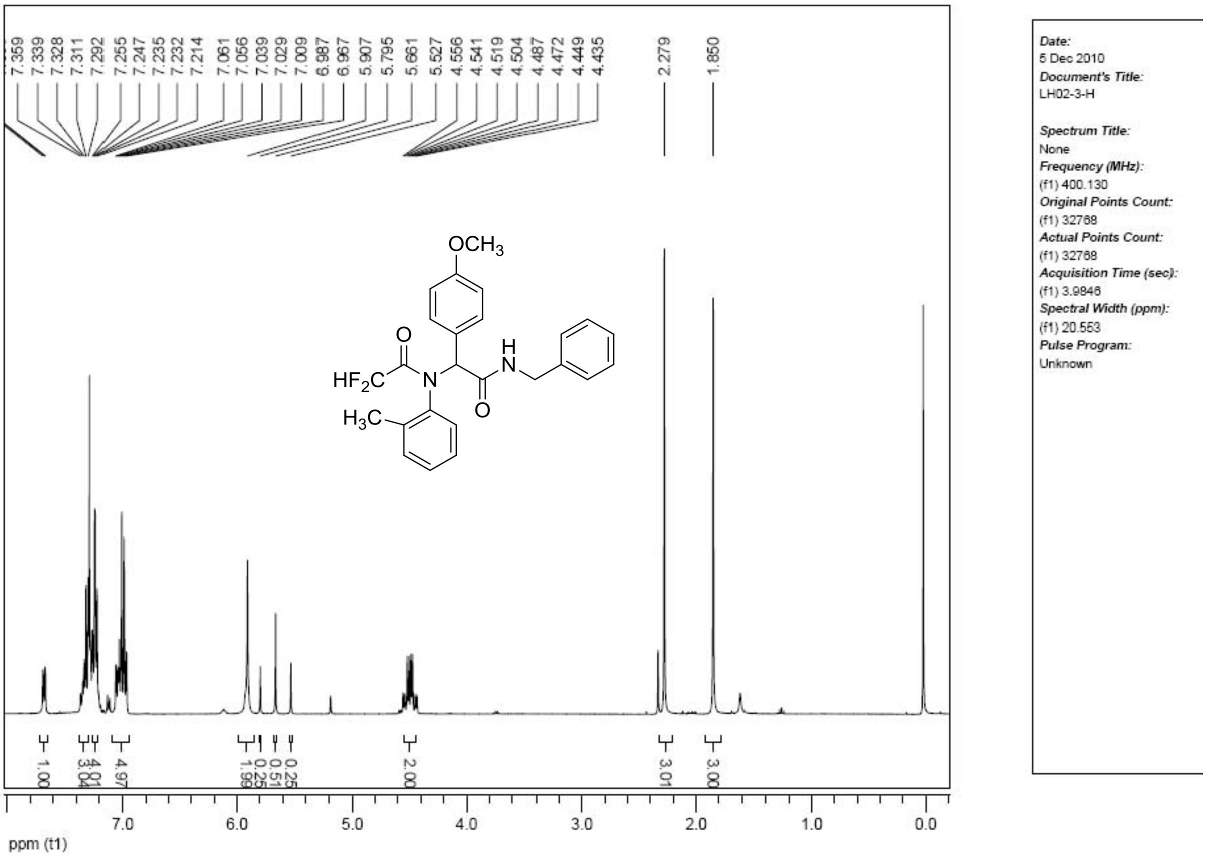

^{13}C NMR of **4f**

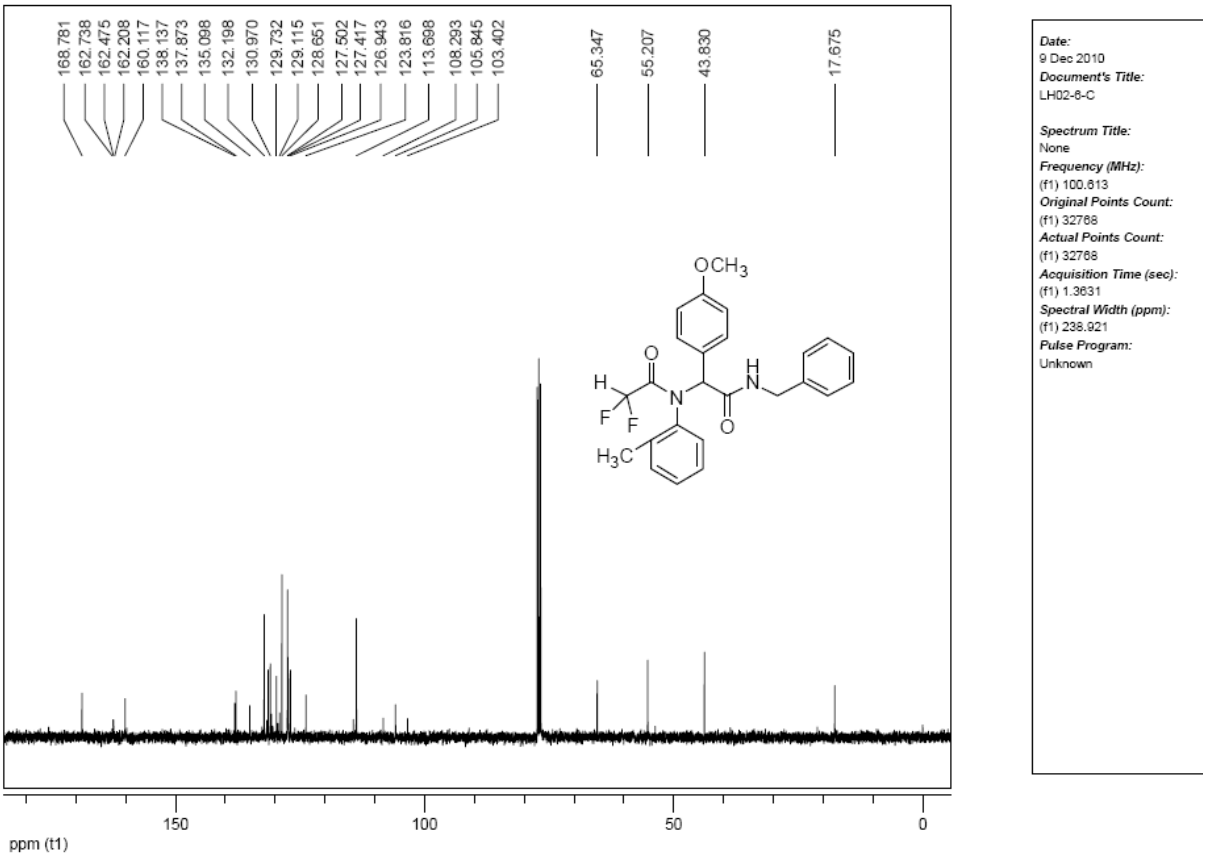

^{19}F NMR of **4f**

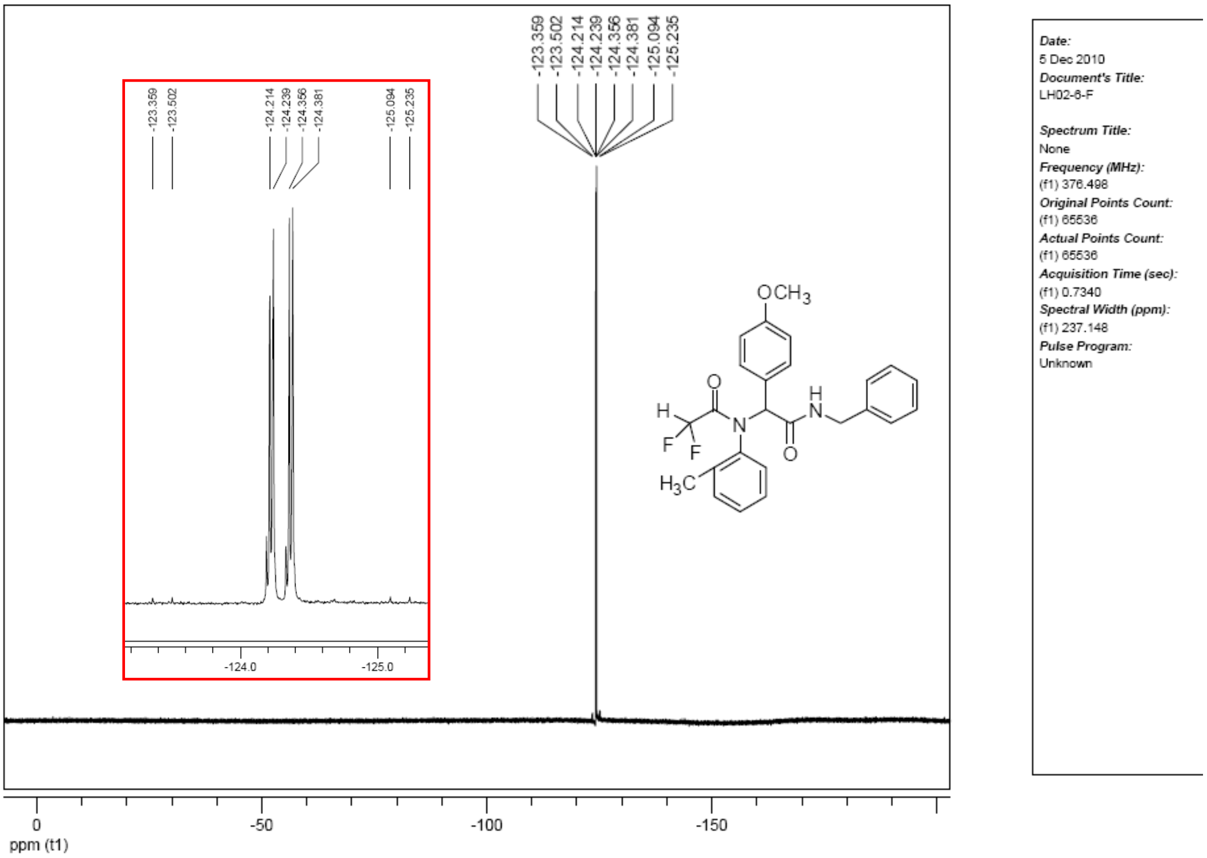

MS (ESI) of **4f**

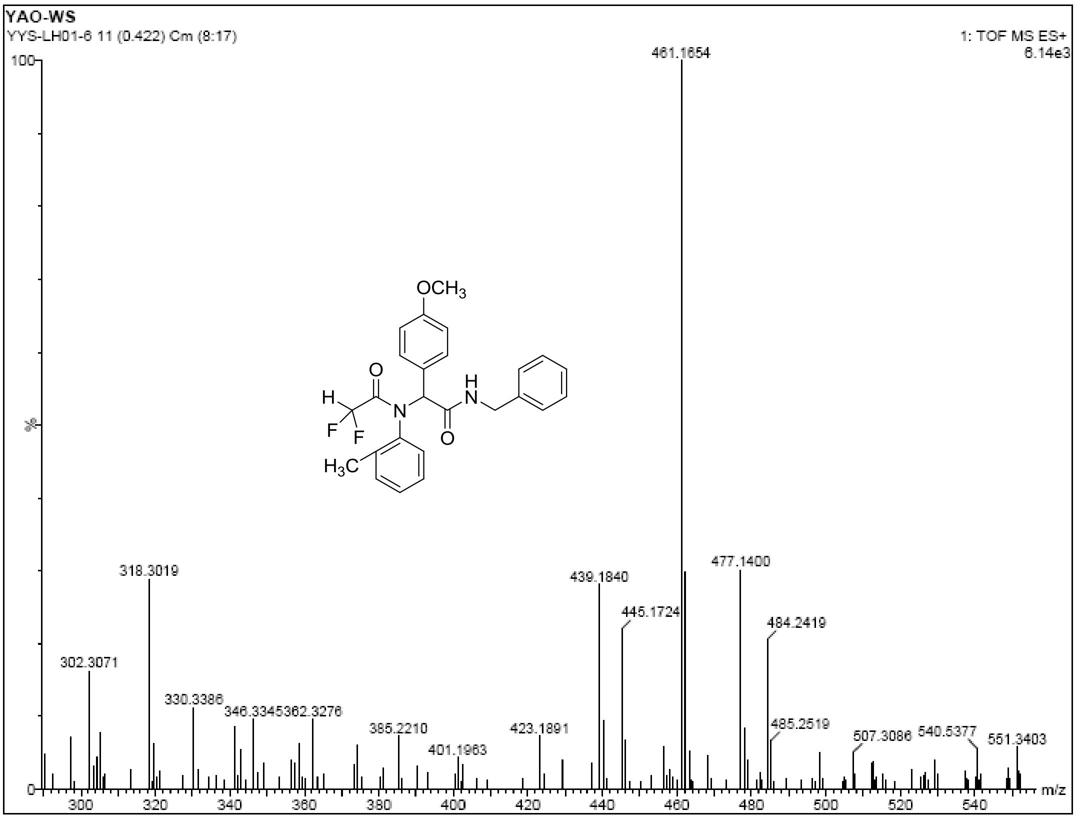

^{1}H NMR of **4g**

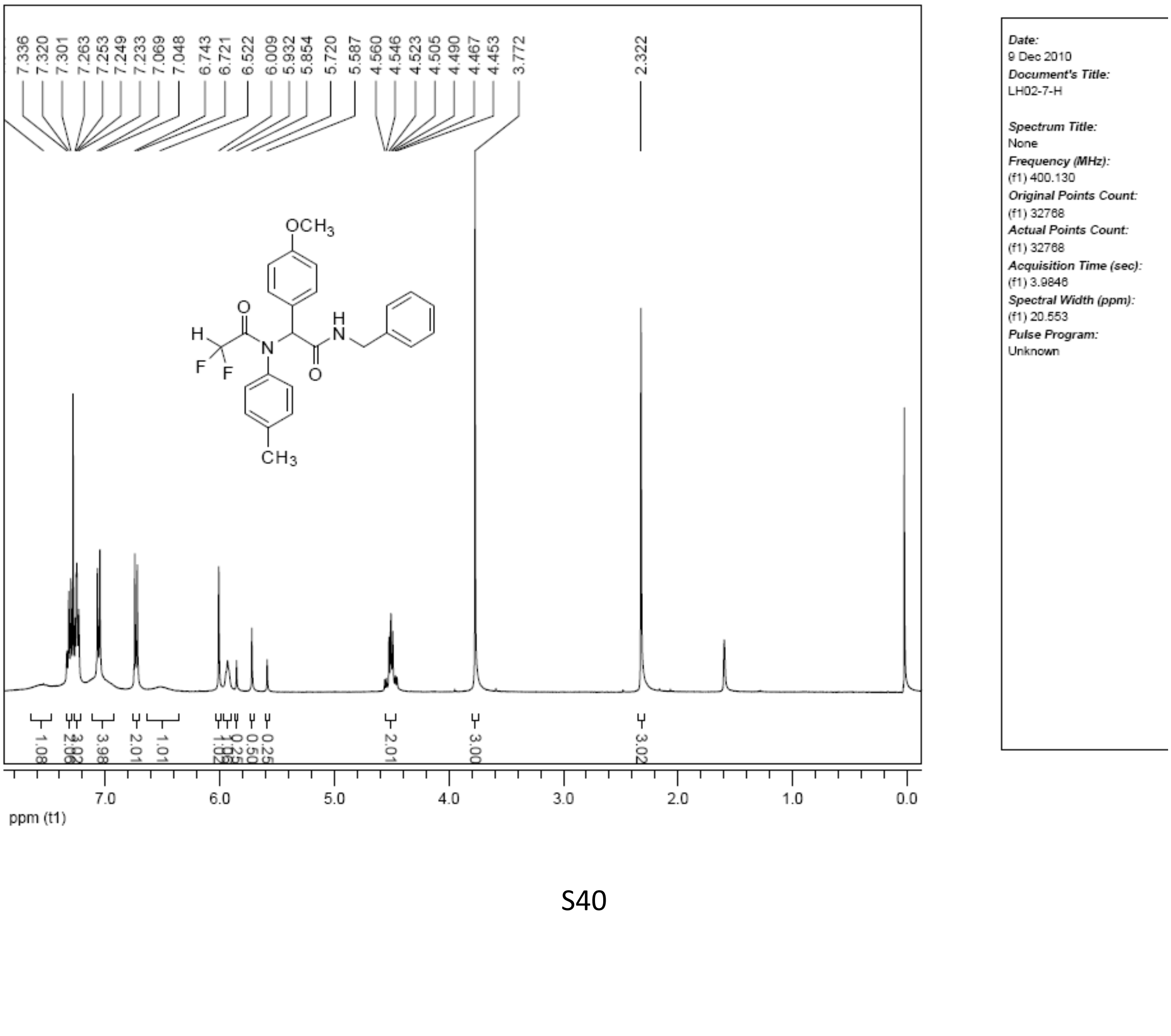

^{13}C NMR of **4g**

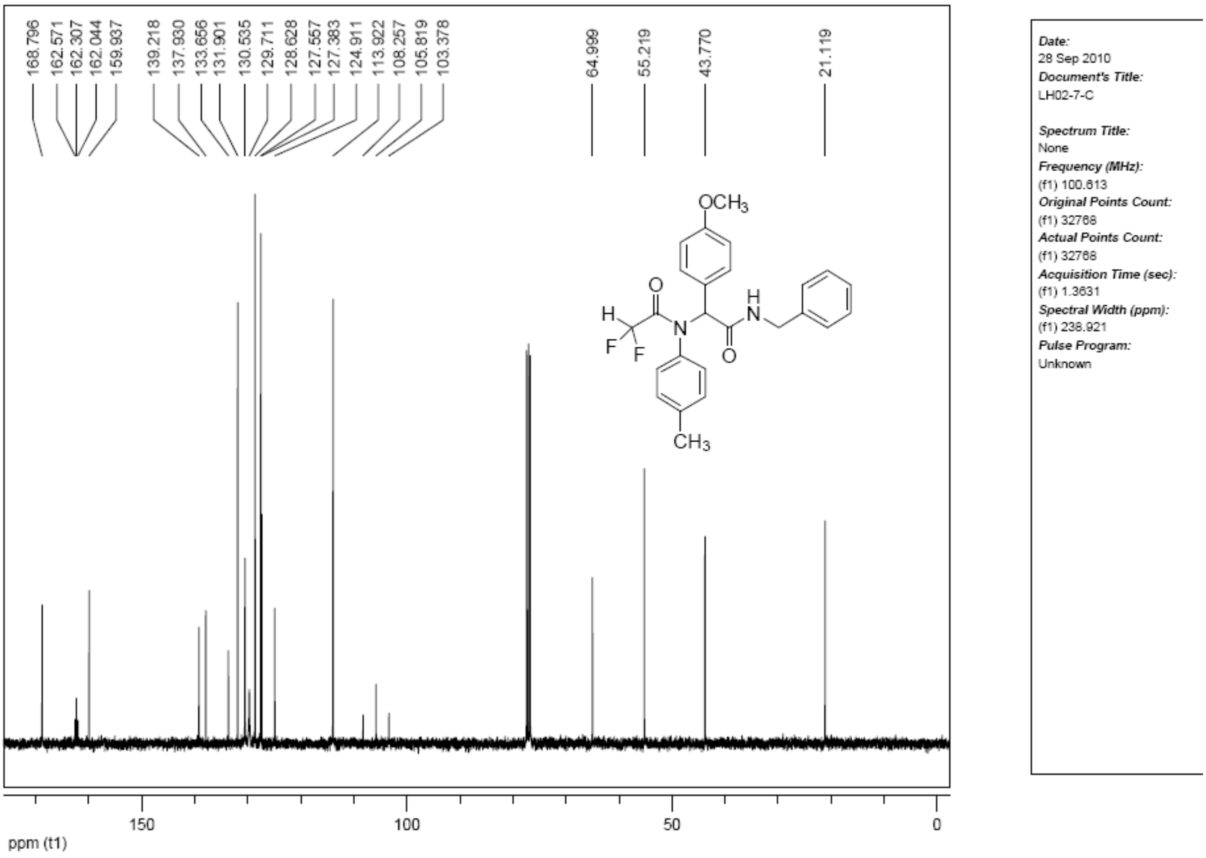

^{19}F NMR of **4g**

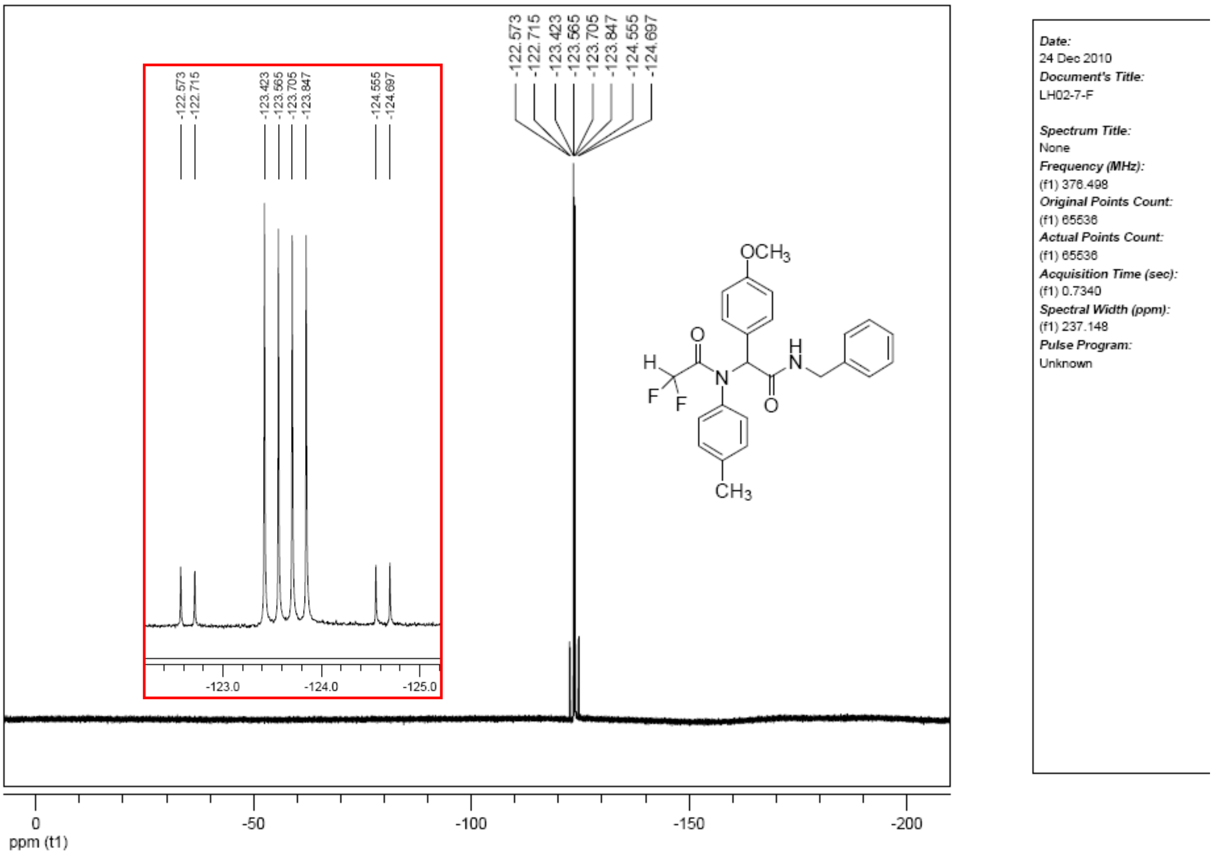

MS (ESI) of **4g**

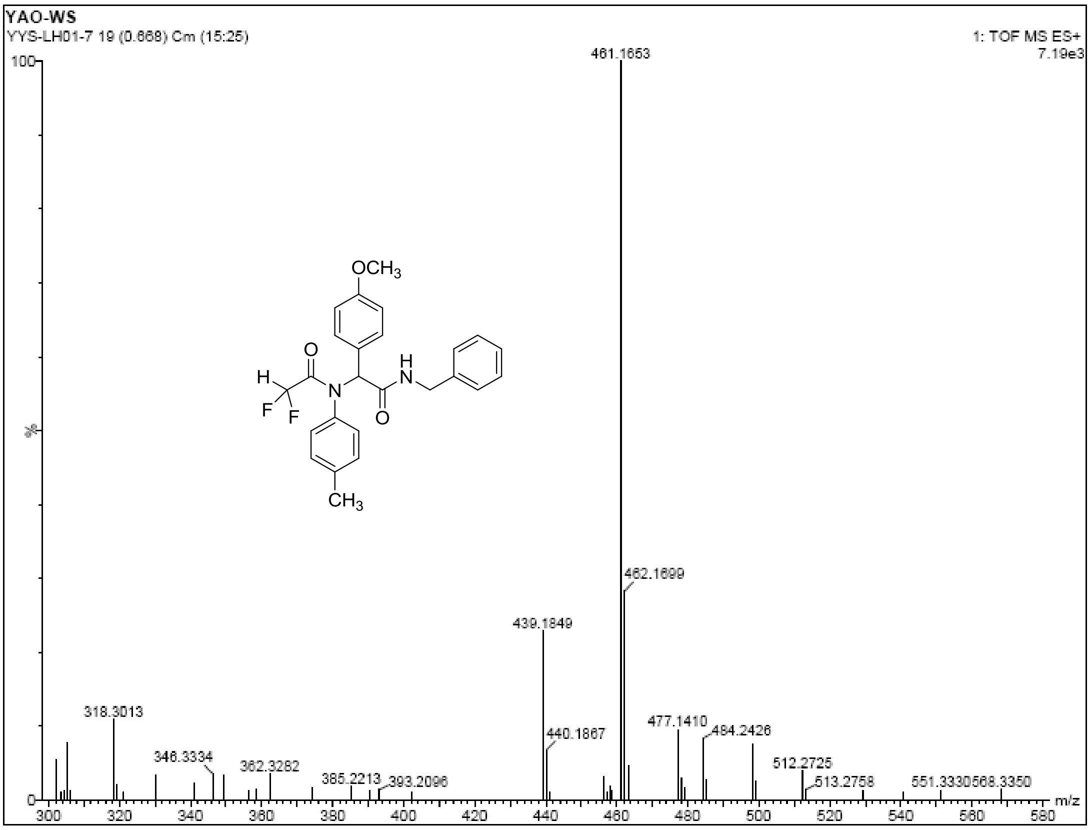

^{1}H NMR of **4h**

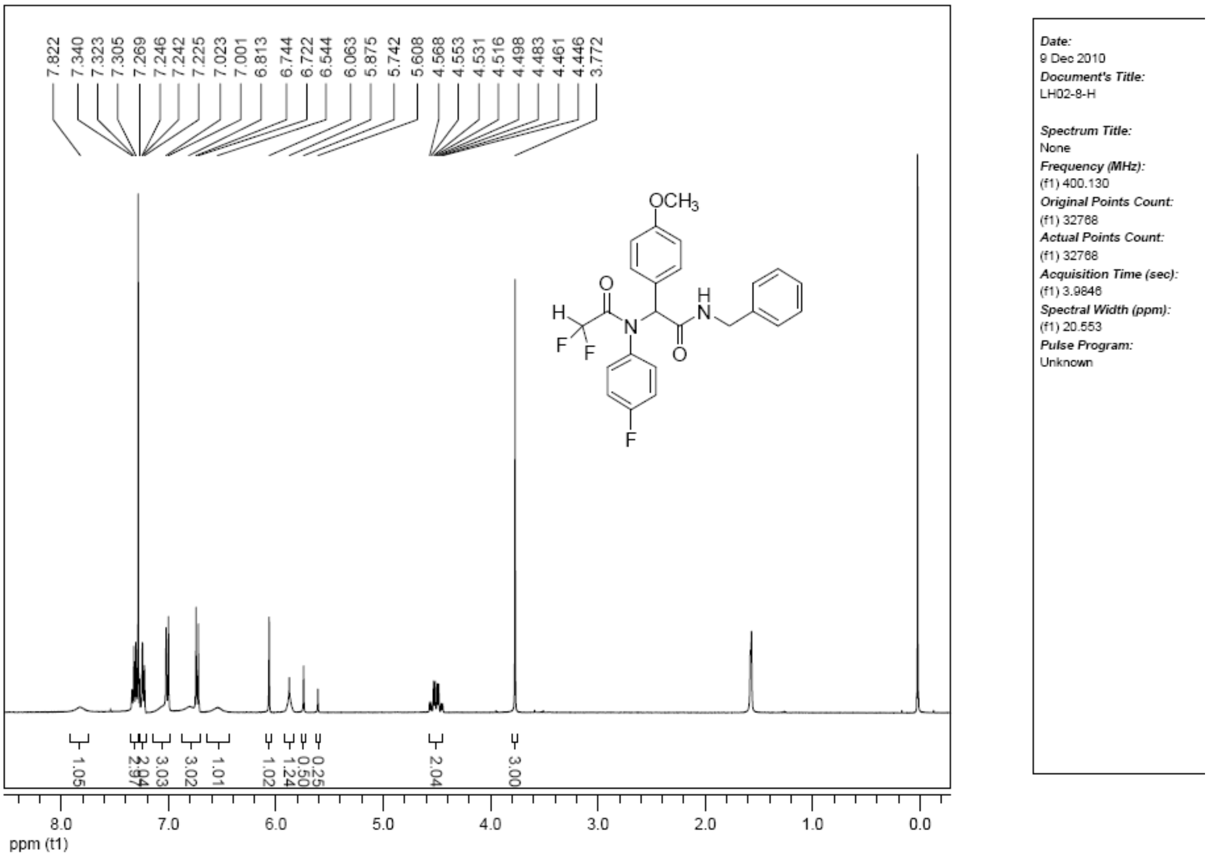

^{13}C NMR of **4h**

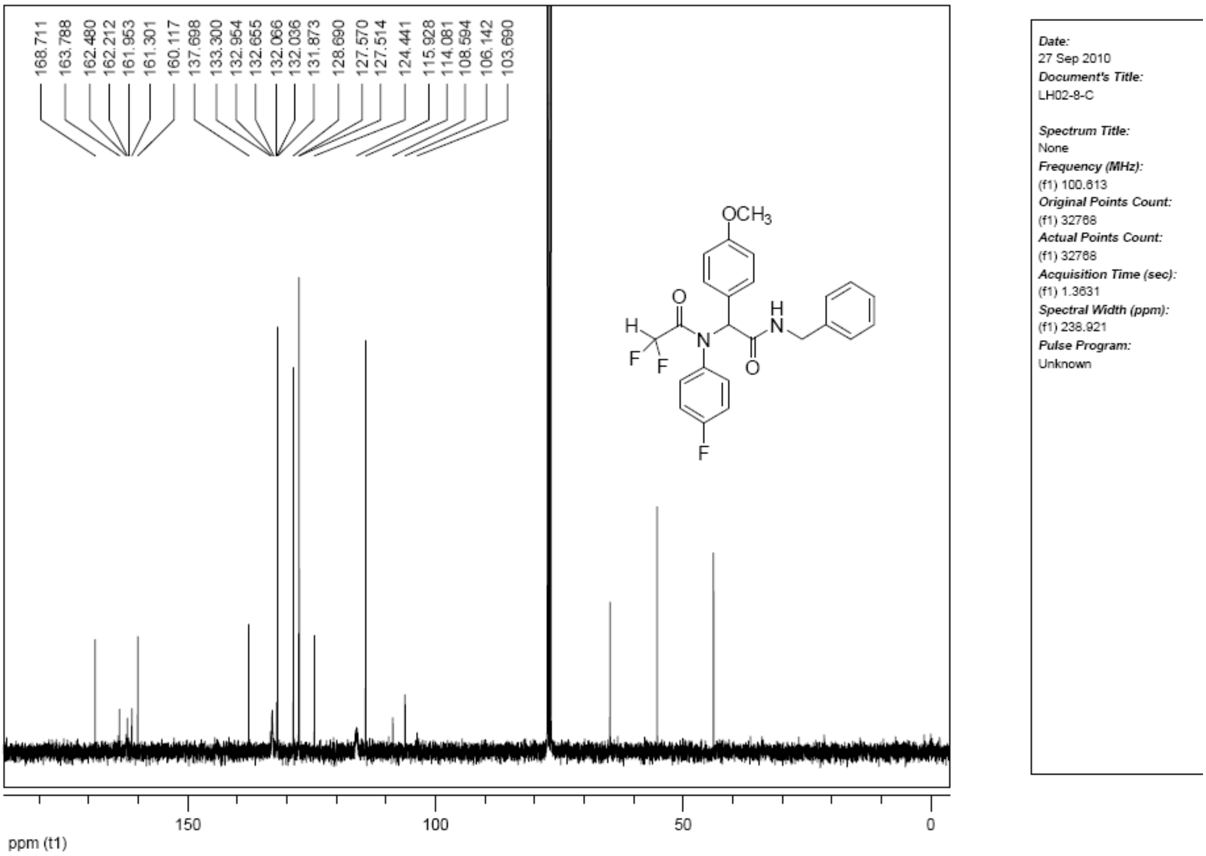

^{19}F NMR of **4h**

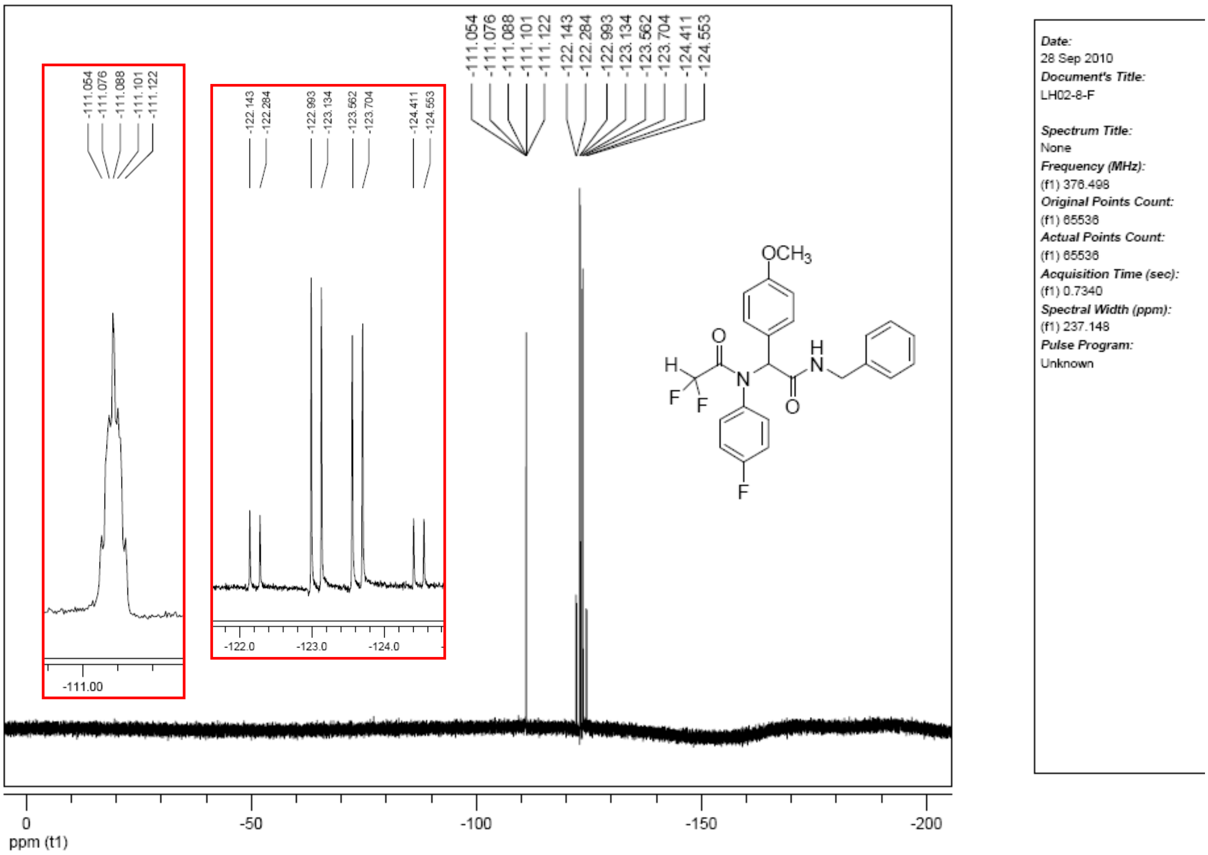

MS (ESI) of **4h**

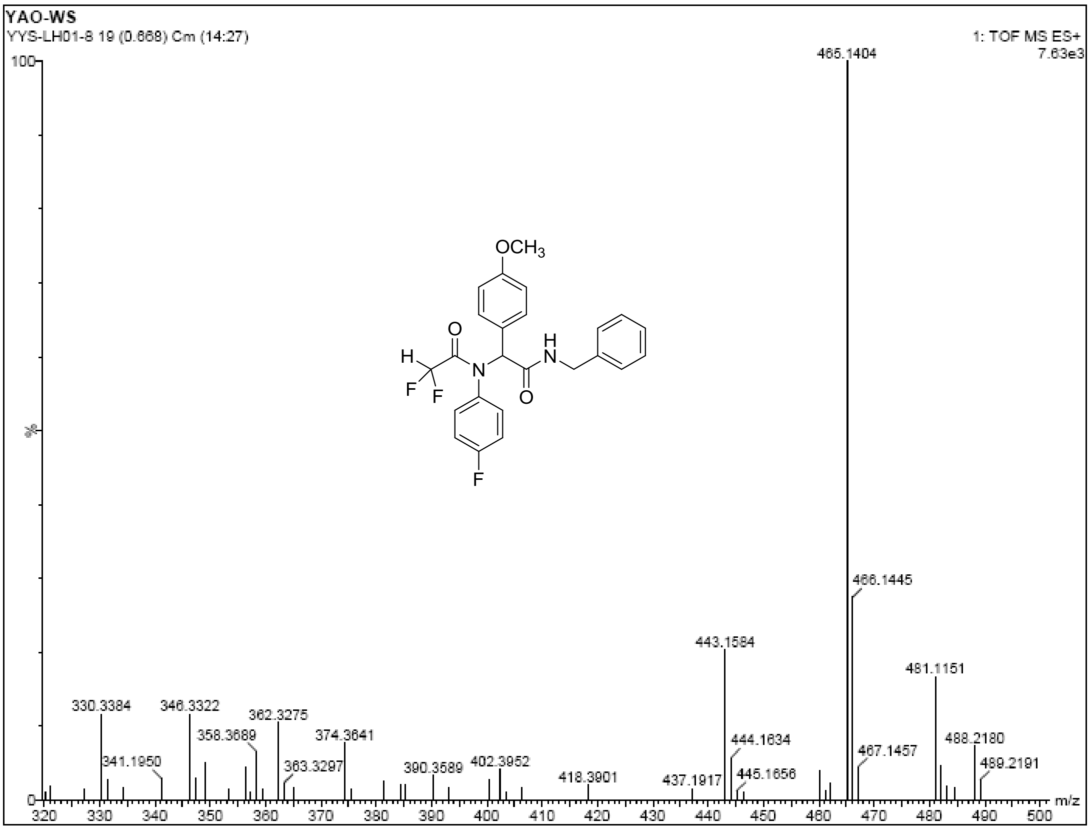

^{1}H NMR of **4i**

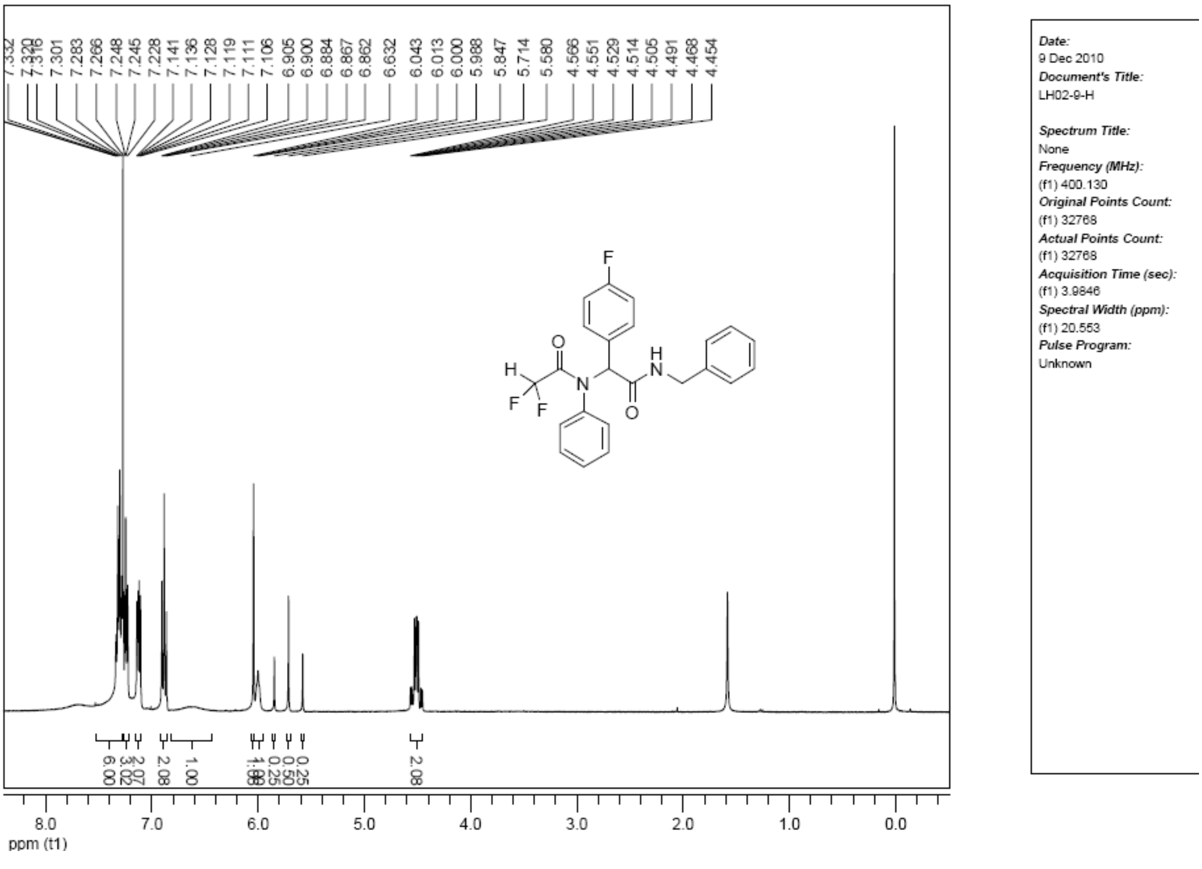

^{13}C NMR of **4i**

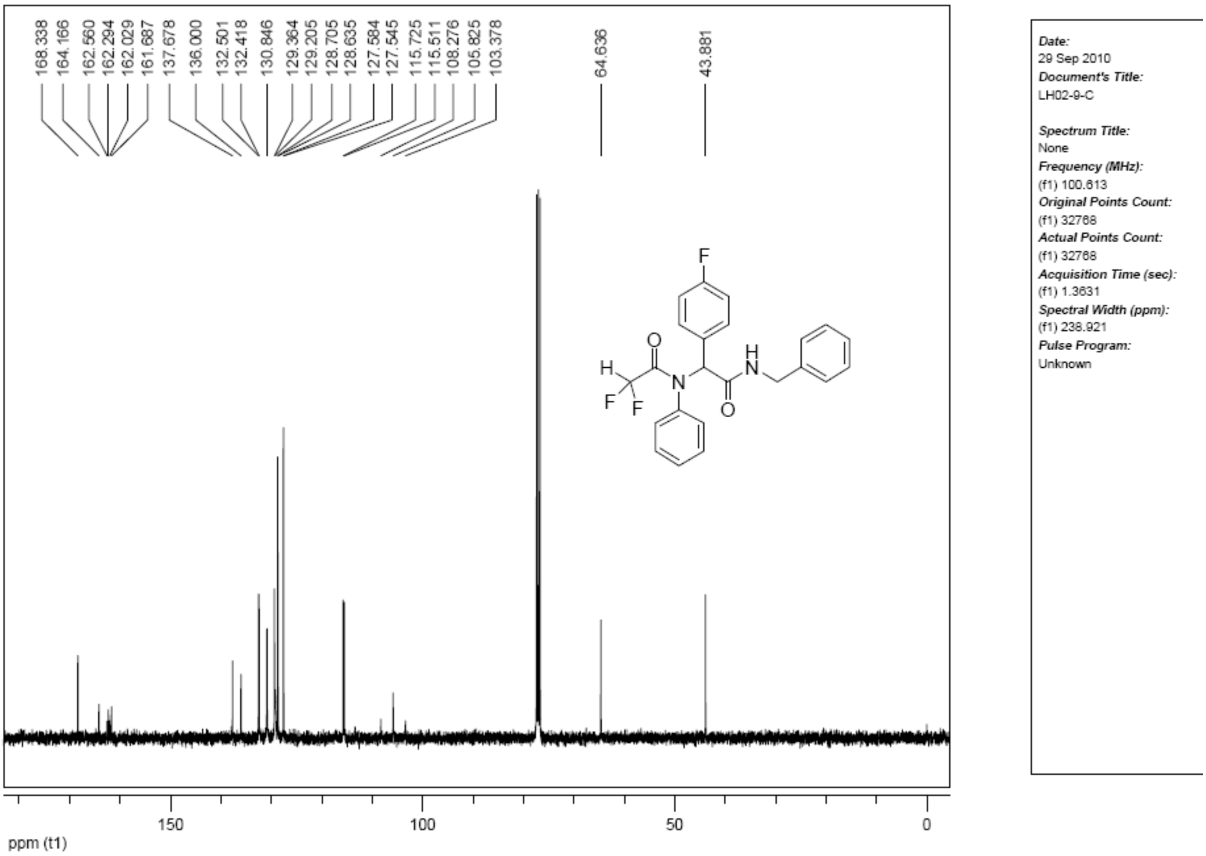

^{19}F NMR of **4i**

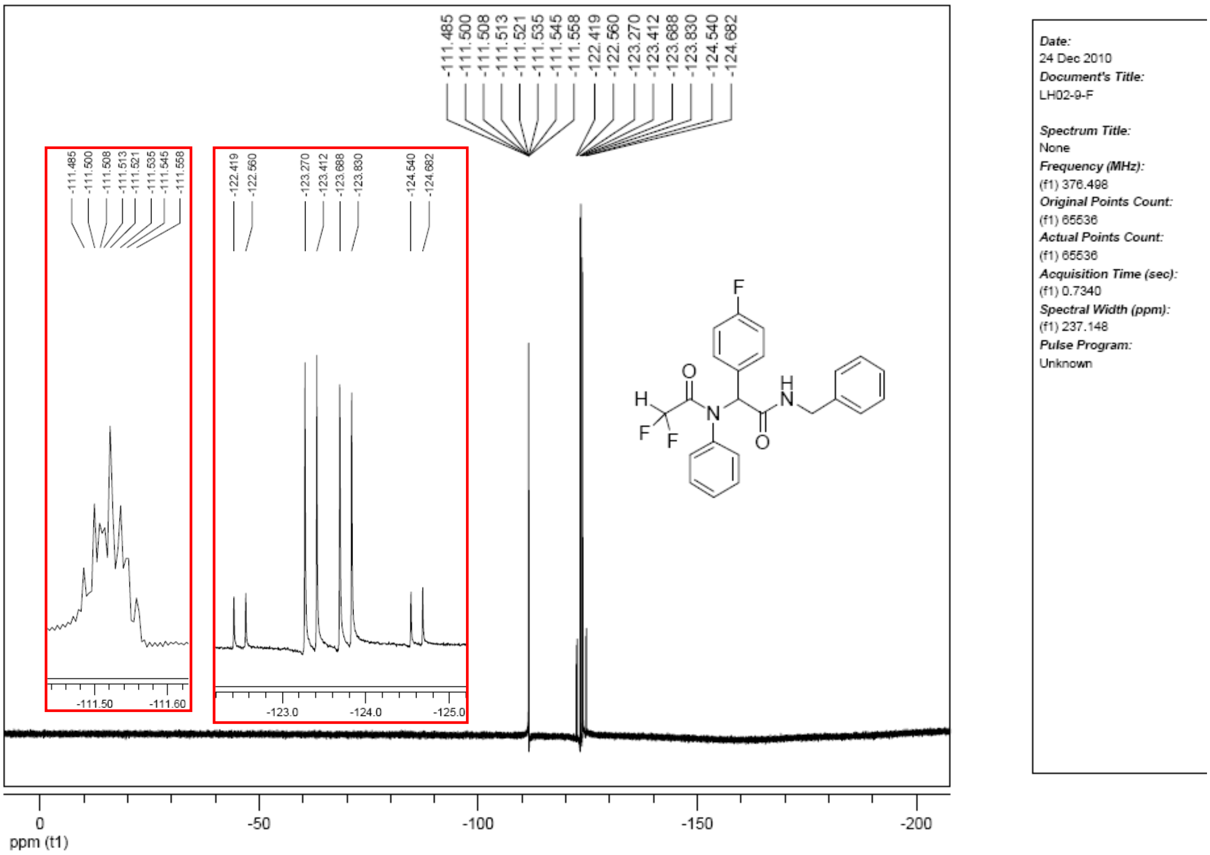

MS (ESI) of **4i**

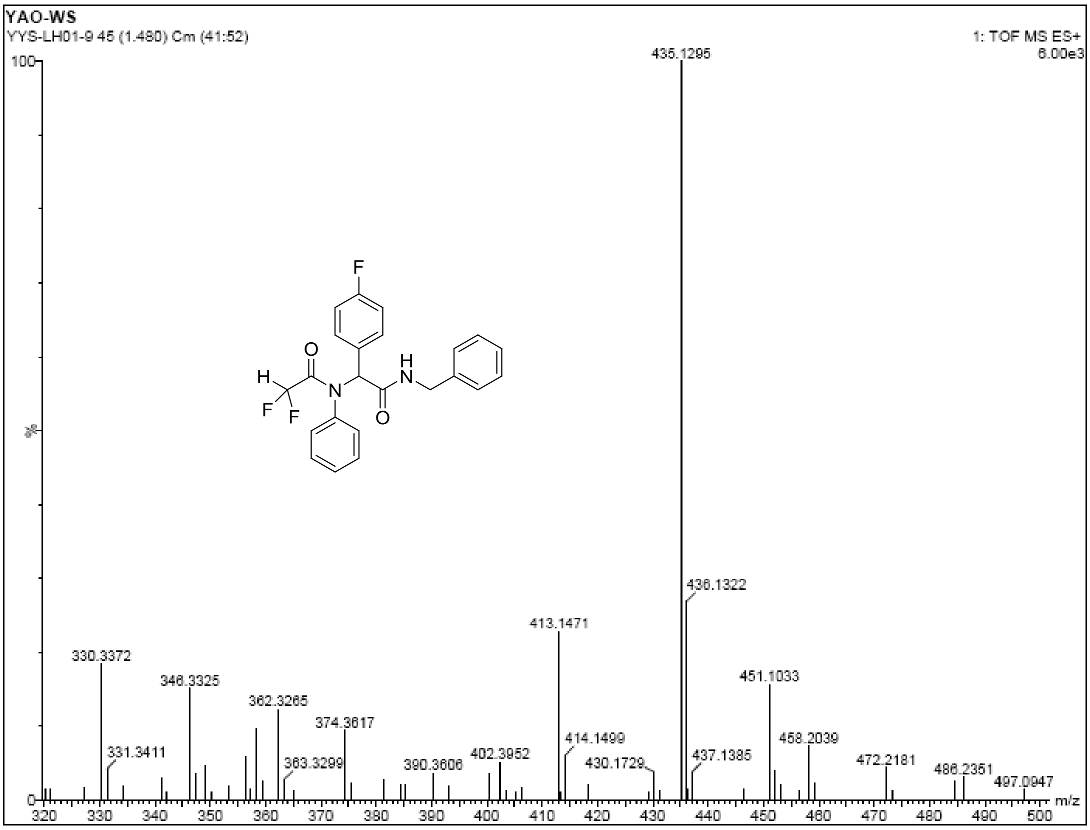

^{1}H NMR of **4j**

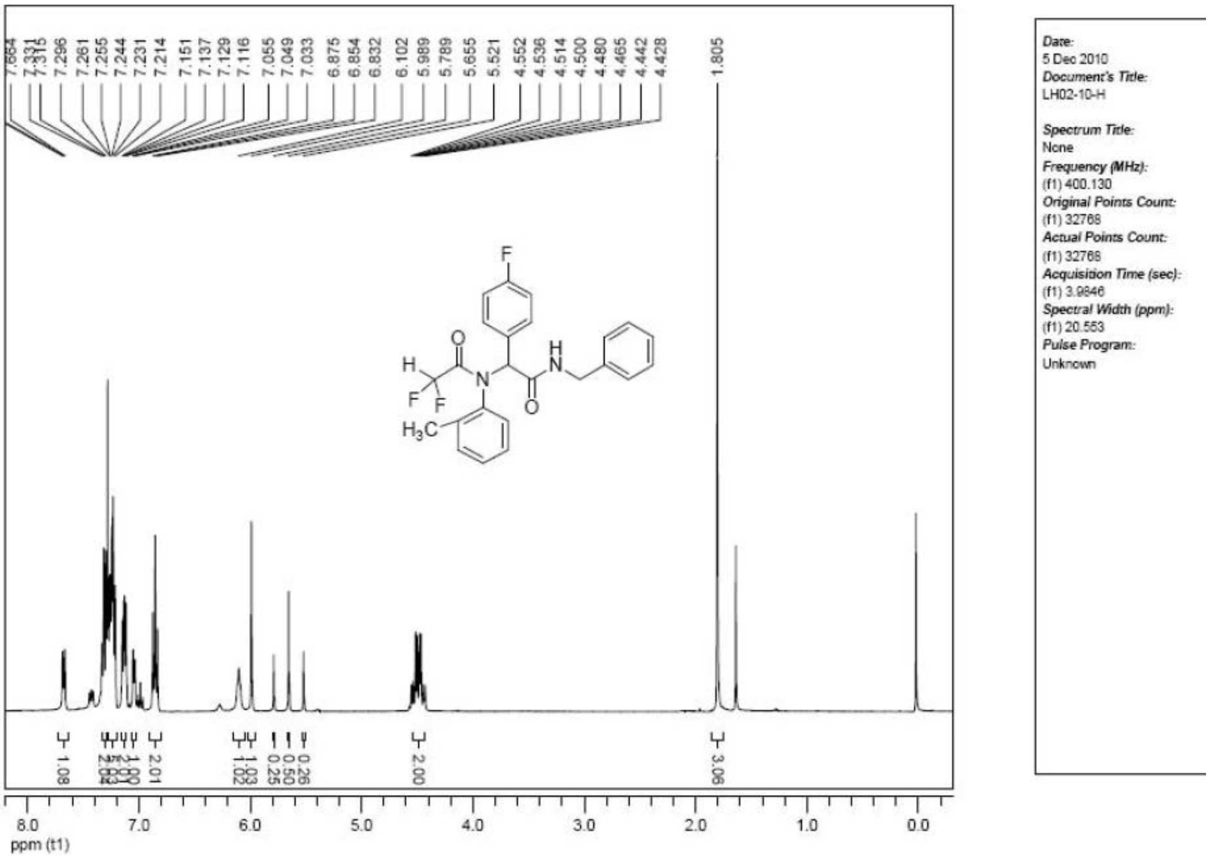

^{13}C NMR of **4j**

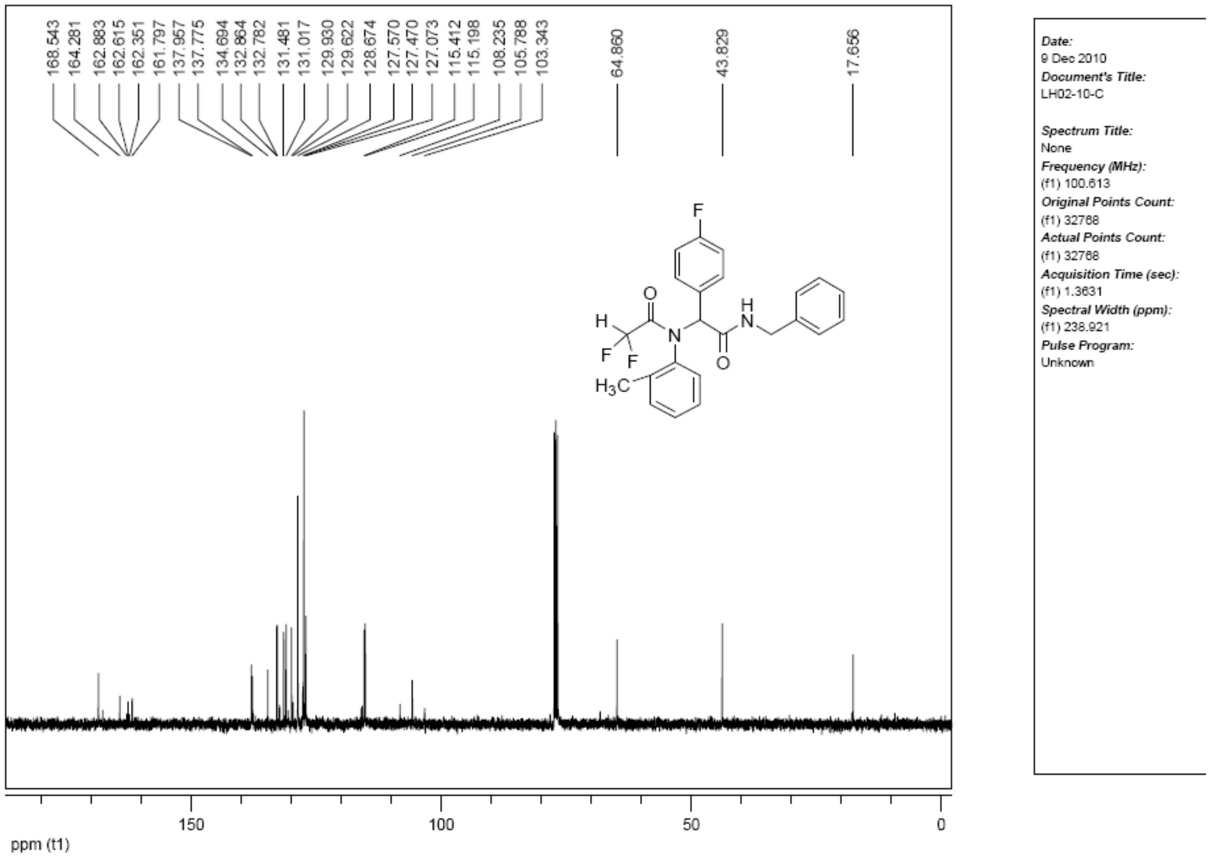

^{19}F NMR of **4j**

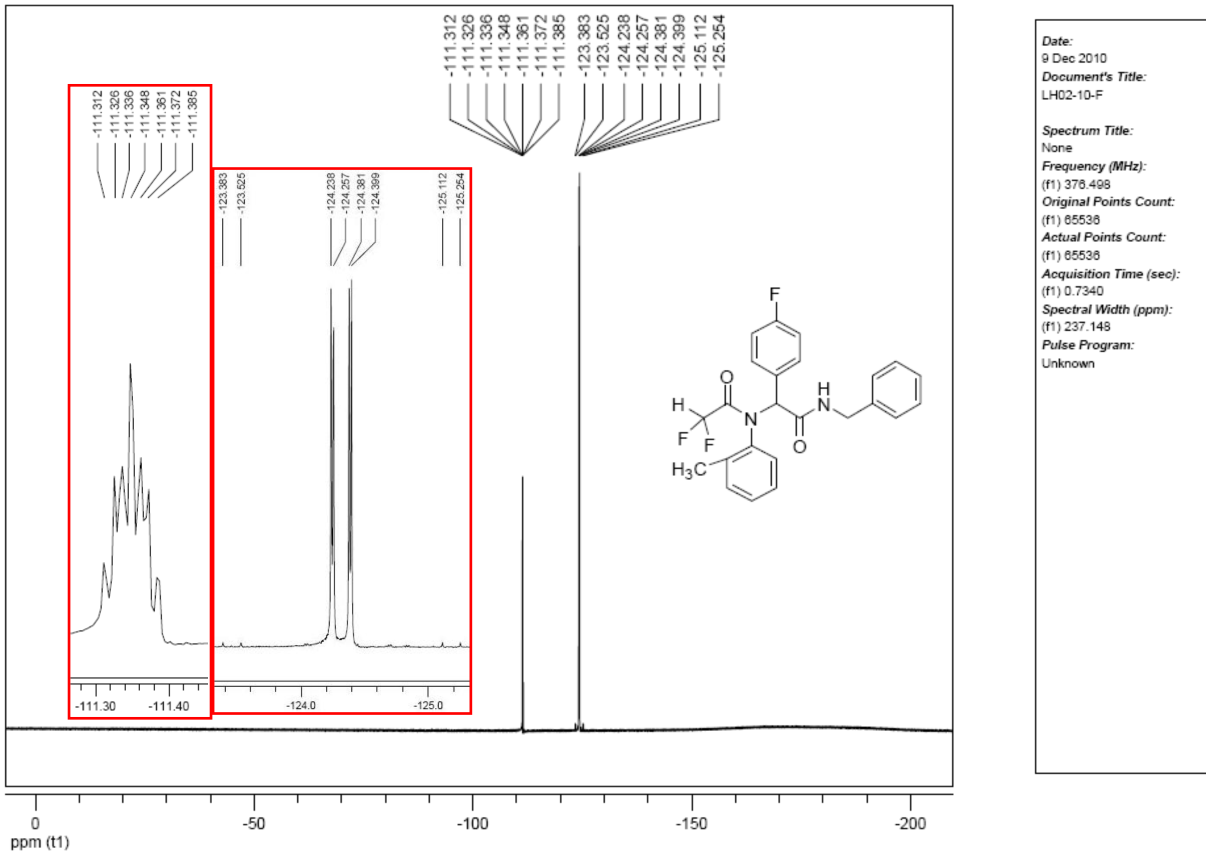

MS (ESI) of **4j**

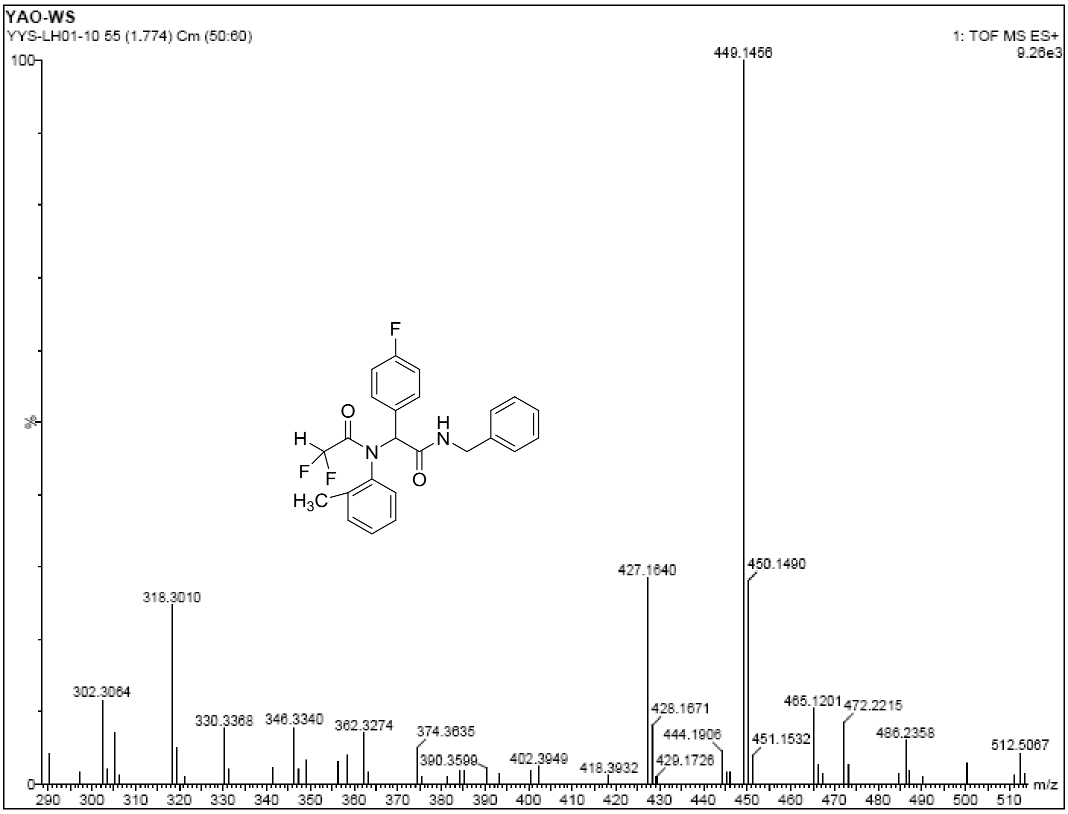

^{1}H NMR of **4k**

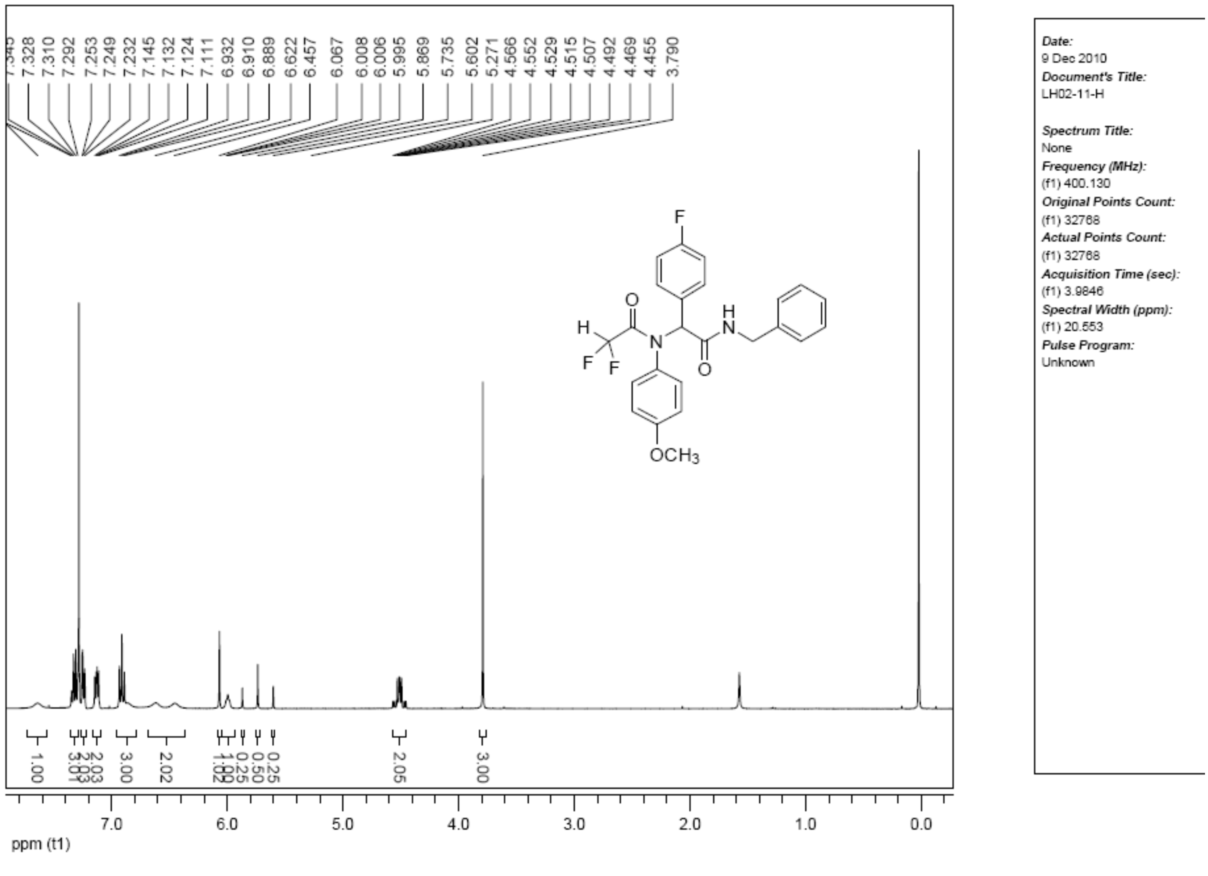

^{13}C NMR of **4k**

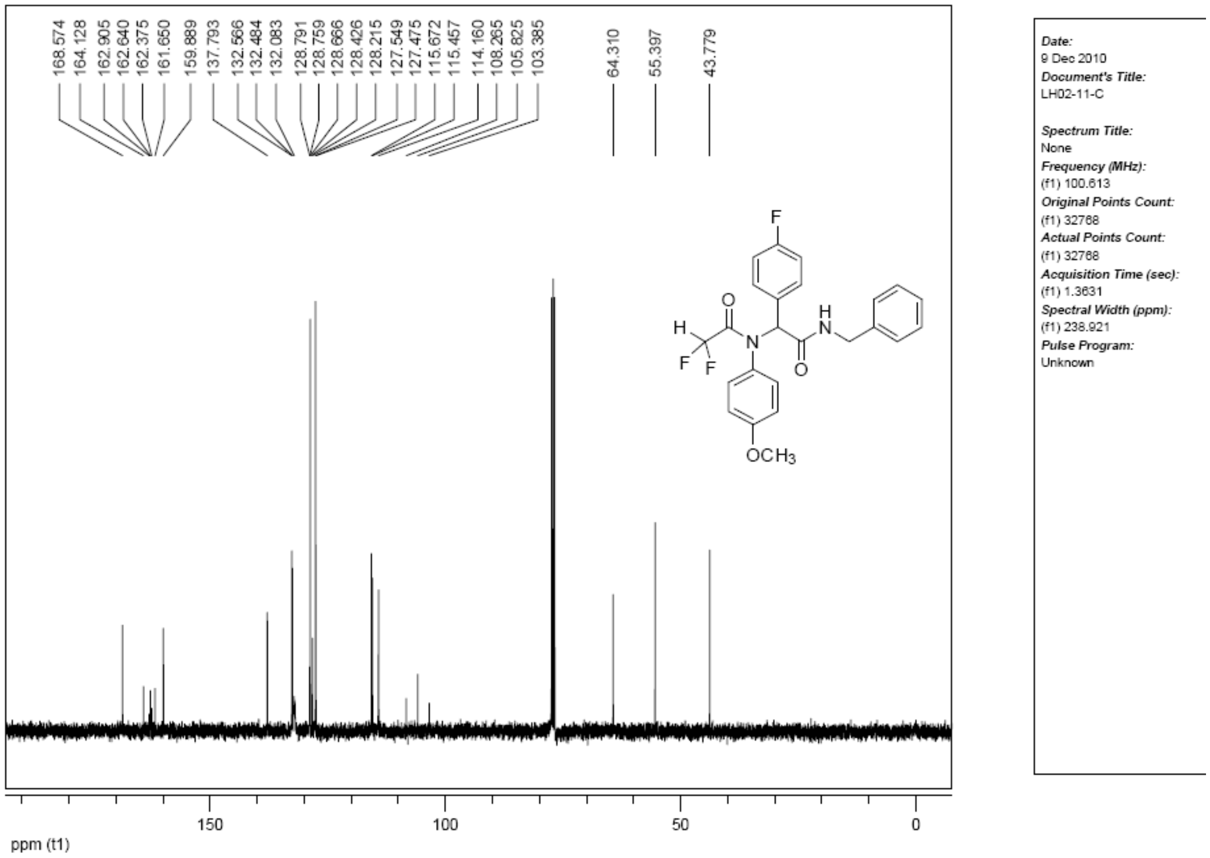

^{19}F NMR of **4k**

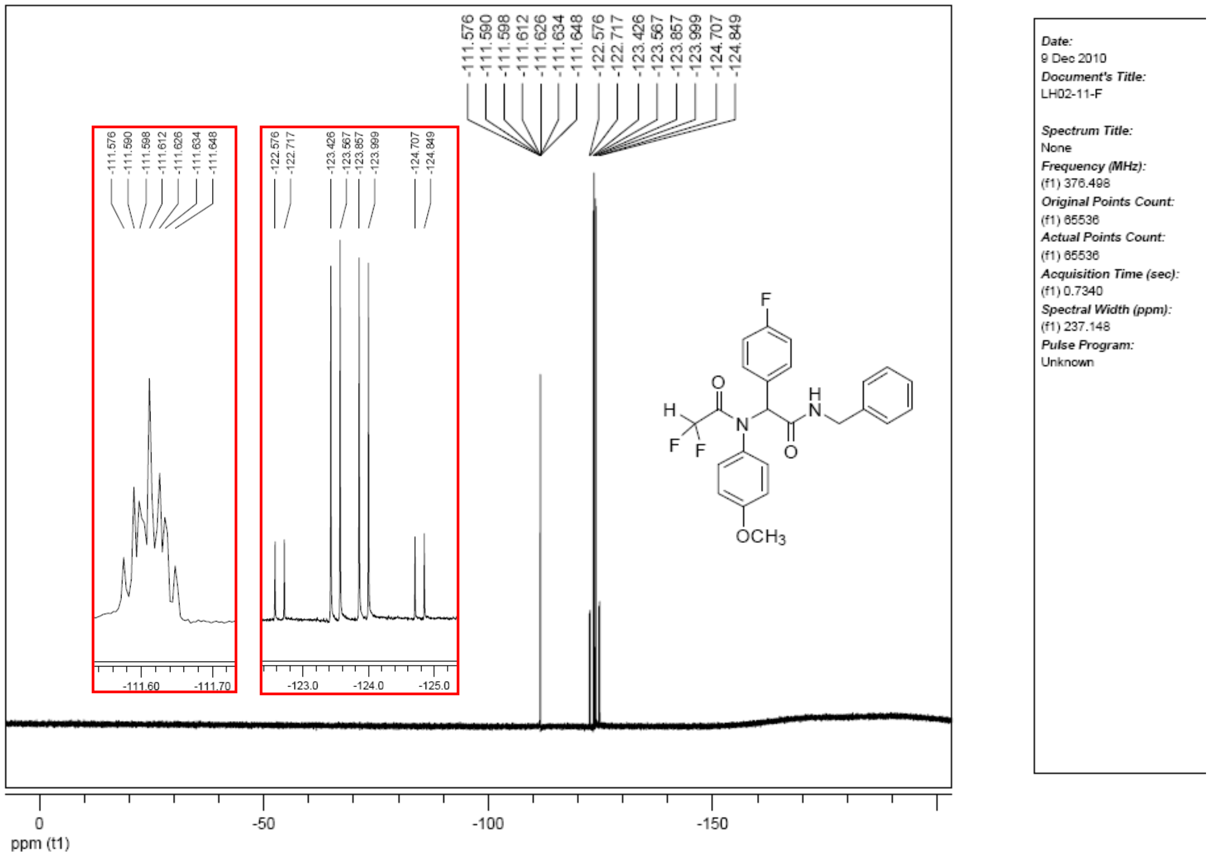

MS (ESI) of **4k**

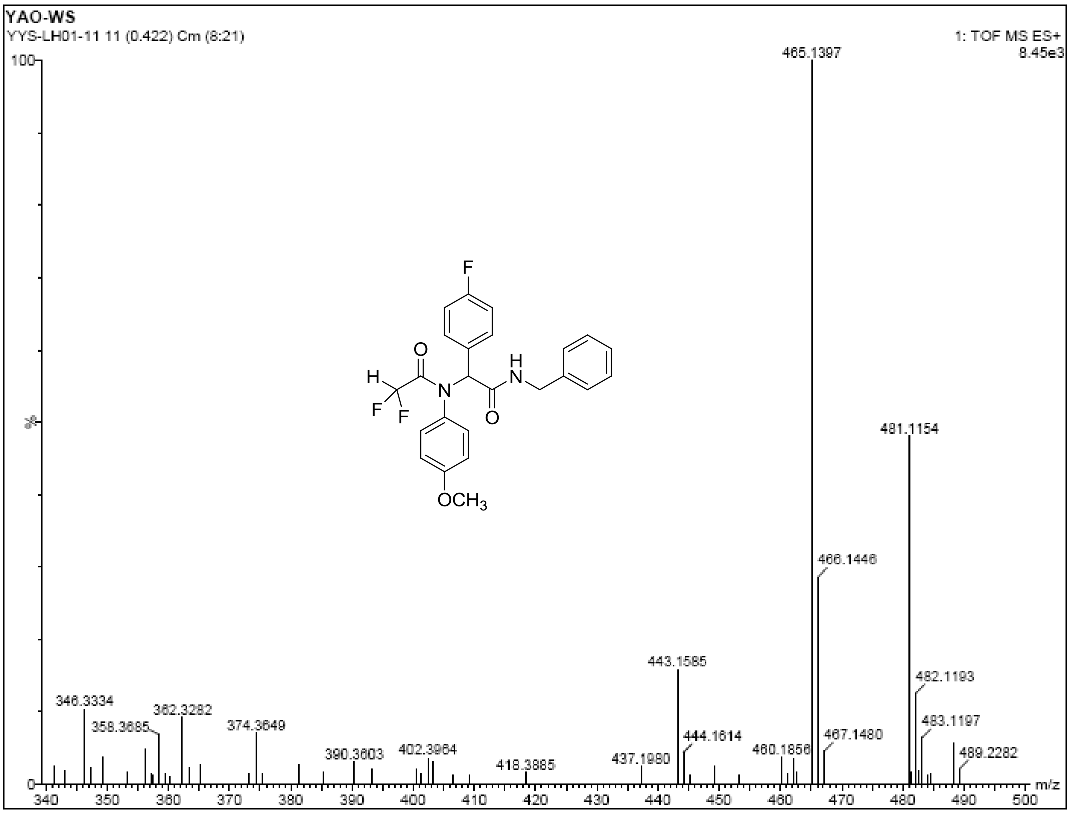

^{1}H NMR of **4l**

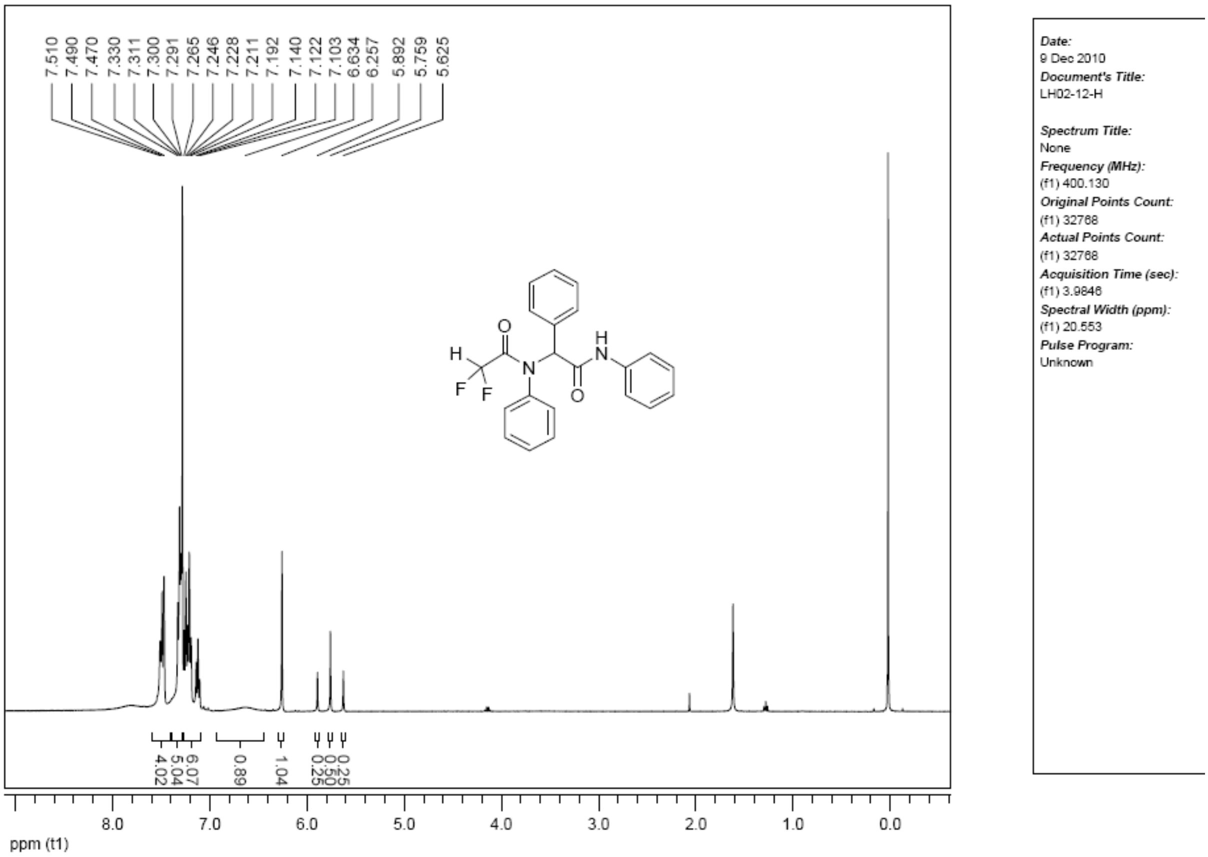

^{13}C NMR of **4l**

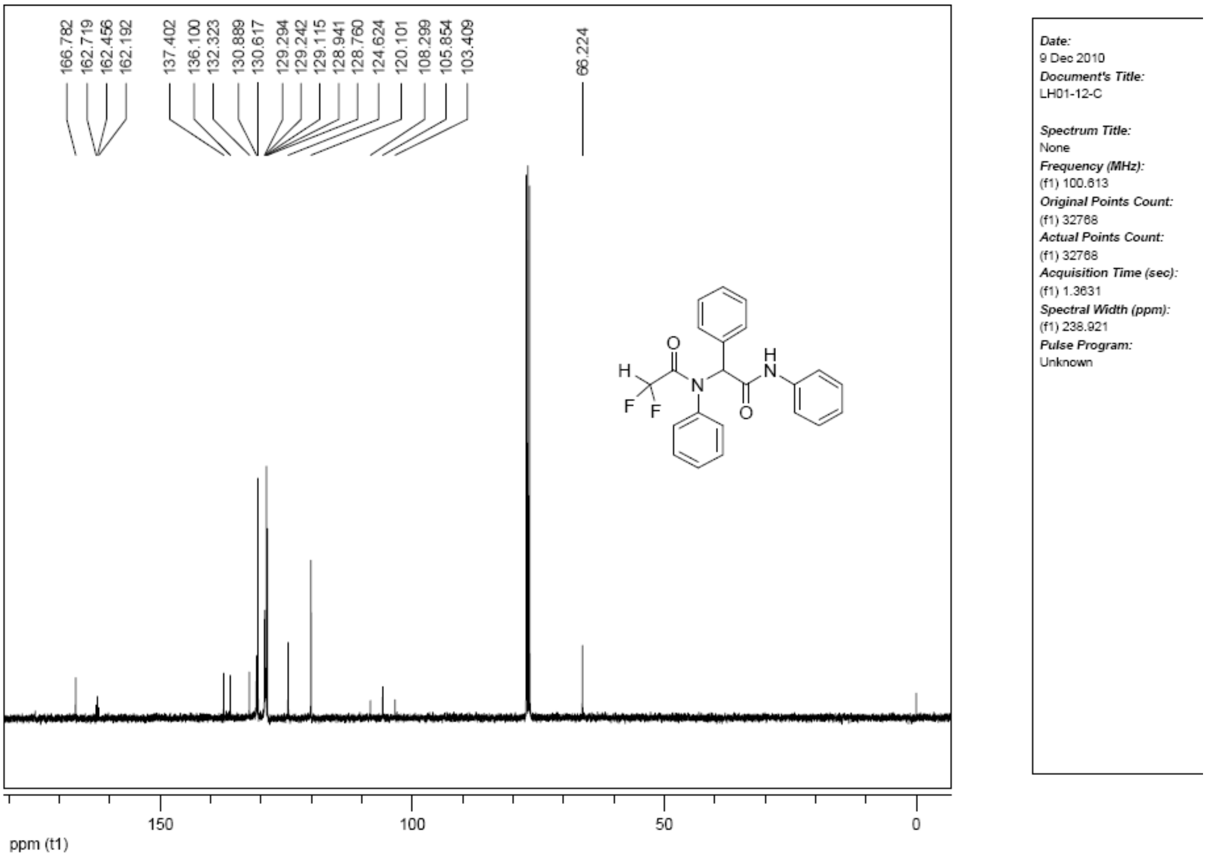

^{19}F NMR of **4l**

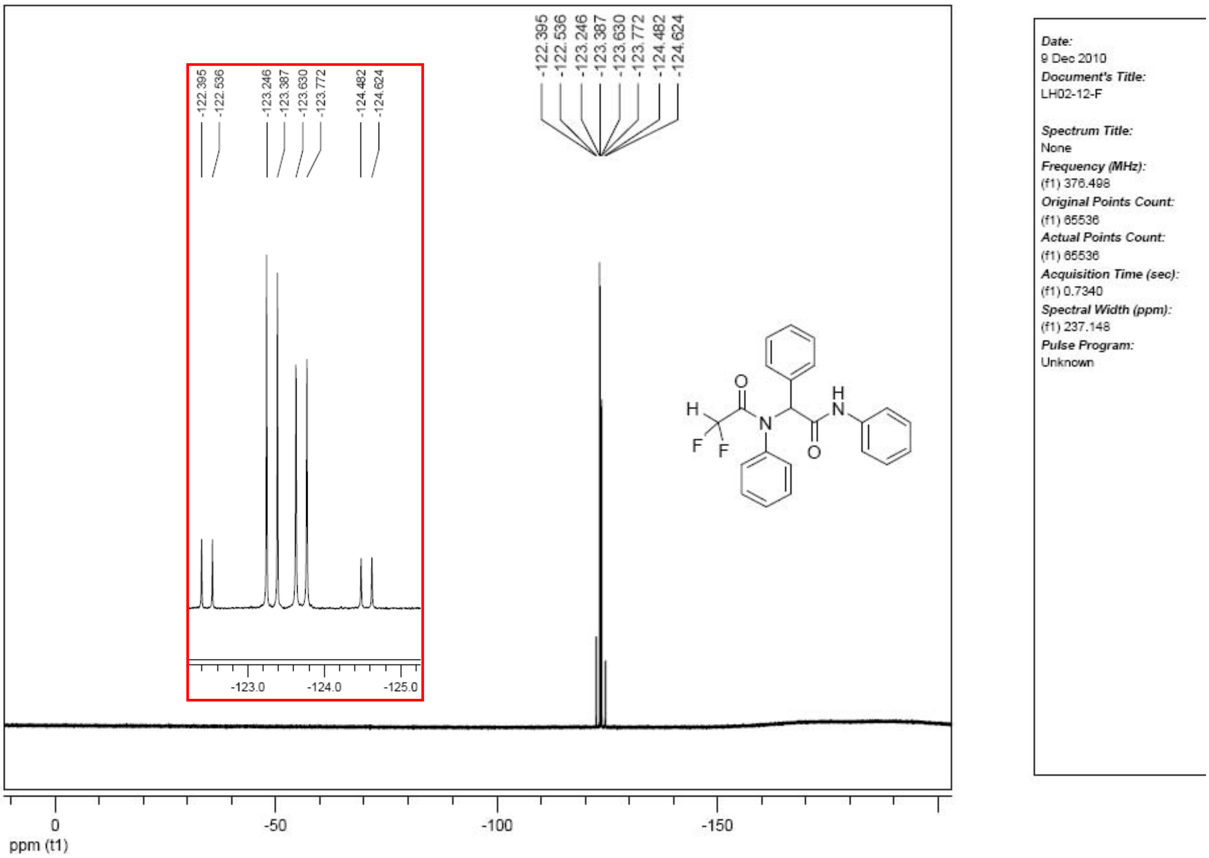

MS (ESI) of **4l**

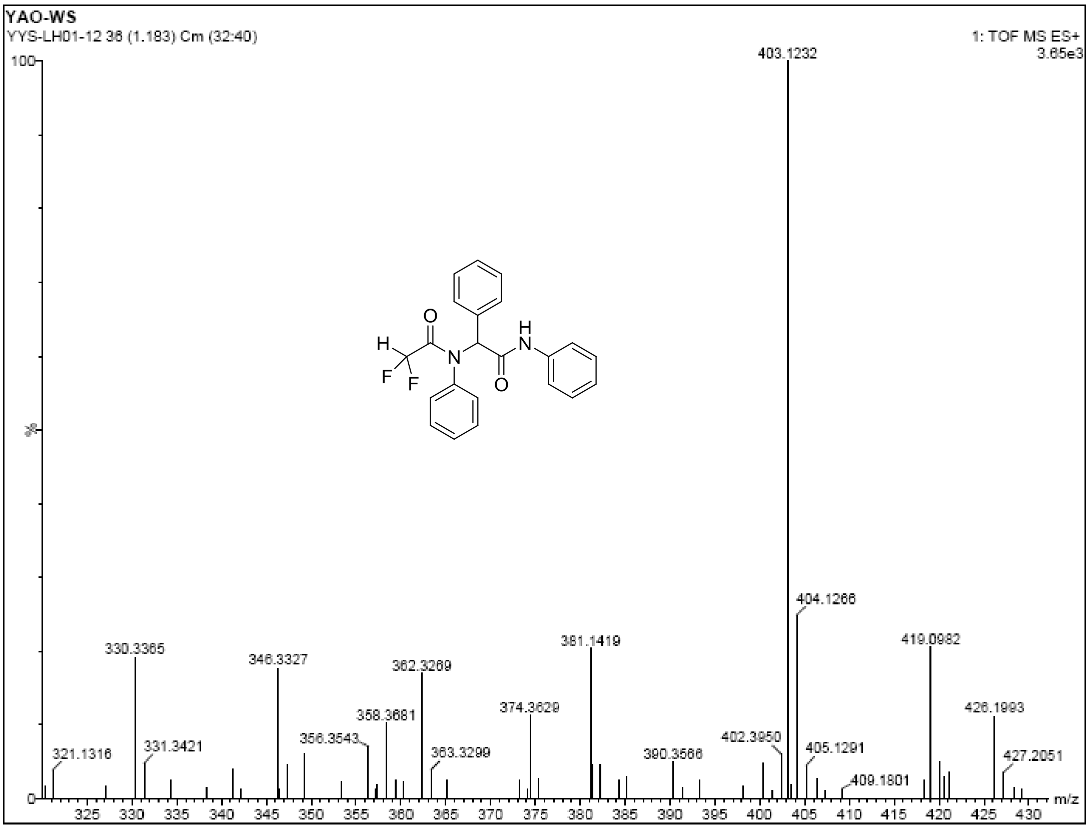

^{1}H NMR of **4m**

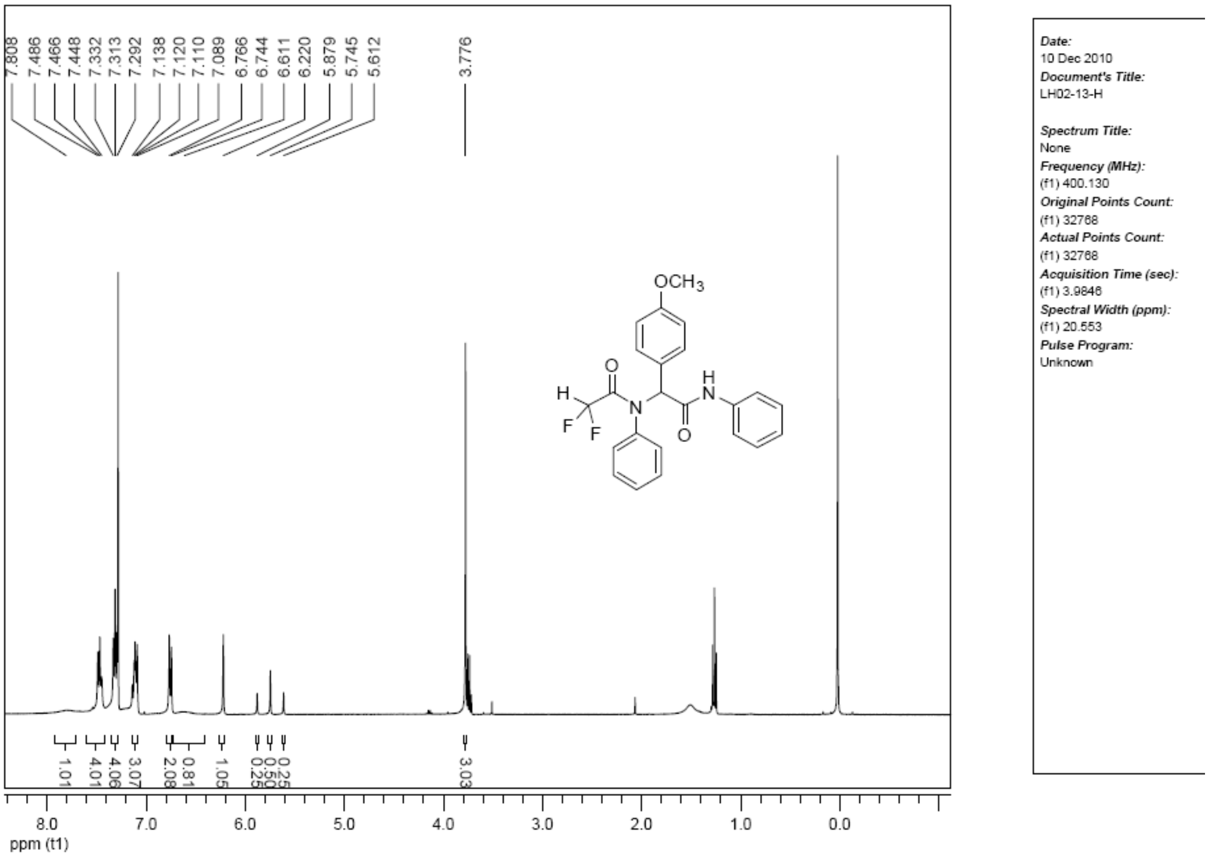

^{13}C NMR of **4m**

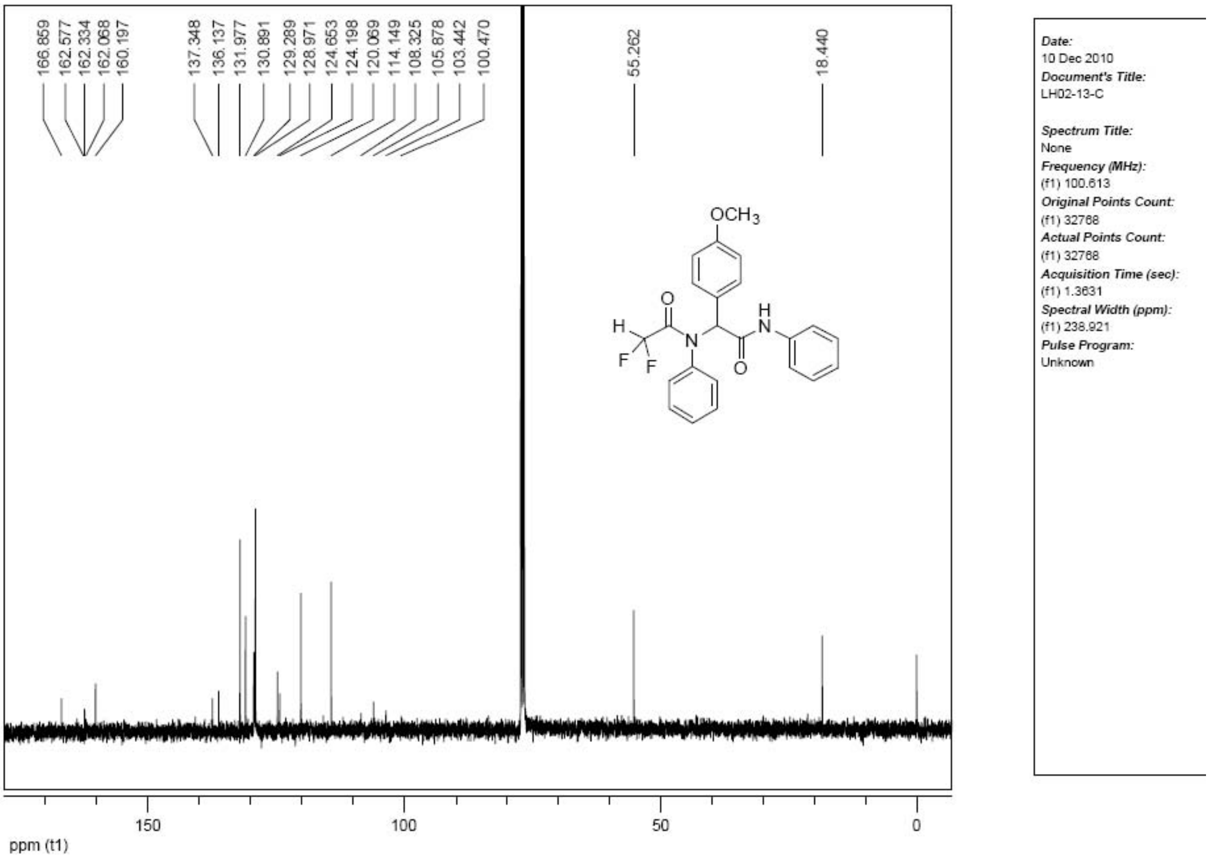

^{19}F NMR of **4m**

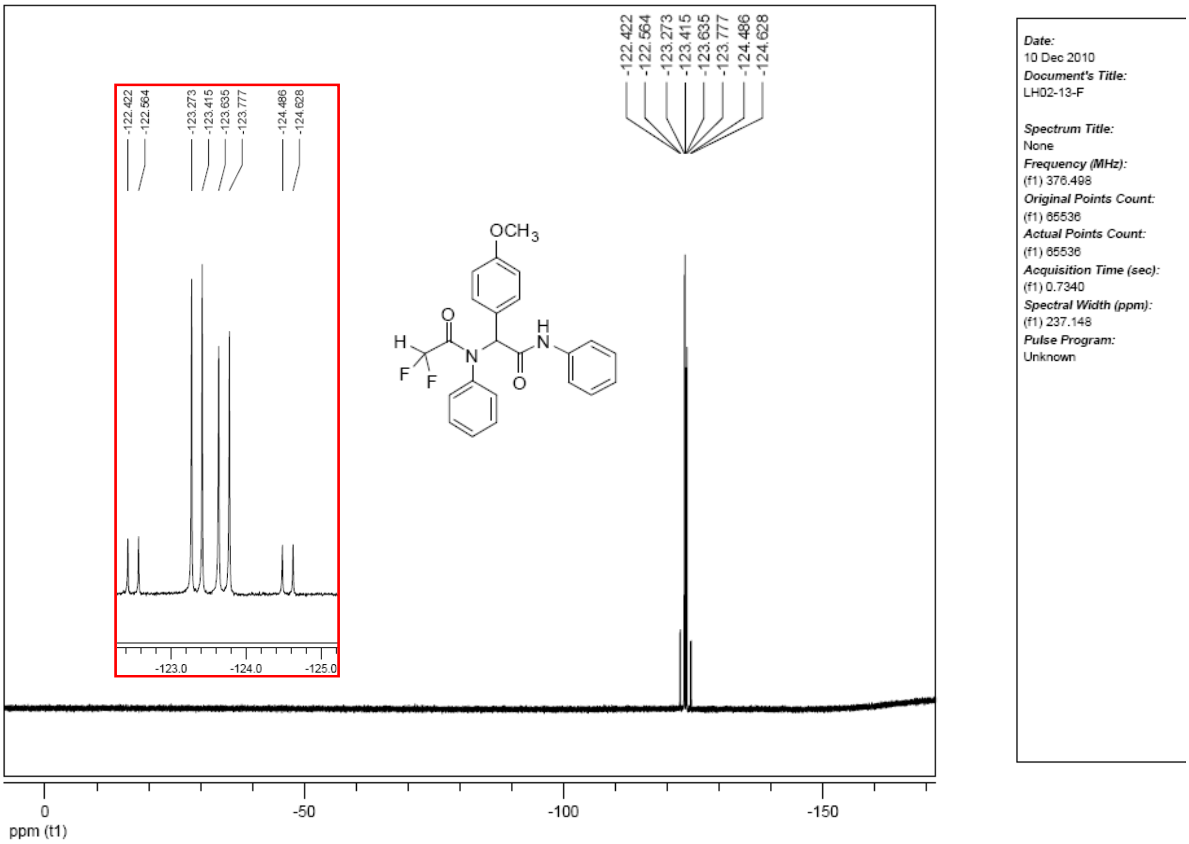

MS (ESI) of **4m**

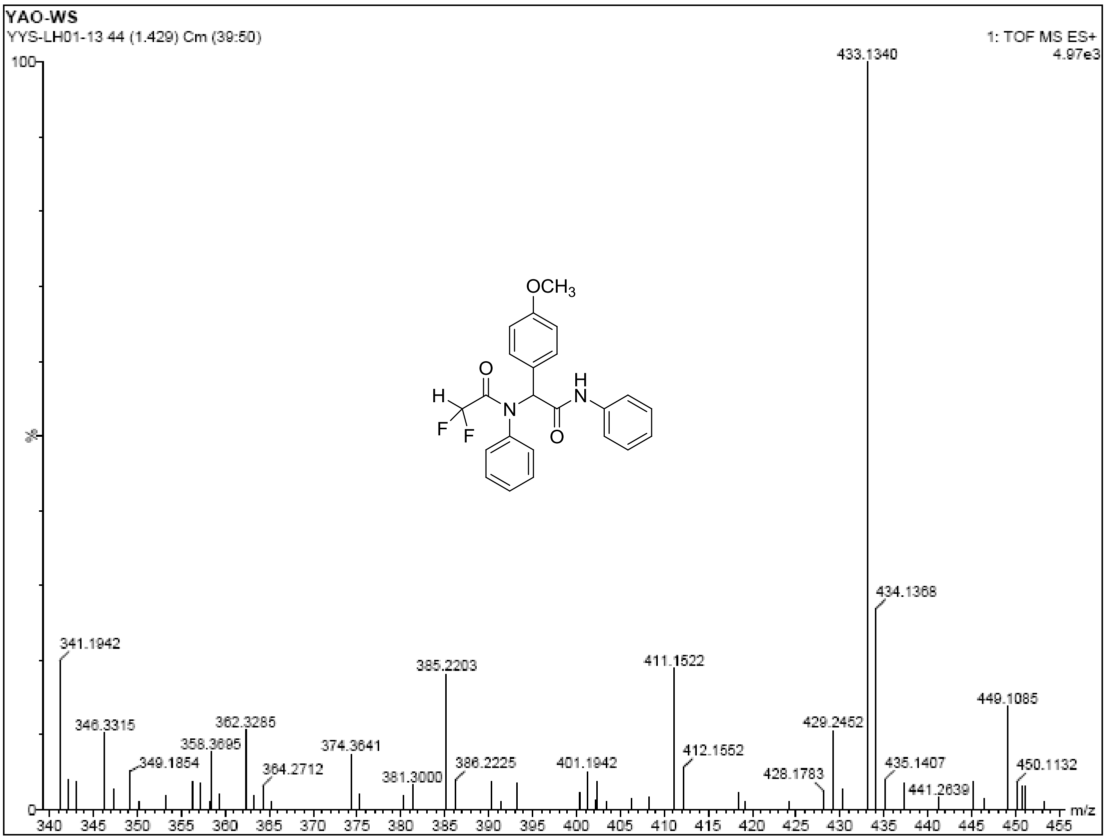

^{1}H NMR of **5**

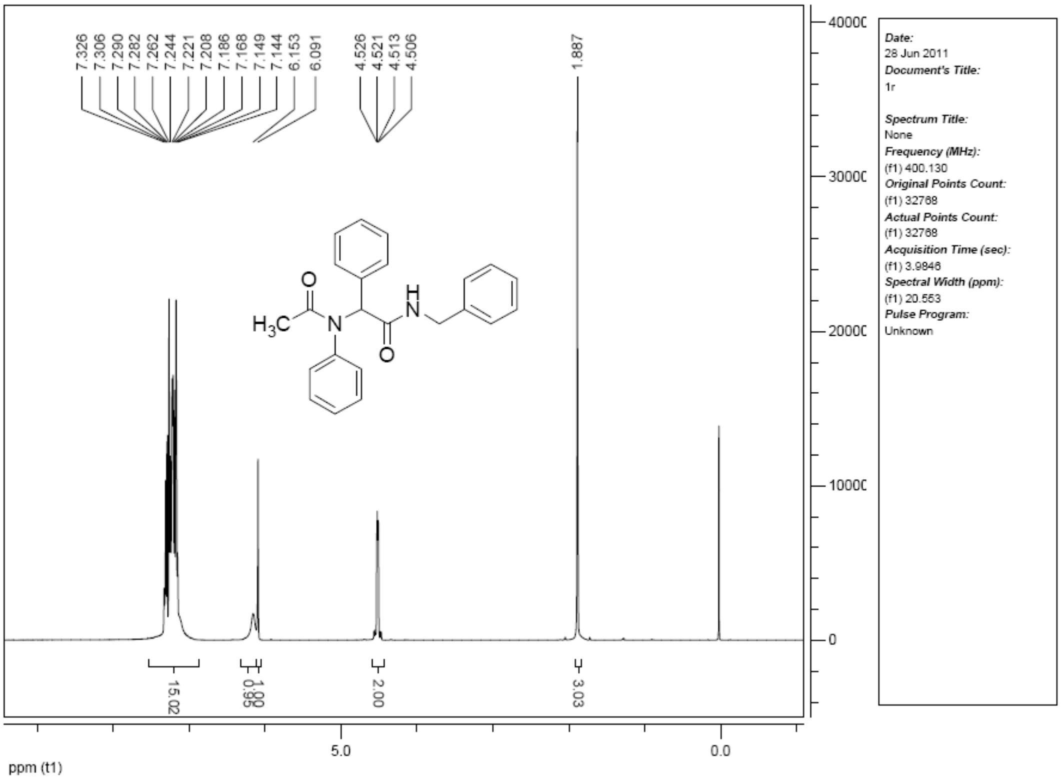

^{13}C NMR of **5**

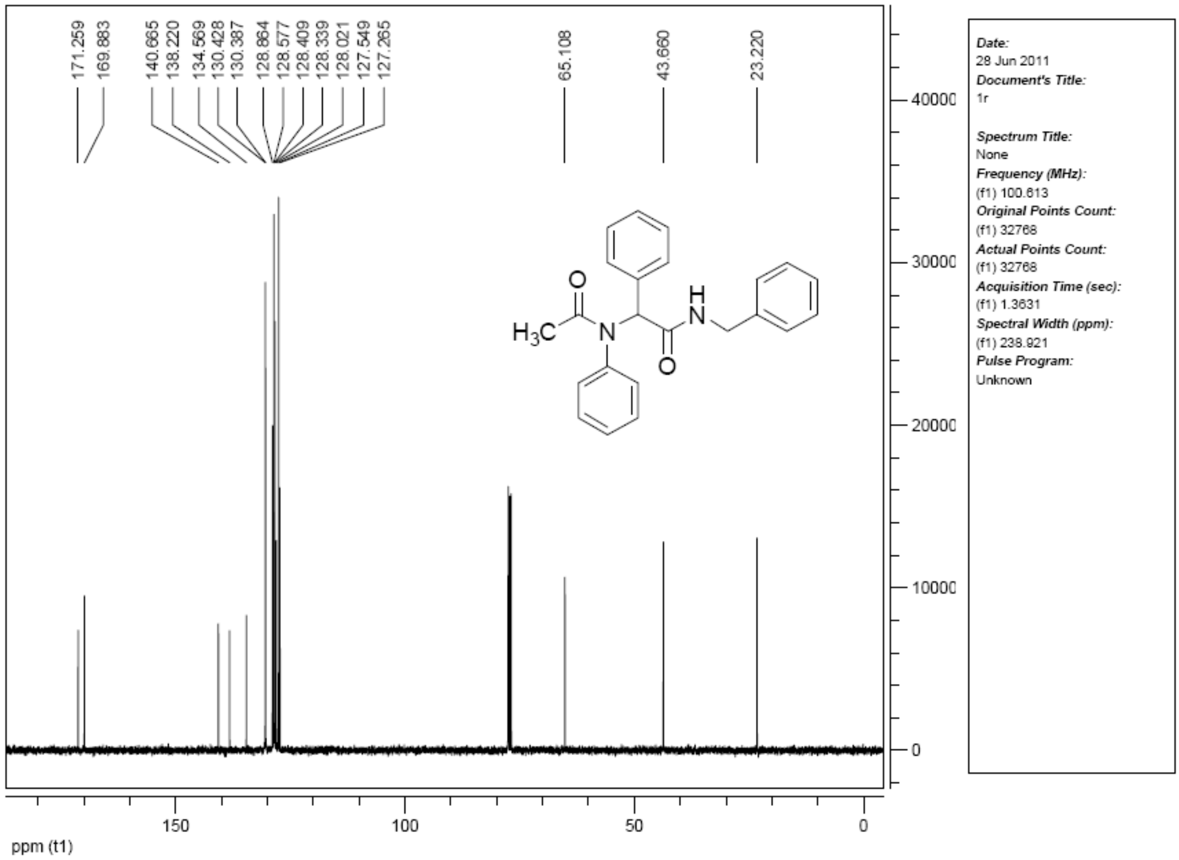

^{1}H NMR of **6**

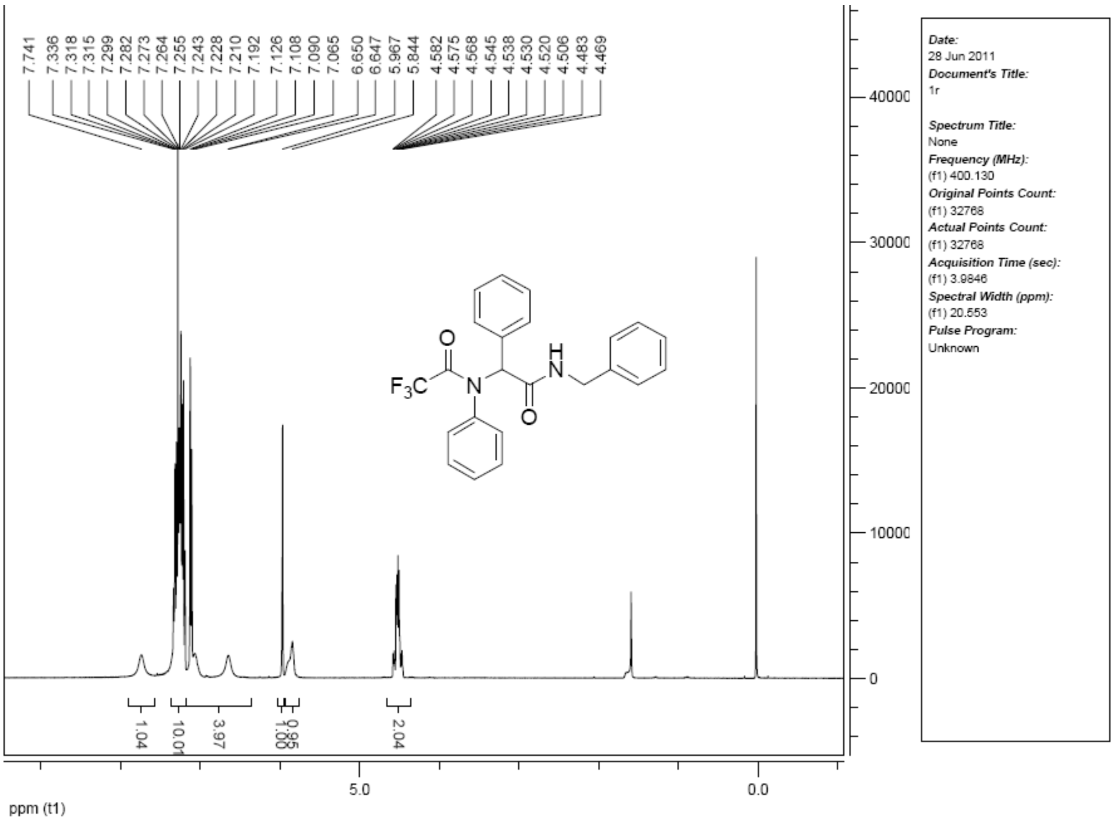

^{13}C NMR of **6**

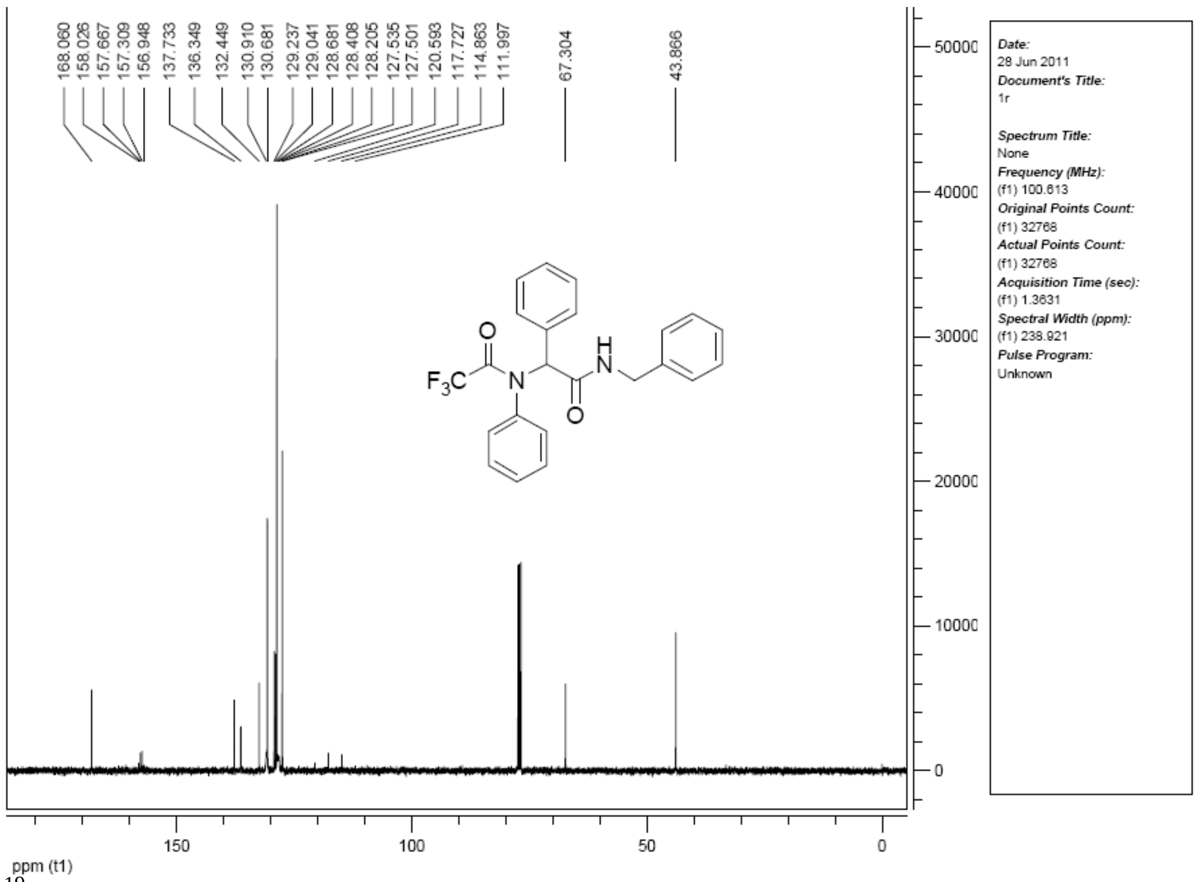

^{19}F NMR of **6**

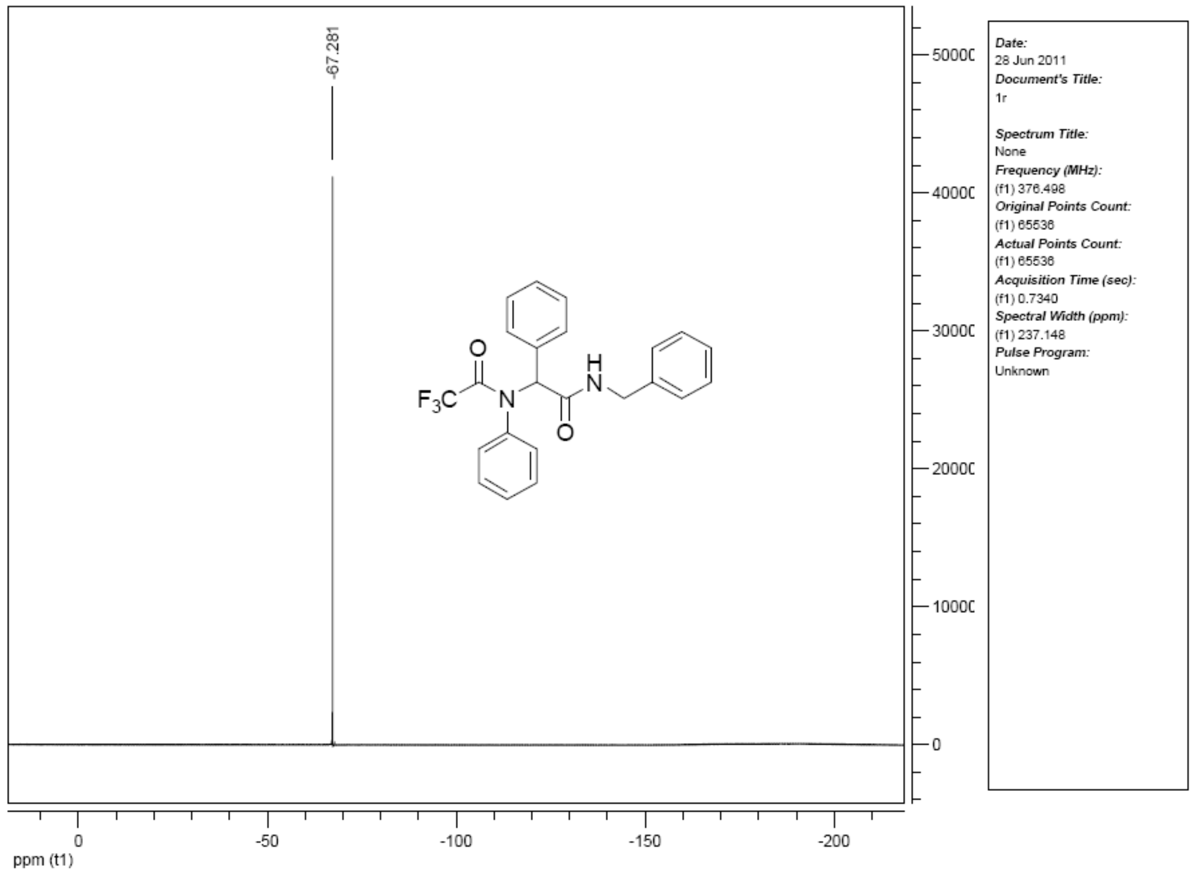

Supplement: File 1 — Experimental procedures and compound characterization. [file Beilstein_J_Org_Chem-07-1070-s001.pdf]
